# FAM3A drives uncoupling of muscle lipid accumulation and insulin resistance depending on insulin receptor

**DOI:** 10.1038/s41419-025-08298-1

**Published:** 2025-12-07

**Authors:** Dan Yang, Xiaohong Song, Xu Zeng, Zenghan Cao, Wenxuan Xiang, Xiangyu Xian, Lichai Yuan, Zheng Zhang, Yuehong Zheng

**Affiliations:** 1 Department of Vascular Surgery, Peking Union Medical College Hospital, Peking Union Medical College and Chinese Academy of Medical Sciences, Beijing, China; 2 Department of Computational Biology and Bioinformatics, Institute of Medicinal Plant Development, Chinese Academy of Medical Sciences and Peking Union Medical College, Beijing, China; 3 State Key Laboratory of Complex Severe and Rare Diseases, Peking Union Medical College Hospital, Chinese Academy of Medical Sciences and Peking Union Medical College, Beijing, China; 4 National Infrastructures for Translational Medicine, Institute of Clinical Medicine, Peking Union Medical College Hospital, Peking Union Medical College and Chinese Academy of Medical Sciences, Beijing, China; 5 Department of Pathology, Institute of Basic Medical Sciences, Peking Union Medical College and Chinese Academy of Medical Sciences, Beijing, China

**Keywords:** Mechanisms of disease, Cardiovascular diseases

## Abstract

Obesity-associated insulin resistance (IR) is closely related to intramyocellular lipid accumulation in skeletal muscle. FAM3 metabolism regulating signaling molecule A (FAM3A) is expressed and secreted in almost all tissues. However, its biological roles and underlying mechanisms remain largely unknown. Here, we reported that abnormal lipid metabolism decreased the FAM3A level. To investigate the function of FAM3A, a transgenic mouse strain was generated, in which FAM3A protein was overexpressed systemically. Proteomic analyses revealed that proteins related to lipid metabolic processes, specifically fatty acid (FA) synthesis complex enzymes and adiponectin were upregulated in the skeletal muscles of the FAM3A-transgenic mice compared with those in the skeletal muscles of the wild-type control mice. Furthermore, a positive correlation between FAM3A and adiponectin levels was observed in patient plasma samples. FAM3A transgene or FAM3A injection in high-fat diet (HFD)-fed mice led to increased levels of muscular srebp1, fas, acc, and acly expression, De novo FA biosynthesis, and lipid accumulation compared with those in matched controls. However, FAM3A transgene or FAM3A injection suppressed IR and inflammation and promoted glucose consumption in HFD-fed mice. Moreover, these functions of FAM3A were attenuated by the inhibition of PPARα. Notably, the inhibition of the insulin receptor abolished the linkage of FAM3A and PPARα as well as their downstream metabolic effects. Taken together, these findings suggest that increasing FAM3A expression in the body may represent a unique opportunity to avoid IR in individuals with obesity induced by high lipid levels.

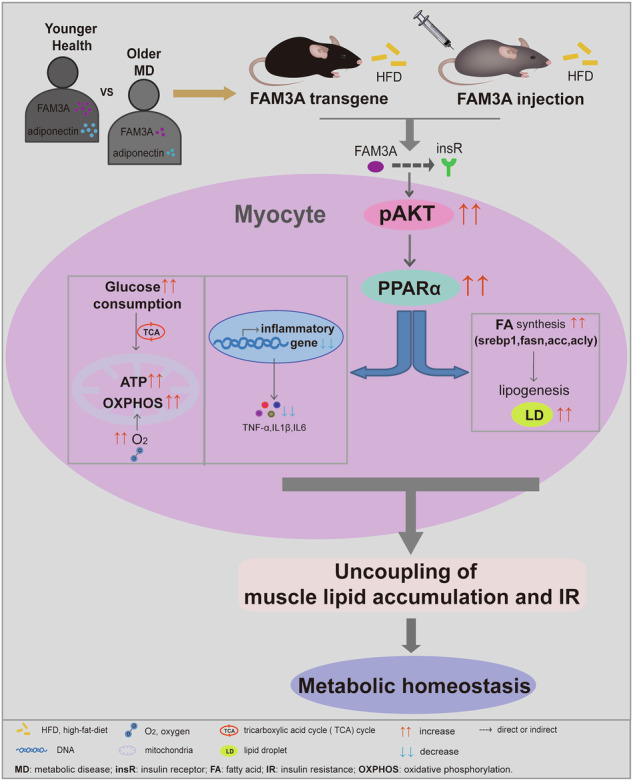

## Introduction

Family with sequence similarity 3 (FAM3) is a novel cytokine-like gene family identified in 2002 and includes four members designated FAM3 metabolism regulating signaling molecule A (FAM3A), FAM3B, FAM3C, and FAM3D [1]. FAM3A and FAM3C are expressed universally in almost all tissues; FAM3B is highly expressed in the pancreas; FAM3D is highly expressed in the placenta. To date, accumulating evidence has suggested that FAM3 proteins participate in the development of many human diseases, such as diabetes and cancer [2–4]. Although the current experimental evidence indicates that FAM3A is involved in regulating metabolism, oxidation, inflammation, and mitochondrial function, the biological roles of FAM3A and its underlying mechanisms remain largely unknown [5–7]. FAM3A is expressed in almost all tissues and is typically composed of 230 amino acid residues. Furthermore, FAM3A is an X chromosome-encoded gene with a large number of splice variants. The universal distribution of FAM3A in different tissues indicates that the biological function of FAM3A is extensive and not restricted to specific tissues or organs. FAM3A is expressed abundantly in the vascular endothelium, particularly capillaries, as immunohistochemistry has shown that stained FAM3A is expressed in the endocardium of the heart, as well as in the vascular endothelium in many other tissues [1]. Considering that the microvasculature is common in tissues and organs, this finding may explain the universal expression of FAM3A. Notably, the intracellular location of FAM3A is controversial. Some previous reports have indicated the localization of FAM3A in the mitochondria [8]. However, our previous study and another recent study revealed that FAM3A is a secreted protein that lacks a mitochondrial localization signal [9, 10].

Obesity, which is characterized by the accumulation of body fat due to chronic excessive energy intake against energy expenditure, is accompanied by systemic health problems, including insulin resistance (IR) and type 2 diabetes [11]. Moreover, in response to IR, the body initiates a defensive inflammatory response, namely, the release of numerous inflammatory cytokines and subsequent deterioration of organ function. This process is called low-grade inflammation, which is usually defined as “the chronic production, but a low-grade state, of inflammatory factors”. Peroxisome proliferator-activated receptor (PPAR) α is a transcription factor whose levels and activity are controlled at many different steps (e.g., gene expression and posttranslational modifications) and by many different factors (e.g., nutritional/hormonal cues, ageing, cytokines, and growth factors). PPARα target genes are involved not only in glucose and lipid metabolism but also in inflammation-modulating pathways. As it acts as a crossroad between inflammation and metabolism, PPARα is an important regulator of metabolic syndrome [12–14].

In the present study, we identified FAM3A as a critical regulator for the treatment of IR under conditions of high lipid-induced obesity. We explored the effects of FAM3A on high-fat diet (HFD)-induced metabolic disturbance and inflammation and elucidated the underlying mechanisms. We observed a positive correlation between FAM3A and adiponectin levels in patient plasma samples; although FAM3A increased intramyocellular lipid accumulation, it inhibited IR and metabolic disorders. Furthermore, these functions of FAM3A were due to the induction of PPARα and depended on the insulin receptor (insR). These findings reveal a pivotal role of FAM3A in and a novel mechanism underlying uncoupling intramyocellular lipid accumulation from IR and reshaping lipid metabolic homeostasis.

## Results

### Involvement of FAM3A in lipid metabolism

**Fig. 1:**
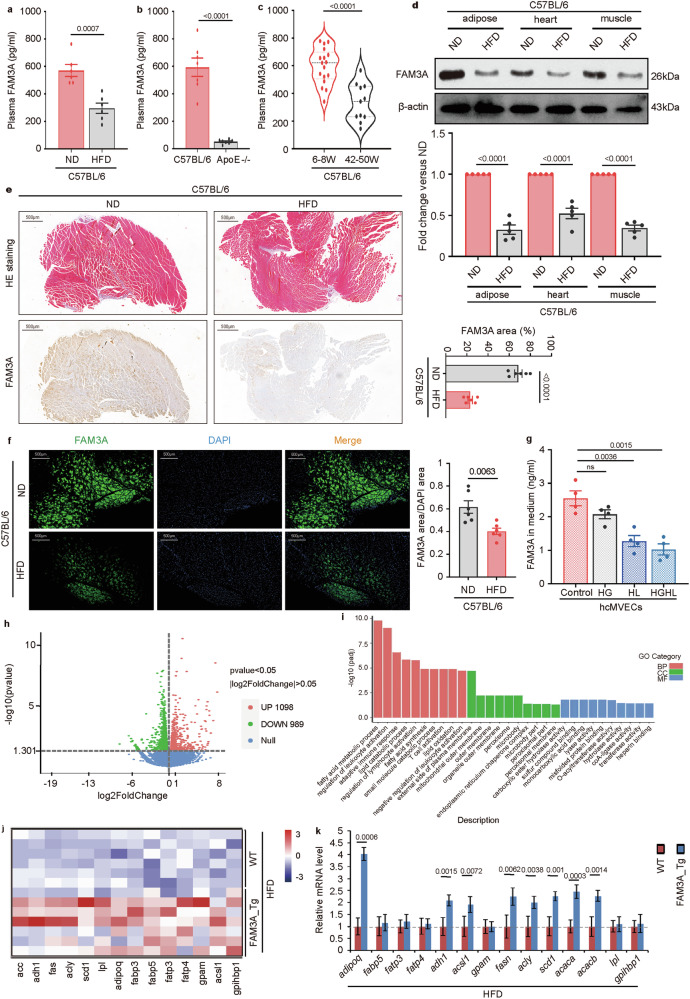
FAM3A is involved in lipid metabolism. **a–c** The plasma FAM3A concentrations in C57BL/6 mice fed a normal diet (ND) or a high-fat diet (HFD) for ten weeks (**a**, n = 6/group), in 6–8-week-old C57BL/6 fed a normal diet (ND) or high-fat diet supplemented with 1.25% cholesterol (HFD-chole; Researchdiet, #D12108c) for ten weeks (**b**, n = 7 biologically independent animals/group), and in 6–8-week-old (n = 17 biologically independent animals) or 42–50-week-old (n = 11 biologically independent animals) C57BL/6 mice (**c**). **d** Western blot image and quantification of the expression of FAM3A in soleus, heart, and adipose tissues from C57BL/6 mice fed a CD or an HFD for ten weeks (n = 5 biologically independent samples/group; quantitative comparisons between samples were run on the same gel). Representative images and quantification of immunohistochemical (**e**) and immunofluorescence (**f**) staining for the FAM3A protein in the soleus skeletal muscles from C57BL/6 mice fed a CD or an HFD for ten weeks (n = 6 biologically independent animals/group). Scale bar: 500 μm. **g** The commercially obtained human cardiac microvascular endothelial cells (hcMVECs) were cultured and treated with high glucose (HG, 33.3 mM D-glucose), high lipid (HL, 250 μM palmitate), or high glucose combined with high lipid (HGHL, 33.3 mM D-glucose and 250 μM palmitate) concentrations for 24 h. The FAM3A concentrations in the culture medium were detected using an ELISA kit (n = 4 biologically independent samples/group). **h** Volcano plot showing differentially expressed proteins based on proteomic data from mass spectrometry, which were determined in soleus muscle from the mice fed an HFD for ten weeks (biologically independent animals/group). **i** The functional enrichment analysis of Gene Ontology (GO) terms is shown. **j** The differentially expressed proteins involved in lipid metabolism are shown in the heatmap. **k** C57BL/6 wild-type (WT) mice and FAM3A-transgenic (FAM3A_Tg) mice were fed an HFD for 10 weeks and the transcript levels of crucial proteins were evaluated using qRT-PCR (n = 5 biologically independent animals/group). The data are presented as the means ± SEMs. Statistical significance was determined with a two-tailed independent *t* test, and *P* values are indicated (^ns^*P* ≥ 0.05). Source data are provided as a Source data file.

FAM3 family members have been reported to be active in metabolic processes. In our study, an HFD or an HFD added with cholesterol (HFD-chole) led to decreased plasma FAM3A levels in mice (Fig. 1a, b). Moreover, FAM3A was abundant in the plasma of normal C57BL/6 mice (Fig. 1a, b). We suspected that FAM3A may be associated with lipid metabolism. Because metabolic malfunction increases with age, we also detected plasma FAM3A levels in both young and old mice. The plasma FAM3A levels were significantly lower in old mice than in young controls (Fig. 1c). Furthermore, lower FAM3A protein levels were detected in the skeletal muscle of the soleus (Fig. 1d–f), heart (Fig. 1d), and adipose tissue (gonadal fat pad, Fig. 1d) from HFD-fed mice than in those from normal diet (ND)-fed controls. FAM3A is expressed predominantly in the vascular endothelium, particularly capillaries [1]. We therefore used microvascular endothelial cells (MVECs) to determine whether FAM3A expression was disturbed in the context of abnormal metabolism. As expected, treatment with both high lipid (HL) and high glucose combined with high lipid (HGHL) decreased the FAM3A levels in the medium from cultured human cardiac microvascular endothelial cells (hcMVECs), whereas high glucose (HG) treatment did not significantly affect the FAM3A levels (Fig. 1g). Collectively, these findings suggest that FAM3A expression is reduced under abnormal lipid metabolic conditions.

We obtained a comprehensive understanding of the biological function of FAM3A by analyzing proteomic data from mass spectrometry to assess the main roles of FAM3A in muscle tissues from C57BL/6 WT control mice and genetically modified FAM3A-transgenic (FAM3A_Tg) mice (Supplementary Fig. 1) fed an HFD. The results showed that an array of proteins was differentially regulated by FAM3A (Fig. 1h), and the functional enrichment analysis based on Gene Ontology (GO) revealed that FAM3A may play crucial roles in lipid metabolism, such as fatty acid metabolism (Fig. 1i). Specifically, these lipid metabolism-related proteins were involved in lipid biosynthesis, including fatty acid synthetase (fas), ATP-citrate lyase (acly), stearoyl-CoA desaturase 1 (scd1), and acetyl-CoA carboxylase (acc) (Fig. 1j). The lipid metabolism-related proteins whose expression changed the most were collected, and their transcript levels were further assessed by qRT-PCR. Compared with the levels in WT controls, the transcript levels of *adipoq*, *fasn*, *acly*, *acaca/acc1*, and *acacb/acc2* in muscles were significantly upregulated in FAM3A-transgenic mice (Fig. 1k). Additionally, the analysis of metabolic pathways showed that the differentially expressed proteins were enriched in the biological function of fatty acid biosynthesis (Supplementary Fig. 2). Therefore, these data suggest that FAM3A may regulate lipogenesis in muscle tissues.

### FAM3A increases lipid accumulation in muscles

**Fig. 2:**
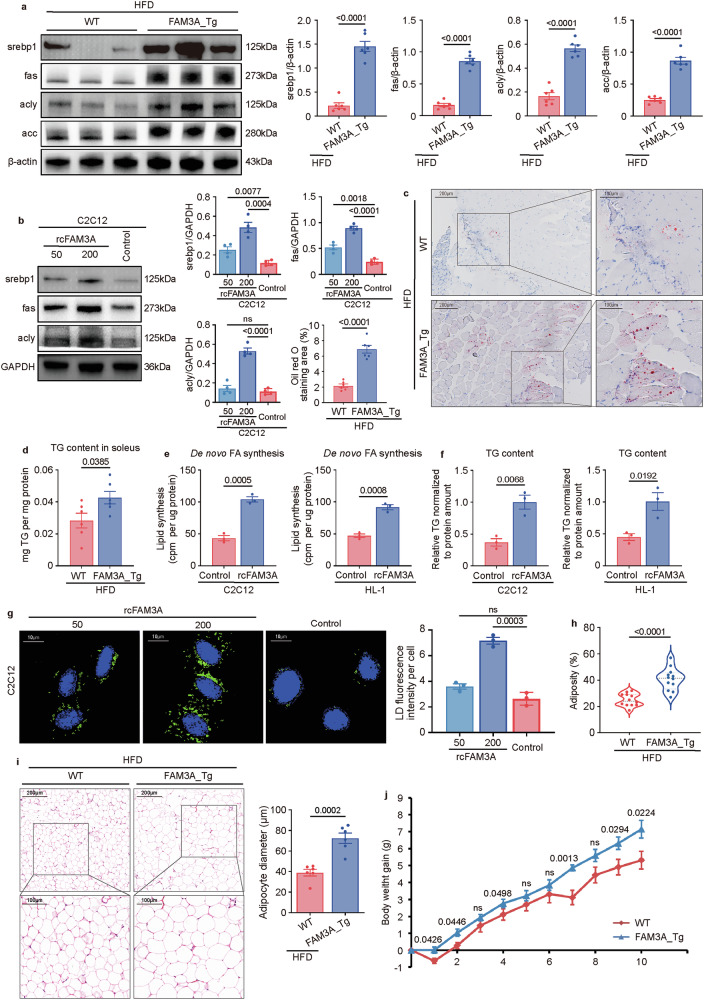
FAM3A increases the lipid content. **a** Western blot images and quantification of the expression levels of srebp1, fas, acly, and acc in soleus muscles from wild-type (WT) mice and FAM3A-transgenic (FAM3A_Tg) mice fed an HFD for ten weeks (n = 6 biologically independent animals/group; quantitative comparisons between samples were performed on the same gel). **b** Western blot images and quantification of the expression levels of srebp1, fas, and acly in cells treated with different concentrations (ng/ml) of rcFAM3A for 8 h (n = 4 biologically independent samples/group; quantitative comparisons between samples were run on the same gel). The lipid droplets (LDs) detected by oil red O staining (**c**) and TG contents (**d**) were measured in soleus muscles from WT mice and FAM3A_Tg mice fed an HFD for five weeks (n = 6 biologically independent animals/group) and plotted in graphs. Scale bar: 200 μm, insets: 100 μm in (**c**). **e**–**g** C2C12 or HL-1 cells were treated with recombinant FAM3A (rcFAM3A, 200 ng/ml) for 12 h. Cellular De novo FA synthesis (**e**), TG contents (**f**), and LDs (**g**) were measured and graphed (n = 3 biologically independent samples/group). Scale bar: 10 μm in (**g**). Body adiposity (**h**, n = 12 biologically independent animals/group), adipocyte diameter in gWAT (**i**, n = 6 biologically independent animals/group), and body weight gain (**j**, n = 10 biologically independent animals/group) were measured in mice fed an HFD for 10 weeks and plotted. The data are presented as the means ± SEMs. Statistical significance was determined with a two-tailed independent *t* test, and *P* values are indicated (^ns^*P* ≥ 0.05). Source data are provided as a Source data file.

Next, we explored the role of FAM3A in lipid biogenesis. As detected by Western blot analysis of skeletal muscles, the levels of the key lipogenic regulator sterol regulatory element-binding protein 1 (srebp1) and lipogenic enzymes including fas, acly, and acc, were upregulated in FAM3A-transgenic mice (Fig. 2a) or in mice injected with the recombinant FAM3A polypeptide (Supplementary Fig. 3a). In vitro experiments also confirmed the ability of FAM3A to increase the levels of srebp1, fas, and acly in C2C12 cells (Fig. 2b). Furthermore, compared with those in the matched controls, the lipid droplet (LD) detected by oil red O staining (Fig. 2c and Supplementary Fig. 3b) and triglyceride (TG) abundance (Fig. 2d and Supplementary Fig. 3c) in the soleus were increased in both the FAM3A-transgenic and the rcFAM3A-injected mice. The ability of FAM3A to promote lipogenesis was further confirmed in vitro, as rcFAM3A increased De novo FA synthesis (Fig. 2e), the TG content (Fig. 2f), and LD area (Fig. 2g) in both C2C12 and HL-1 cells. Moreover, both FAM3A-transgenic and rcFAM3A-injected mice had elevated body adiposity rates (Fig. 2h and Supplementary Fig. 3d, g), which were accompanied by an increased adipocyte diameter (Fig. 2i and Supplementary Fig. 3e) and weight of inguinal white adipose tissue (iWAT) and gonadal white adipose tissue (gWAT) (Supplementary Fig. 3f). Furthermore, compared with their matched controls, FAM3A-transgenic mice experienced much greater weight gain (Fig. 2j) in compared to their matched controls. Taken together, these findings suggest that FAM3A may promote lipid anabolism in muscles.

### FAM3A levels are positively correlated with adiponectin levels

**Fig. 3:**
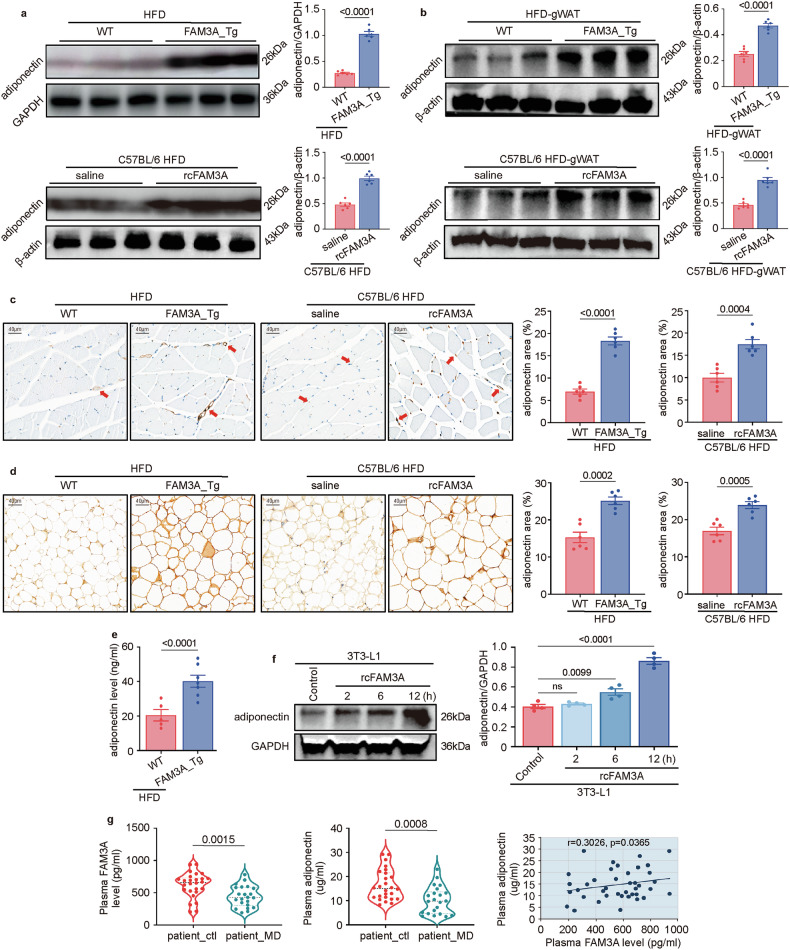
FAM3A induces adiponectin expression. Western blot images and quantification of the adiponectin expression levels in soleus tissues (**a**) and gWATs (**b**) from WT and FAM3A_Tg mice fed an HFD for 10 weeks and from the C57BL/6 mice fed an HFD for ten weeks and treated with or without rcFAM3A (n = 6 biologically independent animals/group; quantitative comparisons between samples were performed on the same gel). Representative images and quantification of immunohistochemical staining for the adiponectin protein in soleus skeletal muscles (**c**) and gWATs (**d**) from mice treated as described in a (n = 6 biologically independent animals/group). Scale bar: 40 μm. **e** Elisa results showing the quantification of adiponectin levels in soleus skeletal muscles in mice treated as described in a (n = 5 or n = 7 biologically independent WT or FAM3A_Tg mice, respectively). **f** Western blot images and quantification of the expression levels of adiponectin in 3T3-L1 cells treated with or without rcFAM3A (200 ng/ml) for 2–12 h (n = 4 biologically independent samples/group; quantitative comparisons between samples were run on the same gel). **g** The plasma FAM3A and adiponectin levels in patients with varicosity with metabolic disorder (MD), including diabetes, hyperlipidaemia, or obesity (patient_MD, n = 22), or without MD (patient_ctl, n = 26), and linear regression models are shown. Source data are provided as a Source data file.

As an adipocyte-derived molecule, adiponectin is associated with metabolic disease. Our high-throughput results revealed that the level of adiponectin was significantly influenced by FAM3A (Fig. 1j, k). Furthermore, our previous study indicated that the level of diponectin was increased by FAM3A treatment [10]. We therefore explored the link between FAM3A and adiponectin. Both the FAM3A transgene in mice and injection of the recombinant polypeptide significantly increased adiponectin levels in soleus skeletal muscles (Fig. 3a, c, e) and in gWATs (Fig. 3b, d). Additionally, this finding was confirmed by in vitro experiments in which recombinant FAM3A (rcFAM3A) increased adiponectin levels in 3T3-L1 adipocytes (Fig. 3f). We also investigated whether a linear correlation existed between the levels of FAM3A and adiponectin in clinical patients. By measuring the plasma FAM3A and adiponectin levels in patients with varicosity with or without metabolic disease (MD; hyperlipidaemia, diabetes, and obesity), we observed a positive correlation between the levels of FAM3A and adiponectin (Fig. 3g). Moreover, compared with non-MD controls, patients with varicosity along with MD had lower plasma levels of FAM3A and adiponectin (Fig. 3g).

### FAM3A ameliorates HFD-induced inflammation and metabolic disturbances

**Fig. 4:**
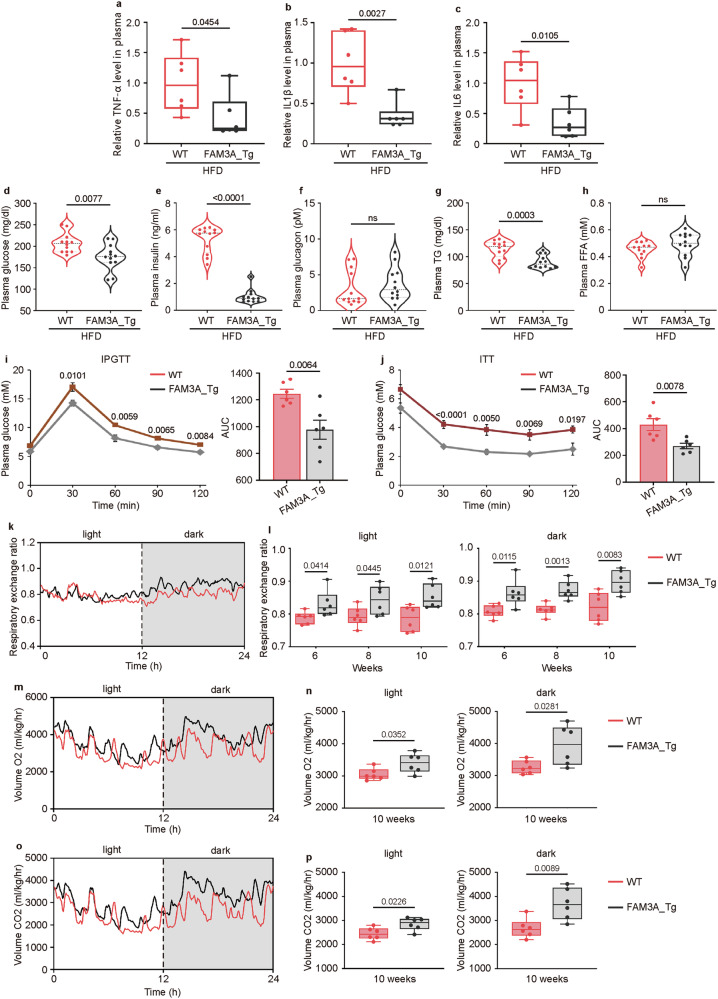
Metabolic and inflammatory characteristics of FAM3A-transgenic mice. WT mice and FAM3A_Tg mice were fed an HFD for sixteen weeks. The TNF-α (**a**), IL1β (**b**), and IL6 (**c**) levels in soleus muscles were measured and graphed (n = 6 biologically independent animals/group in **a**–**c**). The plasma fasting glucose (**d**), insulin (**e**), glucagon (**f**), triglyceride (TG, **g**), and free fatty acid (FFA, **h**) levels were measured and graphed (n = 12 biologically independent animals/group). Intraperitoneal glucose tolerance tests (IPGTTs, **i**) and insulin tolerance tests (ITTs, **j**) were performed (n = 6 biologically independent animals/group). The respiratory exchange ratio (**k**, **l**) and the respiratory volumes of O_2_ (**m**, **n**) and CO_2_ (**o**, **p**) were measured and graphed (n = 6 biologically independent animals/group). The data are presented as the means ± SEMs. Statistical significance was determined with a two-tailed independent *t* test, and *P* values are indicated (^ns^*P* ≥ 0.05). Source data are provided as a Source data file.

Long-term lipotoxicity is closely linked to chronic inflammation, which is a crucial pathological step in metabolic disease progression. We therefore investigated whether FAM3A influenced the inflammatory status under HFD-fed conditions. The overexpression of FAM3A attenuated the levels of TNF-α, IL1β, and IL6 in vivo (Fig. 4a–c and Supplementary Fig. 4a–c) and in vitro (Supplementary Fig. 4d–i). Additionally, we explored the potential plasma cytokines controlled by FAM3A in FAM3A-transgenic mice using cytokine antibody arrays (RayBiotech, USA; GSM-CAA-4000). As shown in Supplementary Table 3, FAM3A significantly altered the levels of some circulating biomarkers in mice fed an HFD.

The attenuation of inflammation is beneficial for enhancing glucose use and promoting insulin sensitivity [15–17]. We further investigated metabolic homeostasis in FAM3A-transgenic mice and rcFAM3A-infused mice. Both the FAM3A-transgenic mice and the rcFAM3A-infused mice had lower plasma glucose concentrations (Fig. 4d and Supplementary Fig. 4j) and insulin levels (Fig. 4e and Supplementary Fig. 4k) than their matched controls. Furthermore, glucose tolerance improved (Fig. 4i), and insulin sensitivity increased (Fig. 4j) in FAM3A-transgenic mice. However, the plasma glucagon levels did not change significantly (Fig. 4f and Supplementary Fig. 4l). We also investigated whether FAM3A affected plasma lipid levels. Compared with WT control mice, FAM3A-transgenic mice presented lower plasma TG levels in (Fig. 4g). However, the plasma free FA (FFA) levels did not change significantly (Fig. 4h and Supplementary Fig. 4n).

We further measured the respiratory exchange ratio (RER) to assess carbohydrate consumption. As expected, the respiratory exchange ratios in both the FAM3A-transgenic mice and the rcFAM3A-infused mice were significantly higher than those in the matched controls during both the light period (a period of increased lipid utilization) and the dark period (a period of increased carbohydrate utilization) (Fig. 4k–p and Supplementary Fig. 4o, p), suggesting that FAM3A may induce an increase in glucose consumption.

Taken together, these results suggest that FAM3A may be beneficial to metabolic homeostasis and exhibit anti-inflammatory activity.

### FAM3A promotes mitochondrial function and maintains muscle homeostasis

**Fig. 5:**
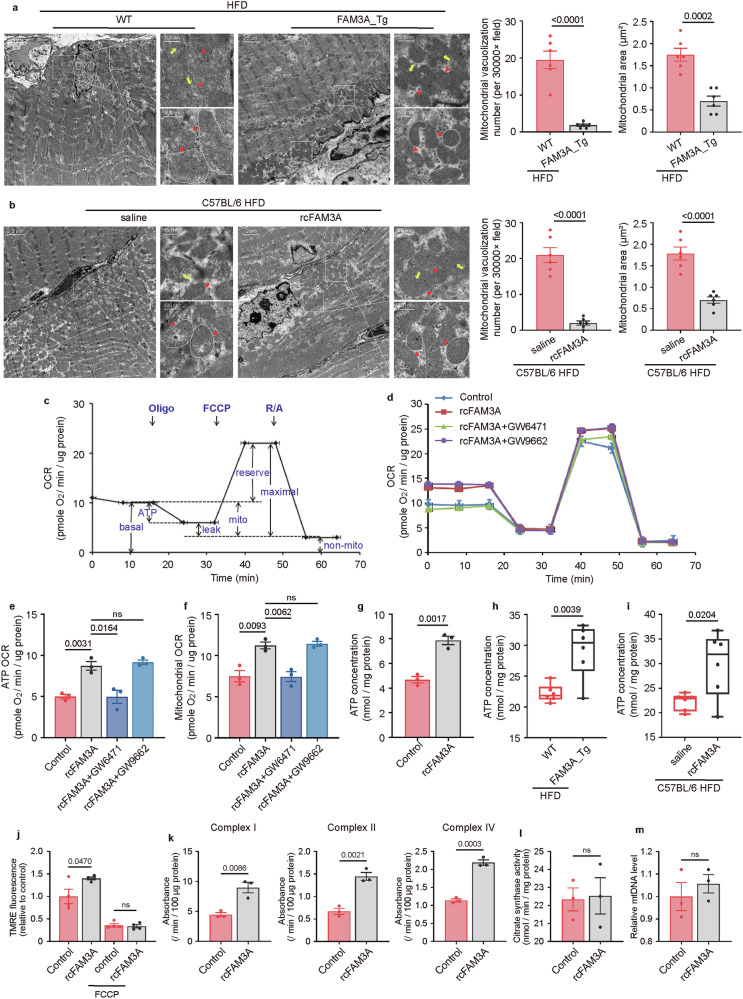
Mitochondrial function and biogenesis in muscle following FAM3A interference. **a**, **b** The electron microscope images of the mitochondrial morphology are shown, and mitochondrial vacuolization and area in the soleus muscles of the FAM3A_Tg and rcFAM3A-infused mice fed an HFD for ten weeks are shown in the graph (n = 6 biologically independent animals/group). Scale bar: 2 μm, insets: 500 nm. Yellow arrows indicate mitochondrial cristae, red arrows indicate vacuolization, and red arrows combined with circles indicate swelling. **c**–**f** C2C12 cells were treated with rcFAM3A (200 ng/ml) for 8 h. Schematic diagram of the oxygen consumption rate (OCR) trace of the mitochondrial flux assay are shown (**c**). The cellular OCRs are shown (**d**). The ATP OCR (**e**) and mitochondrial OCR (**f**) were quantified and graphed (n = 3 biologically independent samples/group). **g**–**i** ATP concentrations were measured and graphed in cells treated as described in (**c**) (**g**, n = 3 biologically independent samples/group) and in the soleus muscles of mice fed an HFD for ten weeks (**h**, **i**; n = 6 biologically independent animals/group). Mitochondrial membrane potential (**j**), complex I, II, and IV activity (**k**), citrate synthase activity (**l**), and mtDNA level (**m**) were measured and graphed in C2C12 cells treated with rcFAM3A (200 ng/ml) for 8 h (n = 3 biologically independent samples/group). The data are presented as the means ± SEMs. Statistical significance was determined with a two-tailed independent *t* test, and *P* values are indicated (^ns^*P* ≥ 0.05). Source data are provided as a Source data file.

Previous studies have reported a role of FAM3A in mitochondrial ATP production. We thus explored the effects of FAM3A on mitochondrial homeostasis. Transmission electron microscope (TEM) images showed fewer mitochondrial vacuoles, cristae rupture, and swellings in both FAM3A-transgenic mice and rcFAM3A-injected mice than in their matched controls under HFD-fed conditions (Fig. 5a, b), suggesting that FAM3A protects against mitochondrial injury in vivo. We also assessed mitochondrial function in vitro and found that FAM3A promoted ATP-related OCR, mitochondrial OCR, and maximal OCR in C2C12 cells (Fig. 5e, f and Supplementary Fig. 5a), but did not significantly affect the proton leak OCR in vitro (Supplementary Fig. 5b). Treatment of C2C12 cells with FAM3A increased ATP levels (Fig. 5g). Similarly, the increase in FAM3A levels in vivo led to increased ATP generation (Fig. 5h, i). Moreover, compared with control cells, FAM3A-treated C2C12 cells displayed a higher mitochondrial membrane potential (Fig. 5j). In addition, we also assessed the effects of FAM3A on citrate synthase activity and mitochondrial DNA (mtDNA) levels in vitro and observed that rcFAM3A did not affect these phenotypes or functions in C2C12 cells (Fig. 5l, m). Therefore, mitochondrial biogenesis was not the main mechanism underlying the regulatory effects of FAM3A on mitochondrial function. Interestingly, respiratory chain enzyme activity assays in rcFAM3A-treated C2C12 cells showed that the functions of the enzymes in complex I, II, and IV were significantly increased (Fig. 5k), possibly leading to increased mitochondrial OCR and ATP production.

Since a previous study reported a role of FAM3A in angiogenesis [18], we investigated whether the increased mitochondrial OCR was matched by elevated blood flux and blood oxygen supply. As expected, FAM3A transgene or injection in mice enhanced blood perfusion in the hind limb (Supplementary Fig. 6a, b), as well as blood hemoglobin oxygen saturation (Supplementary Fig. 6c). Moreover, the increase in FAM3A levels in mice was beneficial to the intactness of muscle morphology as manifested by the much clearer and more regular Z-disks in these mice than in control mice (Supplementary Fig. 6d, e). Interestingly, myosin heavy chain (*myh*) expression levels in the soleus of FAM3A-transgenic mice and FAM3A-infused mice were higher than those in the matched controls (Supplementary Fig. 6f, g).

### PPARα is involved in the uncoupling of muscle lipid accumulation and insulin resistance by FAM3A

**Fig. 6:**
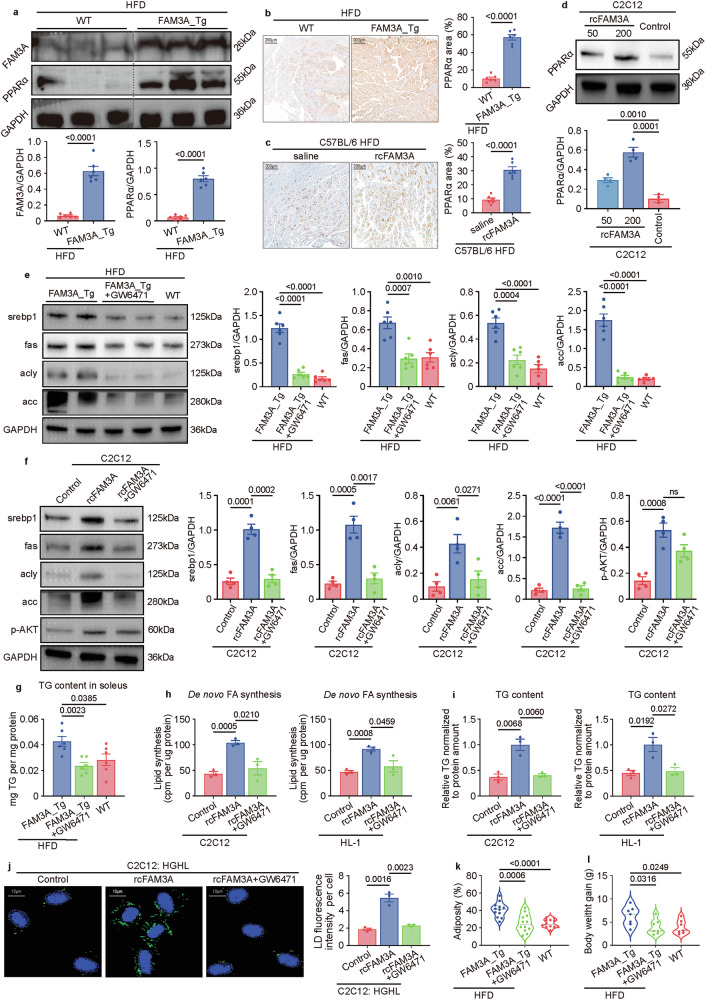
FAM3A drives lipogenesis in muscles via PPARα. **a** Western blot images and quantification of the expression levels of FAM3A and PPARα in soleus muscles from WT mice and FAM3A_Tg mice fed an HFD for ten weeks (n = 6 biologically independent animals/group; quantitative comparisons between samples were run on the same gel). **b**, **c** Representative images and quantification of immunohistochemistry staining for the PPARα protein in soleus skeletal muscles from FAM3A_Tg mice and rcFAM3A-infused mice (n = 6 biologically independent animals/group). Scale bar: 200 μm. **d** Western blot images and quantification of the expression level of PPARα in cells treated with different concentrations (ng/ml) of rcFAM3A for 8 h (n = 4 biologically independent samples/group; quantitative comparisons between samples were run on the same gel). **e** Western blot images and quantification of the expression levels of the indicated proteins in soleus muscles from mice treated with GW6471 and fed an HFD for 10 weeks (n = 6 biologically independent animals/group; quantitative comparisons between samples were run on the same gel). **f** Western blot images and quantification of the expression levels of the indicated proteins in cells pre-treated with GW6471 and then stimulated with rcFAM3A (200 ng/ml; n = 4 biologically independent samples/group; quantitative comparisons between samples were run on the same gel). **g** TG content was measured in soleus muscles from mice fed an HFD for five weeks and the results were plotted (n = 6 biologically independent animals/group). **h**–**j** C2C12 or HL-1 cells were treated with rcFAM3A (200 ng/ml) for 12 h. Cellular De novo FA synthesis (**h**), TG content (**i**), and LD content (**j**) were measured and graphed (n = 3 biologically independent samples/group). **k**, **l** Body adiposity and body weight gain were measured in mice fed an HFD for 10 weeks and the results were plotted (n = 12 or 7 biologically independent animals/group in **k** or **l**, respectively). The data are presented as the means ± SEMs. Statistical significance was determined with a two-tailed independent *t* test, and *P* values are indicated (^ns^*P* ≥ 0.05). Source data are provided as a Source data file.

We further explored the underlying mechanisms involved in the biological function of FAM3A. PPARα is a crucial PPAR isoform expressed in skeletal muscle tissues. In this study, we focused on PPARα. As detected by Western blot and immunohistochemical staining, the levels of PPARα protein in the soleus muscles were increased in the FAM3A-transgenic mice compared with the WT control mice (Fig. 6a–c). An in vitro study also confirmed the role of FAM3A in increasing PPARα protein levels in C2C12 cells (Fig. 6d). We therefore investigated whether the metabolic regulatory effects of FAM3A were due to increased PPARα signaling. As expected, both the in vivo and in vitro results showed that the inhibition of PPARα by GW6471 abolished FAM3A-induced increase in lipogenic protein expression in the muscle (Fig. 6e, f). Furthermore, the increased LD area detected by oil red O staining (Supplementary Fig. 7) and increased triglyceride (TG) levels (Fig. 6g) in the soleus muscles were suppressed by GW6471. These effects of GW6471 were also assessed in vitro, and the rcFAM3A-induced increases in De novo FA synthesis (Fig. 6h), TG content (Fig. 6i), and LD area (Fig. 6j) were repressed by treatment with GW6471 in C2C12 and HL-1 cells. Moreover, similar trends were observed for the body adiposity rate (Fig. 6k) and body weight gain (Fig. 6l).

**Fig. 7:**
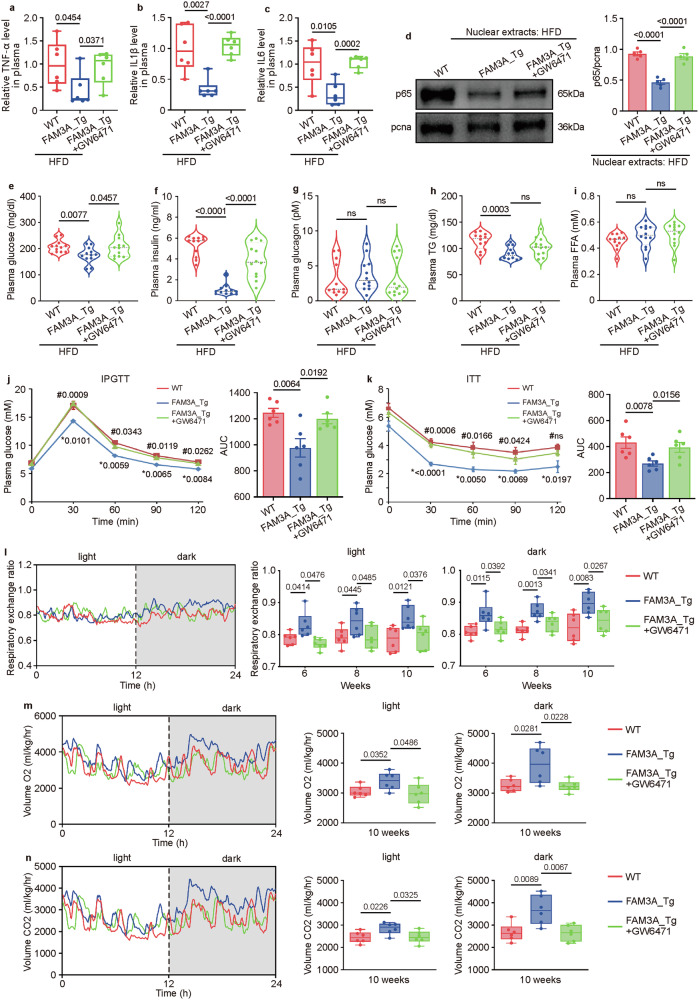
FAM3A-PPARα signaling affects metabolic and inflammatory characteristics. The TNF-α (**a**), IL1β (**b**), and IL6 (**c**) levels in soleus muscles were measured in mice treated with GW6471 and fed an HFD for 16 weeks and the results were plotted (n = 6 biologically independent animals/group). **d** Western blot images and quantification of the nuclear p65 levels in soleus muscles from the mice treated as described in a (n = 5 biologically independent samples/group; quantitative comparisons between samples were run on the same gel). The plasma fasting glucose (**e**), insulin (**f**), glucagon (**g**), triglyceride (TG, **h**), and free fatty acid (FFA, **i**) levels were measured and plotted for the mice treated as described in a (n = 12 biologically independent animals/group). Intraperitoneal glucose tolerance test (IPGTT, **j**) and insulin tolerance test (ITT, **k**) were performed on the mice treated as in a (n = 6 biologically independent animals/group). *FAM3A_Tg versus WT mice; ^#^FAM3A_Tg+GW6471 versus FAM3A_Tg mice. The respiratory exchange ratio (**l**) and the respiratory volumes of O_2_ (**m**) and CO_2_ (**n**) were measured and plotted for the mice fed an HFD for 6–10 weeks (n = 6 biologically independent animals/group). The data are presented as the means ± SEMs. Statistical significance was determined with a two-tailed independent *t* test, and *P* values are indicated (^ns^*P* ≥ 0.05). Source data are provided as a Source data file.

We further investigated whether the regulatory roles of FAM3A in inflammation and metabolic homeostasis were mediated by PPARα signaling. As shown, the anti-inflammatory effects of FAM3A were significantly repressed by GW6471 treatment both in vivo (Fig. 7a–d and Supplementary Fig. 8a–c) and in vitro (Supplementary Fig. 8d–f). Moreover, GW6471 treatment also abolished the FAM3A-induced decrease in plasma glucose (Fig. 7e and Supplementary Fig. 8g) and insulin (Fig. 7f and Supplementary Fig. 8h) levels. Furthermore, the improved glucose tolerance (Fig. 7j) and increased insulin sensitivity (Fig. 7k) were abolished by GW6471 treatment in FAM3A-transgenic mice. Additionally, the altered respiratory exchange ratio and consumption of O_2_ and CO_2_ in FAM3A-transgenic mice were reversed by GW6471 treatment during both the light period and the dark period (Fig. 7l–n and Supplementary Fig. 8l, m), suggesting the involvement of PPARα signaling in the regulatory effects of FAM3A on body glucose consumption.

Taken together, these findings suggest that FAM3A may promote lipid accumulation in muscle and ameliorate insulin resistance and inflammation by enhancing PPARα signaling.

### The actions of FAM3A/PPARα depend on the insulin receptor (insR)

**Fig. 8:**
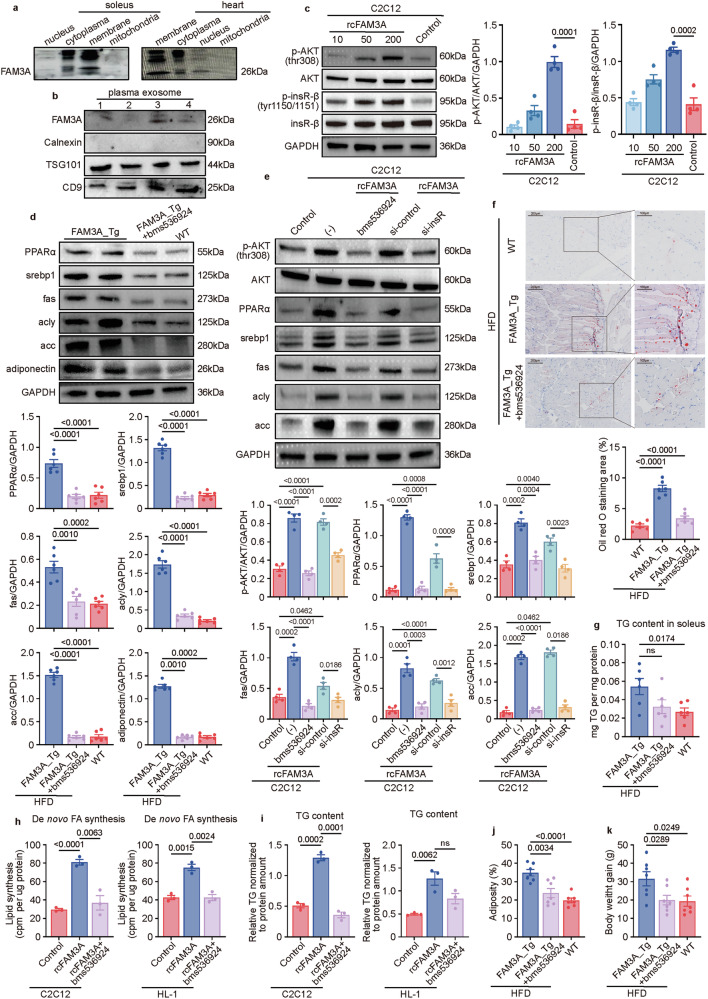
The effects of FAM3A on the regulation of muscle lipogenesis depend on the insulin receptor (insR). **a** Western blot images showing the presence of FAM3A in different cellular compartments in soleus muscles and heart tissues. **b** Levels of the FAM3A protein along with positive controls (TSG101 and CD9) and the negative control (calnexin) in plasma-derived exosomes from patients chosen at random. **c** Western blot images and quantification of the levels of p-AKT (thr308) and p-insR-β (tyr1150/1151) in cells treated with different concentrations (ng/ml) of rcFAM3A for 8 h (n = 4 biologically independent samples/group; quantitative comparisons between samples were run on the same gel). **d** Western blot images and quantification of the expression levels of the indicated proteins in soleus muscles from the mice treated with bms536924 and fed an HFD for 10 weeks (n = 6 biologically independent animals/group; quantitative comparisons between samples were run on the same gel). **e** Western blot images and quantification of the expression levels of the indicated proteins in cells pre-treated with bms536924 or transfected with the insR siRNA and then stimulated with rcFAM3A (200 ng/ml; n = 4 biologically independent samples/group; quantitative comparisons between samples were run on the same gel). The lipid droplets detected by oil red O staining (**f**) and TG content (**g**) were measured in soleus muscles from the mice treated with bms536924 and fed an HFD for five weeks and the results were plotted (n = 6 biologically independent animals/group). Scale bar: 200 μm, insets: 100 μm in (**f**). C2C12 cells were treated with rcFAM3A (200 ng/ml) for 12 hours. Cellular De novo FA synthesis (**h**) and TG content (**i**) were measured and graphed (n = 3 biologically independent samples/group). **j**, **k** Body adiposity and body weight gain were measured in mice treated with bms536924 and fed an HFD for 10 weeks and the results were plotted (n = 7 biologically independent animals/group). The data are presented as the means ± SEMs. Statistical significance was determined with a two-tailed independent *t* test, and *P* values are indicated (^ns^*P* ≥ 0.05). Source data are provided as a Source data file.

The cellular location of FAM3A is controversial. Many studies have reported that FAM3A is a mitochondrial protein, and few have suggested that it is a secreted peptide [9]. In our previous investigation, we reported that FAM3A was a cytokine [10]. In this study, we further assessed FAM3A expression in different cell components, and the results showed that FAM3A was present in the membrane vesicular transport system rather than in the mitochondria (Fig. 8a). Furthermore, both the human and mouse FAM3A amino acid sequences carry a signal peptide (Supplementary Fig. 9a). In addition, FAM3A could be packed in exosomes (Fig. 8b). We also found that FAM3A activated AKT signaling (Fig. 8c and Supplementary Fig. 9c). Since AKT activation is closely correlated with insR signaling pathway, we explored the involvement of insR in the actions of the FAM3A/PPARα axis. As expected, both in vitro and in vivo experiments revealed that FAM3A promoted the phosphorylation of insulin receptor β (insR-β) (Fig. 8c and Supplementary Fig. 9c). Moreover, the inhibition of insR by both chemical compound bms536924 and siRNA interference (Supplementary Fig. 9b) significantly abolished the activation of AKT induced by rcFAM3A in C2C12 cells (Fig. 8e). Furthermore, both in vivo and in vitro results showed that the inhibition of insR attenuated FAM3A-induced increase in muscular PPARα and lipogenic protein expression (Fig. 8d, e). Moreover, the increased muscular LD area (Fig. 8f) and triglyceride abundance (Fig. 8g) in FAM3A-transgenic mice were suppressed by bms536924. These effects of bms536924 were also assessed in vitro, and the FAM3A-induced increases in De novo FA synthesis process (Fig. 8h) and TG content (Fig. 8i) were repressed by treatment with bms536924 in C2C12 and HL-1 cells. Moreover, similar decreasing trends were observed in the assessment of the body adiposity rate (Fig. 8j) and body weight gain (Fig. 8k).

The anti-inflammatory effects of FAM3A were significantly repressed by bms536924 treatment both in vivo (Supplementary Fig. 9d–f) and in vitro (Supplementary Fig. 9g–i). Moreover, bms536924 treatment abolished the FAM3A-induced decreases in plasma glucose (Supplementary Fig. 9j) and insulin (Supplementary Fig. 9k) levels.

Taken together, these findings suggest that insR may be involved in the linkage between FAM3A and PPARα and their downstream actions.

## Discussion

In the present study, the functions of FAM3A in uncoupling lipid accumulation in muscles and insulin resistance (IR) were addressed. We found that (i) FAM3A increased lipogenesis and the lipid content in muscles; (ii) FAM3A increased adiponectin levels and a positive correlation was observed between FAM3A and adiponectin levels in patient plasma samples; (iii) FAM3A inhibited IR and metabolic disorders; and (iv) these functions of FAM3A were due to the induction of PPARα expression and depended on the insulin receptor (insR). In the following sections, these aspects are discussed individually.

How the body balances lipid accumulation and IR is interesting. Excess lipid content predisposes people to IR. White adipose tissue (WAT) has a limited ability to store excess calories as fat, and the excess lipids can also be stored in small lipid droplets (LDs) within nonadipose tissue such as skeletal muscle, heart, and liver. However, exceptional cases of the uncoupling of IR from obesity have been reported, for instance, the “athlete’s paradox”, namely, a higher lipid content is concomitant with increased insulin sensitivity. Therefore, the mechanism underlying the link between lipid accumulation and metabolic disorders is complex, and the exploration of effective treatments is feasible to avoid IR under the excess lipid accumulation conditions [19–21].

A number of transcription factors, including sterol regulatory element binding protein (srebp) isoforms, CCAAT-enhancer binding protein (C/EBP) isoforms, nuclear hormone receptors (NR1H2 and NR1H3), and peroxisome proliferator activated receptors (PPARs), have been shown to control the expression of lipogenic enzymes and genes in the lipogenic pathways [22, 23]. Srebp1 is a transcription factor that regulates the expression of lipogenic genes [24], and its downstream targets include srebp1 itself, fas, acc, and acly [25–28]. At present, the ability of FAM3A to regulate lipid metabolism has not been fully elucidated. Wang et al. reported that FAM3A could attenuate lipogenesis and fatty liver in diabetic mice [8]. Recently, FAM3A was shown to inhibit the expression of lipogenic genes and suppress lipid deposition in hepatocytes and liver tissues [29]. Previous studies have explored the effects of FAM3A in the liver. Our study of skeletal muscle indicated that FAM3A promoted lipogenesis in skeletal muscles. Specifically, increasing FAM3A levels both in vivo and in vitro increased the expression of srebp1 and lipogenic enzymes, including fas, acly, and acc (Fig. 2a, b and Supplementary Fig. 3a), the lipid content (Fig. 2c, d, f–i and Supplementary Fig. 3b–g), and body weight gain (Fig. 2j). Furthermore, in vitro experiments supported these results, as evidenced by the finding that FAM3A increased De novo FA synthesis in C2C12 cells (Fig. 2e). Perhaps, FAM3A regulates lipid biosynthesis in a tissue-specific manner. This hypothesis needs to be explored in depth.

Previous studies have indicated a role of FAM3A in IR and diabetes. Wang et al. reported that FAM3A attenuated hyperglycemia and IR in diabetic mice [8]. Moreover, FAM3A inhibited the expression of gluconeogenic genes and suppressed gluconeogenesis in the liver [29]. In accordance with these reports, FAM3A treatment decreased plasma glucose and insulin levels (Fig. 4d, e, i, j and Supplementary Fig. 4j, k) and promoted glucose consumption (Fig. 4k, l and Supplementary Fig. 4o, p). IR is accompanied by a chronic inflammation, and anti-inflammatory agents can be beneficial to alleviate IR [30–33]. As expected, FAM3A inhibited inflammation, as indicated by the decreased levels of TNF-α, IL1β, and IL6 in vitro and in vivo (Fig. 4a–c and Supplementary Fig. 4a–i). Therefore, anti-inflammatory effects might be responsible for the increased glucose consumption and alleviation of IR induced by FAM3A. In addition to these findings concerning inflammation and insulin resistance, the effects of FAM3A on mitochondrial homeostasis were explored in our study because of the significance of mitochondria in bioenergetic efficiency and metabolism [34]. Our study revealed that FAM3A treatment was an effective way to alleviate mitochondrial injury and promote mitochondrial function (Fig. 5). Concomitantly, blood perfusion and hemoglobin oxygen saturation increased (Supplementary Fig. 6a–c). Enhanced mitochondrial performance generally coordinates with an increased oxygen supply. We speculate that these effects may be due to angiogenesis induced by FAM3A, as a previous study revealed that FAM3A increased the capillary density and angiogenesis [18]. Taken together, the most important finding in our study is the novel role of FAM3A in uncoupling IR from increased lipid accumulation.

Adiponectin, which is produced and secreted predominantly by fat cells in adipose tissue, exerts pleiotropic effects on numerous tissues. Clinical studies have implicated adiponectin as a possible causative factor in the etiology of multiple diseases. An increase in circulating adiponectin levels can effectively offset any negative consequences of metabolic disorders including IR and obesity [35–37]. To date, few studies have explored the role of FAM3A in adipocyte differentiation. It has been indicated that FAM3A inhibited adipocyte differentiation [38]. However, the opposite conclusion was reached that FAM3A enhanced the adipogenesis of preadipocytes [39]. Our results revealed that both FAM3A transgene and rcFAM3A-injection in mice significantly upregulated adiponectin levels in skeletal muscle tissues (Fig. 3a, c) and WATs (Fig. 3b, d). Furthermore, these findings were confirmed by in vitro experiments in which rcFAM3A increased adiponectin expression in 3T3-L1 adipocytes (Fig. 3f). These results indicated that adiponectin levels were increased by FAM3A. Interestingly, a positive linear regression relationship was observed between the plasma levels of FAM3A and adiponectin in patients with or without metabolic disease (MD) (Fig. 3g). Importantly, plasma FAM3A levels were decreased along with metabolic disorders (Fig. 3g), consistent with previous studies reporting that FAM3A expression was downregulated in liver cells treated with high-lipid [40] and that chronic exposure to high concentrations of insulin repressed FAM3A expression in HepG2 cells [8]. Taken together, these results suggest that FAM3A is a novel cytokine that is downregulated in metabolic disorders and can positively regulate adiponectin levels.

As the first member of the PPAR family, PPARα is a transcription factor whose levels and activity are controlled by gene expression regulation and many different factors, including nutritional/hormonal cues, aging, cytokines, and growth factors [41, 42]. PPARα target genes are involved not only in glucose and lipid metabolism but also in inflammation-modulating pathways. PPARα can induce lipogenesis [12]. PPARα agonizts can increase the activity of srebp1c promoter and srebp1c protein levels [12, 43, 44]. Moreover, PPARα agonizts promoted lipogenesis in a srebp1c-dependent manner in the liver [45]. The loss of PPARα led to decreased expression levels of srebp1 targets, including FAS and ACC [46]. In our study, we identified FAM3A as a novel cytokine that controls PPARα levels, as shown by the results of in vivo and in vitro experiments (Fig. 6a–d). Most importantly, we found that lipogenesis elicited by FAM3A could be alleviated by a PPARα antagonist both in vivo and in vitro (Fig. 6e–l). Furthermore, the anti-inflammatory and anti-IR effects of FAM3A and the promotion of glucose consumption were significantly reversed by the inhibition of PPARα (Fig. 7). Therefore, our study identified FAM3A/PPARα axis as a novel signaling pathway that regulates metabolic disorders and balances excess lipids and IR.

The FAM3A cellular location is controversy. Currently, the majority of studies have proposed that FAM3A is a mitochondrial protein [40, 47, 48]. However, our previous study and another recent study have revealed that FAM3A is a secreted protein that lacks a mitochondrial localization signal [9, 10]. FAM3A colocolizes with cis-Golgi marker GM-130 and endoplasmic reticulum (ER) marker KDEL [9]. A signal peptide is present in the amino acid sequences of both the human and mouse FAM3A proteins (Supplementary Fig. 9a). Moreover, using muscle tissues, we detected that FAM3A was present at the cell membrane and in the cytoplasm rather than in the mitochondrial component (Fig. 8a). As a cytokine, it typically functions as a ligand to bind with a receptor. As presented in this study, FAM3A promoted glucose consumption, lipogenesis, and AKT activation (Fig. 8c and Supplementary Fig. 9c). In this sense, FAM3A functions similar to insulin. The lipogenic effect of SREBP1 can be assisted by insulin [49]. We therefore investigated whether insulin or insulin-like growth factor (IGF) receptors are involved in the actions of FAM3A. The pleiotropic effects of insulin are central in biology and medicine. Receptors of insulin and IGF are receptor tyrosine kinases whose signaling controls multiple aspects of animal physiology throughout life, including metabolism, growth, and even a variety of new, cell type-specific functions [50–52]. Interestingly, in addition to activate AKT, FAM3A promoted the phosphorylation of insR-β (Fig. 8c and Supplementary Fig. 9c). Using gene silencing or chemical inhibitor, we further found that the inhibition of insR reversed AKT activation and increased levels of PPARα, lipogenic transcription factor SREBP1, and lipogenic enzymes (Fig. 8d, e). Furthermore, the altered lipid synthesis and content and even body weight gain were abolished by insR blockade (Fig. 8f–k). InsR was also implicated in the anti-IR and anti-inflammatory effects of FAM3A (Supplementary Fig. 9d–k). These findings suggested that the effects of FAM3A depended on insR. However, an in-depth study is needed to decipher the direct or indirect association between FAM3A and insR.

In conclusion, we believe that the findings set forth in the current study should be of interest to lipidologists. However, the lack of knowledge of the FAM3A receptor limits the in-depth explorations of the mechanisms underlying the uncoupling of lipid accumulation in muscle from IR. The functions of FAM3A in regulating WATs and brown adipose tissues (BATs) are largely unknown and require further investigation. Is FAM3A another type of insulin-like growth factor? Does FAM3A explain the “athlete’s paradox”?

As a small peptide, FAM3A has potential clinical translational value. In the future, determining whether the increase in FAM3A levels in the body would be beneficial to the population with metabolic disorder, as delineated in the mouse models in our study, is worthy of investigation. We hope that targeting FAM3A will be beneficial for diagnosis and drug development to improve obesity-related IR in the future.

## Materials and methods

### Ethics statement

Our research complies with all relevant ethical regulations and our work with mice has been approved by the Ethics Review Board of Peking Union Medical College Hospital (Approval No. [XHDW-2023-083-2]; Beijing, China).

### Human specimen collection and clinical characteristics

A total of 48 patients with varicosity were included in this study (the basic clinical characteristics are provided in Supplementary Table 1); 22 patients with varicosity with metabolic disorder (MD), including diabetes, hyperlipidaemia, or obesity and 26 patients with varicosity without MD were included. Plasma samples were collected early in the morning and separated within 2 h from 5 mL peripheral blood samples by centrifugation at 2000 rpm for 10 min at 4 °C and stored at −80 °C until the detection of the levels of FAM3A (Lifespan, #LS-F35367) and adiponectin (Lifespan, #LS-F2599) using ELISA assays. In addition, the plasma samples were centrifuged at 1500 × *g* for 10 min to remove any cells and large particles, and then the supernatant was gently transferred to a new tube and centrifuged again at 10,000 × *g* for 10 min for exosome separation. The acquired exosomes were further filtered with qEVoriginal/70 nm (Izon) to obtain 1.5 ml fractions. Millipore Microcon-10 kDa units were used to centrifuge the fractions at 14,000 × *g* for 15 min at 4 °C for ultrafiltration and concentration, after which the fractions were stored at −80 °C until the detection of the amount of FAM3A by western blotting.

The use of human specimens was approved by the Ethics Review Board of Peking Union Medical College Hospital (Beijing, China). Written informed consent for the use of plasma samples was obtained from all patients included in the study.

### Generation of FAM3A-transgenic (FAM3A_Tg) mice

To generate systemic FAM3A-transgenic mice (NM_001379181.1), CAG-m-fam3a expression plasmid vector (Supplementary Fig. 1a) was constructed and microinjected into the zygotes of C57BL/6J mouse. The surviving zygotes were subsequently transferred into the pseudopregnant female ICR mouse. The first generation of transgenic pups (F0, founder mouse line) had a unique transgene insertion site, and the genotype was identified by PCR with the primers FAM3A-F (5’GCTGGTTGTTGTGCTGTCTCATC) and FAM3A-R (5’GCTGTCACGGAAGGCTAGATC) (Supplementary Fig. 1b). Each independent founder line was used to establish germline-transmitted mice by breeding one generation (F1) transgenic mice.

### Experimental animals and treatment

In this study, male C57BL/6J mice (18–25 g) were selected based on inclusion criteria of health and specific physiological characteristics. The exclusion criteria included significant diseases, abnormal body weight, and mortality during the experiment. All the criteria were pre-established to ensure data accuracy and consistency. C57BL/6J mice (Vital River, China) or FAM3A-transgenic mice were housed in a temperature- and humidity-controlled environment and had free access to a standard laboratory diet (Vital River, China) and water. In some experiments, 6- to 8-week-old mice were fed a synthetic high-fat diet (HFD) containing 45% energy from lard (Researchdiet, Canada, #D12451) for 10 weeks. To infuse of FAM3A in vivo, the rcFAM3A protein (NM_021806; Origene, #TP303495) was slowly dissolved at 4 °C prior to use, at a concentration of 1 µg/mL in a sterilized PBS solution. The recombinant protein solution was injected via the tail vein in a volume of 200 µl (containing 0.2 µg of FAM3A protein) each time once every 7 days until the HFD-fed model was established. For PPARα inhibition, the mice were treated with 15 mg/kg/day GW6471 (Topscience, #T8486) by oral gavage. For insulin receptor (insR) inhibition, the mice were treated with 20 mg/kg/day bms536924 (Topscience, #T6419) by oral gavage. Specifically, the inhibitors were initially dissolved in DMSO and then in vehicle containing 10% 1-methyl-2-pyrrolidone and 5% Cremophor EL. The mice were assigned to experimental groups in an alternating and balanced manner to avoid cage, batch, and handling bias and no formal randomization method was performed. Blinding was not applied during treatment administration or sample collection.

For the analysis of the plasma metabolic profile, the mice were fasted for 8 h and blood samples were collected for analysis of the levels of triglyceride (TG) (Abcam, #ab65336), free fatty acid (FA) (Abcam, #ab65336), and glucose (Applygen, China, #E1010) using a colorimetric method and levels of insulin (CrystalChem, #90080) and glucagon (Mercodia, #10-1281-01) using ELISAs. Adiposity was measured by an index analyser (Bruker, #MinispecLF50) to quantify the percentage of body fat. For some experiments, the gonadal white adipose tissue (gWAT) surrounding the testes (male mice) and ovaries (female mice) and the inguinal white adipose tissue (iWAT), located beneath the skin in the groin region, were precisely excised and weighed. To determine microcirculation profiles in hind limbs of mice, an Oxygento See (O2C; LEA Medizintechnik GmbH, Giessen, Germany) and a dual-channel laser Doppler monitoring instrument (VMS-LDF2; Moor Instrument, Ltd, Axminster, United Kingdom) were used to configure the multimodal device to acquire microcirculatory oxygen and hemodynamic data from the hind limbs of the mice, including hemoglobin oxygen saturation (SO_2_), deoxyhaemoglobin (deoxyHb), and oxyhaemoglobin (oxyHb). The microcirculatory blood perfusion in the hind limb was continuously scanned for 1 min using laser speckle contrast imaging (LSCI; RFLSI III, RWD Life Sciences). For the analysis of mouse adiposity, WAT weight, and microcirculation profiles in the hind limb, the investigators were blinded to the group allocations during the measurements and data analysis, and the mice were tested in a randomized order.

For all the animal experiments, the sample size was chosen based on the literature and the variability observed in previous experience in our laboratory.

### Histological immunofluorescence

The soleus tissue was fixed with 4% paraformaldehyde and then dehydrated and embedded in paraffin. The paraffin-embedded tissue sections were pre-stained and incubated at room temperature with 5% normal goat serum for 20 min. After the sealed serum incubation, the tissue sections were further incubated with the primary antibody anti-FAM3A (Origene, #TA324017, 1:100), overnight at 4 °C in a humid chamber. Afterwards, the secondary antibody was incubated with the sections at room temperature in the dark for 60 min to bind the fluorophore. After the slices were slightly dried, DAPI staining solution was added and the samples were incubated at room temperature in the dark for 10 min. The fluorescence-stained sections were then visualized using a panoramic scanning system (Pannoramic MIDI, 3D HISTECH). The FAM3A signaling was quantified with ImageJ (ver. 1.53) and the value was quantified as the ratio of FMA3A area to DAPI area in a visual field.

### Immunohistochemistry

The soleus tissues and the gWATs that surround the testes were fixed with 10% formalin immediately after surgical resection and embedded in paraffin. The tissues were cut into 5 μm sections which were subsequently incubated with 3% hydrogen peroxide to block endogenous peroxidase activity. The tissue sections were blocked with 10% BSA for 1 h and incubated with primary antibodies against FAM3A (Origene, #TA324017; 1:100), adiponectin (Abcam, #ab22554; 1:200), and PPARα (Invitrogen, #PA1-822A; 1:100), at 4 °C overnight. Then, the HRP-conjugated secondary antibody was added to the sections and incubated. DAB substrate was added, and the sections were examined with a light microscope. The positively stained area was quantified in a visual field at ×40 magnification with ImageJ (ver. 1.53), and the values are presented as a percentage of the total area in a visual field.

### Haematoxylin-eosin (HE) and oil red O staining

HE and oil red O staining of tissue sections were routinely performed. Briefly, skeletal muscle tissues and gWATs were cut into 5 μm sections on a microtome. The tissue sections were deparaffinated by immersion in xylene and rehydration, and then stained with HE or an oil red O kit (Sigma, #102419) and examined with a light microscope. For the oil red O staining, the positively stained area was quantified in a visual field at ×40 magnification with ImageJ (ver. 1.53). The values are presented as a percentage of the total area in a visual field. For the measurement of adipocyte diameter, the areas of adipocytes were precisely measured in a visual field at ×40 magnification and the diameters were calculated based on the measured area and the quantified cell number with ImageJ (ver. 1.53).

### Cell culture and treatment

Commercially acquired human cardiac MVECs (hcMVECs, Sciencell, USA; #6000) were cultured in ECM (Sciencell, USA; #1001) supplemented with ECGS (Sciencell, USA; #1052), 5% FBS (Sciencell, USA; #0025), and P/S solution (Sciencell, USA; #0503) under standard conditions (5% CO_2_/pH 7.2–4/37 °C/95% humidity). For lipid or glucose treatments, cells at passage No.2–3 were grown until confluence.

Commercially acquired C2C12 mouse myoblast cell lines (Fenghui, China; #CL0058), HL-1 mouse cardiac myocytes (Cellverse, China; #icell-m077), and 3T3-L1 adipocytes derived from mouse embryonic fibroblasts (Cellverse, China; #icell-m066) were cultured in DMEM supplemented with 10% FBS (Sciencell, USA; #0025), and P/S solution. Specifically, mature 3T3-L1 adipocytes were used between 8 and 10 days after the start of differentiation. These commercially purchased C2C12, HL-1, and 3T3-L1 cell lines were authenticated through STR profiling. They were also tested for mycoplasma contamination and found to be free of contamination.

For the experiments, the C2C12, HL-1, and 3T3-L1 cell lines were treated with 200 ng/ml rcFAM3A or chemical PPARα inhibitor GW6471 (8 μM diluted with DMSO; Topscience, #T8486) [53] or chemical insulin receptor (insR) inhibitor bms536924 (4 μΜ diluted with DMSO; Topscience, #T6419) for 12 h. For insR silencing, C2C12 cells were incubated with 0.5 μg/ml siRNA (Santa Cruz, #sc-35673). After 48 h, cells were treated with rcFAM3A.

### Respiratory exchange ratio (RER) measurement

Mice were housed individually in sealed metabolic cages (Oxymax, Columbus Instruments, CLAMS-8). Before data collection (3 cycles of 12 h of light/12 h of dark), mice were acclimated to the metabolic cages for 6–8 h. RER data were compiled with Oxymax software and analyzed with Microsoft Excel by averaging readings taken every 20 min during each light/dark cycle. During the measurements and data analysis, the mice were tested in a randomized order and the investigators were blinded to the group allocations. All the data were adjusted for body weight.

### Real-time PCR

Total RNA was extracted from tissues or cells with TRIzol reagent (Sigma-Aldrich, USA) and transcribed into cDNA using a PrimeScript™ RT Reagent Kit (Takara, Japan) according to the manufacturer’s instructions. The reaction mixture was used as the template to perform real-time PCR. The primers used are listed in Supplementary Table 2.

### Western blot analysis

Anti-FAM3A (Origene, #TA324017; 1:1000), anti-adiponectin (Cell Signaling Technology, #2789; 1:1000), anti-PPARα (Santa Cruz, #sc-398394; 1:500), anti-srebp1 (Santa Cruz, #sc-13551; 1:500), anti-fas (Cell Signaling Technology, #3180; 1:1000), anti-acly (Cell Signaling Technology, #4332; 1:1000), anti-acc (Cell Signaling Technology, #3676; 1:1000), anti-p65 (Santa Cruz, #sc-8008; 1:500), anti-pcna (Santa Cruz, #sc-25280; 1:500), anti-p-AKT (thr308) (Cell Signaling Technology, #9275; 1:1000), anti-AKT (Cell Signaling Technology, #9272; 1:1000), anti-p-insR-β (tyr1150/1151) (Cell Signaling Technology, #3024; 1:1000), anti-insR-β (Cell Signaling Technology, #3025; 1:1000), anti-Calnexin (Abcam, #ab75801; 1:1000), anti-TSG101 (Abcam, #ab125011; 1:1000), anti-CD9 (Abcam, #ab92726; 1:1000), anti-β-actin (LABLEAD, #A0101; 1:4000), anti-GAPDH (Cell Signaling Technology, #5174; 1:4000), and HRP-conjugated secondary antibodies were commercially obtained (Supplementary Table 6). Western blot was performed according to previous protocols wildly described. For the detection of nuclear localization of a protein, the tissue nuclear extracts were obtained using a commercial nuclear extract kit (Beyotime, #P0027) according to the manufacturer’s instructions. To quantify protein signals, we subtracted background and normalized the value to β-actin or GAPDH or PCNA. To determine protein phosphorylation signal, we quantified the relative value to its total signal and then normalized this value to that of β-actin or GAPDH. Full and uncropped western blots are provided in the Supplementary Material.

### De novo lipogenesis assay

C2C12 or HL-1 cells cultured in 12-well plates were treated with rcFam3A (200 ng/ml) for 12 h, with or without pre-treatment with PPARα inhibitor GW6471 (8 μM) or insulin receptor inhibitor bms536924 (4 μM) for 30 min, and then [1-^14^C]-acetic acid (Perkin Elmer, #NEC084H001MC) was spiked into the medium at a final radioactivity of 4 μCi/mL. After an incubation for 4 h at 37 °C, the cells were washed 3 times with ice-cold PBS and scraped into 200 μl of cold PBS supplemented with 0.5% TritonX-100 and protease inhibitors. Then, 120 μl of crude lysate was mixed with 400 μl of a 2:1 chloroform/methanol mixture and vortexed. Following addition of 100 μl of water, the samples were vortexed and centrifuged for 15 min at 1000 × *g* at room temperature. The 200 μl of the lower/organic phase was mixed with Emulsifier Safe scintillation cocktail, and ^14^C was quantified using a Hidex 300 SL counter. The remaining crude lysate was clarified for 10 min at maximum speed, and the protein in the supernatant was quantified to normalize the amount of ^14^C incorporated into the organic phase.

### Triglyceride (TG) assay

TG content in cells or tissues was determined using a Triglyceride Colorimetric Assay (Cayman Chemical, #10010303) according to the manufacturer’s instructions. Briefly, C2C12 or HL-1 cells were washed twice with ice-cold PBS, and then collected and suspended in 30 μl of 1× NP40 substitute assay reagent. The 5 μl of this suspension was diluted in NP40 lysis buffer, and the supernatant was used to quantify the protein amount. The remaining sample was heated to 80 °C, then vortexed, and heated again. Samples were centrifuged for 8 min at 10,000 × *g* at room temperature. TG concentration in the supernatant was tested with a colorimetric assay. To quantify the TG content in each sample, the value was normalized to the protein amount in that sample. Additionally, to determine TG content in a tissue, the soleus muscle was homogenized in 200 μl water in 1.5 ml NAVY bead lysis kit tubes using a tissue homogenizer. The sample was further mixed with 50 μl of 5 × NP40 substitute assay reagent, and then TG and protein amount were assayed and quantified.

### Cellular lipid droplet (LD) quantification

Cells were plated in glass-bottom dishes (Mattek LifeScience, #P35G-1.5-14-C) and labeled with Bodipy 493/503 (1 μM, Thermo, #D3922) and Hoechst 33342 (1 μM, Thermo, #H3570) for 15 min at 37 °C, and subsequently washed. The LD fluorescence intensity in each cell was quantified using ImageJ (ver. 1.53).

### Seahorse mitochondrial flux analyses

The mitochondrial oxygen consumption rate (OCR) was measured with an XFpro extracellular Analyzer (Seahorse Bioscience, USA) according to the manufacturer’s instructions. Briefly, C2C12 cells were seeded in special Seahorse plates. One hour before measurements were taken, glucose (10 mM) or pyruvate (1 mM) was supplemented into XF medium in a CO_2_-free incubator at 37 °C. The basal cellular OCR was recorded in the absence of any inhibitors or uncouplers. After baseline measurement, oligomycin (ATP synthase inhibitor; 1 μM) was added to measure the OCR for ATP production. To determine maximal respiration, the uncoupler carbonyl cyanide 4-(trifluoromethoxy) phenylhydrazone (FCCP; 0.5 μM) was added. To determine the non-mitochondrial OCR, Rotenone (1 μM; complex I inhibitor) and antimycin A (1 μM; complex III inhibitor) were used. To quantify the mitochondrial OCR, the non-mitochondrial OCR was subtracted from the basal OCR. A typical OCR trace from this mitochondrial flux assay and how each parameter was calculated are shown in Fig. 5c. After the assays were performed, the plates were saved and the protein concentration in each well was determined using bicinchoninic acid (BCA) protein assay. In each plate, the same treatment was performed in triplicate, and three independent experiments were performed for each group.

### Measurement of ATP levels

ATP concentrations were measured with an ATP bioluminescence assay kit CLS II (Roche, #11699695001). Briefly, cells or tissues were lysed with a lysis buffer. Reaction was initiated by dispensing Luciferase reagent into all the samples. Luminescence was measured with a microplate reader. Additionally, protein concentrations were measured with a BCA protein assay. The ATP level in each sample was represented as nmol/mg protein. Each sample was analyzed in triplicate and at least three independent experiments were performed for each group.

### Determination of mitochondrial membrane potential

The mitochondrial membrane potential (Δψm) was determined using tetramethylrhodamine ethyl ester (TMRE, MedChemExpress, #115532-52-0). Briefly, cells were seeded in black 96-well plates and cultured. TMRE (1 μM) was added and incubated for 20 min at 37 °C. Cells were then washed with 0.2% BSA in PBS. The fluorescence was detected at 575 nm using a Synergy HTX multimode Reader (BioTek) and the detected fluorescence signaling was corrected to the protein amount in each sample. Additionally, FCCP (1 μM) was used for negative control to eliminate mitochondrial membrane potential. All the values were normalized to the control, which was arbitrarily defined as one fold. Each sample was measured in triplicate, and at least three independent experiments were performed for each group.

### Measurements of mitochondrial respiratory chain enzyme activity

Measurements of complex I activity (Complex I enzyme activity microplate assay kit; Abcam, #ab109721), complex II activity (Complex II enzyme activity microplate assay kit; Abcam, #ab109908), and complex IV activity (Complex IV enzyme activity microplate assay kit; Abcam, #ab109911) in C2C12 cells were performed according to the manufacturer’s instructions. Briefly, C2C12 cells were pre-treated with GW6471 for 30 min and then treated with rcFAM3A for 8 h. The cells were harvested and loaded onto the plate for 3 h. Afterwards, 200 μl of assay solution was added and optical density (OD) values were measured in kinetic mode. The OD value of each sample is presented as the absorbance per minute per 100 μg of cell lysate.

### Citrate synthase activity assays

Whole-cell citrate synthase activity was measured using a Citrate synthase activity assay kit (Abcam, #ab239712). Briefly, 1 × 10^6^ cells were incubated with ice cold citrate synthase assay buffer, and then centrifuged. The supernatant was collected and mixed with reaction buffer, and then absorbance was measured immediately at 412 nm with a spectrophotometer in kinetic mode at 25 °C for 20 min. Each value was normalized to the protein content in each sample, and the citrate synthase activity was represented as nmol per min per mg protein.

### Determination of mtDNA levels

Total DNA, including mitochondrial DNA (mtDNA) and nuclear DNA (nDNA) was extracted from cells using a DNeasy Blood & Tissue Kit (Qiagen, #69504). The contents of mtDNA and nDNA were determined by quantifying relative gene expression level (cytochrome b for mtDNA and Ppia for nDNA) with quantitative real-time PCR. The mtDNA gene expression value was corrected by that of nDNA gene expression, and the calculated values were normalized to those of the control, which was arbitrarily defined as one fold.

### Statistics

All the values are presented as the means ± SEMs. The normality and equal variance of all values were assessed using the Kolmogorov-Smirnov test to determine the choice between parametric or nonparametric tests. When the values conformed to normality and equal variance, to determine the differences between groups, unpaired *t* test was used for comparisons between 2 groups, and one-way analysis of variance (ANOVA) was used for comparisons between > 2 groups, followed by the Bonferroni post hoc test. Otherwise, the Mann-Whitney nonparametric test for two independent samples was used to define differences between groups. In each case, significance was defined as *P* < 0.05. Statistical analyses were performed with IBM SPSS Statistics 25 software. In this study, the sample size was chosen empirically based on the literature and the experimental variability observed in our previous study.

## Supplementary information

Supplementary materials

Uncropped blots

Dataset1

## Data Availability

The raw mass spectrometry proteomic data are deposited in the iProX database (accession number: IPX0012785000). This paper does not report the original code. Supplementary tables and figures are available in the Supplemental Material. Unprocessed blots are available as a PDF file “Uncropped blots” in the Supplementary Material. Correspondence and reasonable requests for materials should be addressed to DY or YZ.

## References

[1] Zhu Y, Xu G, Patel A, McLaughlin MM, Silverman C, Knecht KA, et al. Cloning, expression, and initial characterization of a novel cytokine-like gene family. Genomics. 2002;80:144–50. 12160727 10.1006/geno.2002.6816

[2] Zhang X, Yang W, Wang J, Meng Y, Guan Y, Yang J. FAM3 gene family: a promising therapeutical target for NAFLD and type 2 diabetes. Metabolism. 2018;81:71–82. 29221790 10.1016/j.metabol.2017.12.001

[3] Yang J, Guan Y. Family with sequence similarity 3 gene family and nonalcoholic fatty liver disease. J Gastroenterol Hepatol. 2013;28:105–11. 23855304 10.1111/jgh.12033

[4] Wang C, Burkhardt BR, Guan Y, Yang J. Role of pancreatic-derived factor in type 2 diabetes: evidence from pancreatic beta cells and liver. Nutr Rev. 2012;70:100–6. 22300596 10.1111/j.1753-4887.2011.00457.x

[5] Li X, Yuan F, Xiong Y, Tang Y, Li Z, Ai J, et al. FAM3A plays a key role in protecting against tubular cell pyroptosis and acute kidney injury. Redox Biol. 2024;74:103225. 38875957 10.1016/j.redox.2024.103225 PMC11226986

[6] Yan H, Meng Y, Li X, Xiang R, Hou S, Wang J, et al. FAM3A maintains metabolic homeostasis by interacting with F1-ATP synthase to regulate the activity and assembly of ATP synthase. Metabolism. 2023;139:155372. 36470472 10.1016/j.metabol.2022.155372

[7] Wang D, Wei T, Cui X, Xia L, Jiang Y, Yin D, et al. Fam3a-mediated prohormone convertase switch in alpha-cells regulates pancreatic GLP-1 production in an Nr4a2-Foxa2-dependent manner. Metabolism. 2025;162, 156042. 10.1016/j.metabol.2024.156042 39362520

[8] Wang C, Chi Y, Li J, Miao Y, Li S, Su W, et al. FAM3A activates PI3K p110alpha/Akt signaling to ameliorate hepatic gluconeogenesis and lipogenesis. Hepatology. 2014;59:1779–90. 24806753 10.1002/hep.26945

[9] Sala D, Cunningham TJ, Stec MJ, Etxaniz U, Nicoletti C, Dall’Agnese A, et al. The Stat3-Fam3a axis promotes muscle stem cell myogenic lineage progression by inducing mitochondrial respiration. Nat Commun. 2019;10:1796. 30996264 10.1038/s41467-019-09746-1 PMC6470137

[10] Lei C, Kan H, Xian X, Chen W, Xiang W, Song X, et al. FAM3A reshapes VSMC fate specification in abdominal aortic aneurysm by regulating KLF4 ubiquitination. Nat Commun. 2023;14:5360. 37660071 10.1038/s41467-023-41177-x PMC10475135

[11] Szukiewicz D. Molecular mechanisms for the vicious cycle between insulin resistance and the inflammatory response in obesity. Int J Mol Sci. 2023;24:9818. 37372966 10.3390/ijms24129818 PMC10298329

[12] Bougarne N, Weyers B, Desmet SJ, Deckers J, Ray DW, Staels B, et al. Molecular actions of PPARalpha in lipid metabolism and inflammation. Endocrine Rev. 2018;39:760–802. 30020428 10.1210/er.2018-00064

[13] Staels B. PPAR agonists and the metabolic syndrome. Therapie. 2007;62:319–26. 17983557 10.2515/therapie:2007051

[14] Lefebvre P. Sorting out the roles of PPAR alpha in energy metabolism and vascular homeostasis. J Clin Investig. 2006;116:571–80. 16511589 10.1172/JCI27989 PMC1386122

[15] Hagberg CE, Falkevall A, Wang X, Larsson E, Huusko J, Nilsson I, et al. Vascular endothelial growth factor B controls endothelial fatty acid uptake. Nature. 2010;464:917–21. 20228789 10.1038/nature08945

[16] Savage DB, Petersen KF, Shulman GI. Disordered lipid metabolism and the pathogenesis of insulin resistance. Physiol Rev. 2007;87:507–20. 17429039 10.1152/physrev.00024.2006 PMC2995548

[17] Petersen KF, Shulman GI. Cellular mechanism of insulin resistance in skeletal muscle. Journal R Soc Med. 2002;95:8–13. 12216329 PMC1308947

[18] Xu W, Liang M, Zhang Y, Huang K, Wang C. Endothelial FAM3A positively regulates post-ischaemic angiogenesis. EBioMedicine. 2019;43:32–42. 31000420 10.1016/j.ebiom.2019.03.038 PMC6562148

[19] Chen J, Markworth JF, Ferreira C, Zhang C, Kuang S. Lipid droplets as cell fate determinants in skeletal muscle. Trends Endocrinol Metab TEM. 2025;36:645–59. 39613547 10.1016/j.tem.2024.10.006 PMC12116854

[20] Krahmer N, Farese RV Jr., Walther TC. Balancing the fat: lipid droplets and human disease. EMBO Mol Med. 2013;5:973–83. 23740690 10.1002/emmm.201100671 PMC3721468

[21] Verboven K, Wouters K, Gaens K, Hansen D, Bijnen M, Wetzels S, et al. Abdominal subcutaneous and visceral adipocyte size, lipolysis and inflammation relate to insulin resistance in male obese humans. Sci Rep. 2018;8:4677. 29549282 10.1038/s41598-018-22962-x PMC5856747

[22] Piché ME, Tchernof A, Després JP. Obesity phenotypes, diabetes, and cardiovascular diseases. Circ Res. 2020;126:1477–1500. 32437302 10.1161/CIRCRESAHA.120.316101

[23] Seravalle G, Grassi G. Obesity and hypertension. Pharmacol Res. 2017;122:1–7. 28532816 10.1016/j.phrs.2017.05.013

[24] Marques-Oliveira GH, Silva TM, Lima WG, Valadares HMS, Chaves VE. Insulin as a hormone regulator of the synthesis and release of leptin by white adipose tissue. Peptides. 2018;106:49–58. 29953915 10.1016/j.peptides.2018.06.007

[25] Leonard AE, Pereira SL, Sprecher H, Huang YS. Elongation of long-chain fatty acids. Prog Lipid Res. 2004;43:36–54. 14636670 10.1016/s0163-7827(03)00040-7

[26] Kim YM, Shin HT, Seo YH, Byun HO, Yoon SH, Lee IK, et al. Sterol regulatory element-binding protein (SREBP)-1-mediated lipogenesis is involved in cell senescence. J Biol Chem. 2010;285:29069–77. 20615871 10.1074/jbc.M110.120386 PMC2937938

[27] Shimano H, Horton JD, Hammer RE, Shimomura I, Brown MS, Goldstein JL. Overproduction of cholesterol and fatty acids causes massive liver enlargement in transgenic mice expressing truncated SREBP-1a. J Clin Investig. 1996;98:1575–84. 8833906 10.1172/JCI118951 PMC507590

[28] Angela M, Endo Y, Asou HK, Yamamoto T, Tumes DJ, Tokuyama H, et al. Fatty acid metabolic reprogramming via mTOR-mediated inductions of PPARgamma directs early activation of T cells. Nat Commun. 2016;7:13683. 27901044 10.1038/ncomms13683 PMC5141517

[29] Hu CQ, Hou T, Xiang R, Li X, Li J, Wang TT, et al. PANX1-mediated ATP release confers FAM3A’s suppression effects on hepatic gluconeogenesis and lipogenesis. Mil Med Res. 2024;11:41. 38937853 10.1186/s40779-024-00543-6 PMC11210080

[30] Lee SH, Park SY, Choi CS. Insulin resistance: from mechanisms to therapeutic strategies. Diabetes Metab J. 2022;46:15–37. 34965646 10.4093/dmj.2021.0280 PMC8831809

[31] Hill MA, Yang Y, Zhang L, Sun Z, Jia G, Parrish AR, et al. Insulin resistance, cardiovascular stiffening and cardiovascular disease. Metab Clin Exp. 2021;119:154766. 33766485 10.1016/j.metabol.2021.154766

[32] Yazici D, Sezer H. Insulin resistance, obesity and lipotoxicity. Adv Exp Med Biol. 2017;960:277–304. 10.1007/978-3-319-48382-5_12 28585204

[33] Perry RJ, Samuel VT, Petersen KF, Shulman GI. The role of hepatic lipids in hepatic insulin resistance and type 2 diabetes. Nature. 2014;510:84–91. 24899308 10.1038/nature13478 PMC4489847

[34] Liesa M, Shirihai OS. Mitochondrial dynamics in the regulation of nutrient utilization and energy expenditure. Cell Metab. 2013;17:491–506. 23562075 10.1016/j.cmet.2013.03.002 PMC5967396

[35] Straub LG, Scherer PE. Metabolic Messengers: Adiponectin. Nature Metab. 2019;1:334–9. 32661510 10.1038/s42255-019-0041-z PMC7357716

[36] Engin A. Adiponectin resistance in obesity: adiponectin leptin/insulin interaction. Adv Exp Med Biol. 2024;1460:431–62. 10.1007/978-3-031-63657-8_15 39287861

[37] Tam E, Ouimet M, Sweeney G. Cardioprotective effects of adiponectin-stimulated autophagy. J Lipid Atheroscler. 2025;14:40–53. 39911962 10.12997/jla.2025.14.1.40 PMC11791421

[38] Kang T, Peng D, Bu G, Gu H, Zhang F, Zhang R, et al. Transcriptional regulation analysis of FAM3A gene and its effect on adipocyte differentiation. Gene. 2016;595:92–98. 27688071 10.1016/j.gene.2016.09.038

[39] Chi Y, Li J, Li N, Chen Z, Ma L, Peng W, et al. FAM3A enhances adipogenesis of 3T3-L1 preadipocytes via activation of ATP-P2 receptor-Akt signaling pathway. Oncotarget. 2017;8:45862–73. 28515350 10.18632/oncotarget.17578 PMC5542233

[40] Zhou Y, Jia S, Wang C, Chen Z, Chi Y, Li J, et al. FAM3A is a target gene of peroxisome proliferator-activated receptor gamma. Biochim Biophys Acta. 2013;1830:4160–70. 23562554 10.1016/j.bbagen.2013.03.029

[41] Ezhilarasan D. Deciphering the molecular pathways of saroglitazar: A dual PPAR alpha/gamma agonist for managing metabolic NAFLD. Metab Clin Exp. 2024;155:155912. 38609038 10.1016/j.metabol.2024.155912

[42] Tahri-Joutey M, Andreoletti P, Surapureddi S, Nasser B, Cherkaoui-Malki M, Latruffe N. Mechanisms mediating the regulation of peroxisomal fatty acid beta-oxidation by PPARalpha. Int J Mol Sci. 2021;22:8969. 34445672 10.3390/ijms22168969 PMC8396561

[43] Fernández-Alvarez A, Alvarez MS, Gonzalez R, Cucarella C, Muntané J, Casado M. Human SREBP1c expression in liver is directly regulated by peroxisome proliferator-activated receptor alpha (PPARalpha). J Biol Chem. 2011;286:21466–77. 21540177 10.1074/jbc.M110.209973 PMC3122206

[44] Koo SH, Satoh H, Herzig S, Lee CH, Hedrick S, Kulkarni R, et al. PGC-1 promotes insulin resistance in liver through PPAR-alpha-dependent induction of TRB-3. Nat Med. 2004;10:530–4. 15107844 10.1038/nm1044

[45] Oosterveer MH, Grefhorst A, van Dijk TH, Havinga R, Staels B, Kuipers F, et al. Fenofibrate simultaneously induces hepatic fatty acid oxidation, synthesis, and elongation in mice. J Biol Chem. 2009;284:34036–44. 19801551 10.1074/jbc.M109.051052 PMC2797174

[46] Patel DD, Knight BL, Wiggins D, Humphreys SM, Gibbons GF. Disturbances in the normal regulation of SREBP-sensitive genes in PPAR alpha-deficient mice. J Lipid Res. 2001;42:328–37. 11254743

[47] Jia S, Chen Z, Li J, Chi Y, Wang J, Li S, et al. FAM3A promotes vascular smooth muscle cell proliferation and migration and exacerbates neointima formation in rat artery after balloon injury. J Mol Cell Cardiol. 2014;74:173–82. 24857820 10.1016/j.yjmcc.2014.05.011

[48] Xiang R, Chen J, Li S, Yan H, Meng Y, Cai J, et al. VSMC-specific deletion of FAM3A attenuated Ang II-promoted hypertension and cardiovascular hypertrophy. Circ Res. 2020;126:1746–59. 32279581 10.1161/CIRCRESAHA.119.315558

[49] Saltiel AR. Insulin signaling in health and disease. J Clin Investig. 2021;131:e142241. 10.1172/JCI142241 PMC7773347 33393497

[50] Haeusler RA, McGraw TE, Accili D. Biochemical and cellular properties of insulin receptor signalling. Nat Rev Mol Cell Biol. 2018;19:31–44. 28974775 10.1038/nrm.2017.89 PMC5894887

[51] Choi E, Duan C, Bai X. Regulation and function of insulin and insulin-like growth factor receptor signalling. Nat Rev Mol Cell Biol. 2025;26:558–80. 39930003 10.1038/s41580-025-00826-3 PMC12631569

[52] Kadowaki T, Ueki K, Yamauchi T, Kubota N. SnapShot: insulin signaling pathways. Cell. 2012;148:624–624.e1 e621. 22304926 10.1016/j.cell.2012.01.034

[53] Castelli V, Catanesi M, Alfonsetti M, Laezza C, Lombardi F, Cinque B, et al. PPARalpha-selective antagonist GW6471 inhibits cell growth in breast cancer stem cells inducing energy imbalance and metabolic stress. Biomedicines. 2021;9:127. 33525605 10.3390/biomedicines9020127 PMC7912302

