# Supplementary material for: FAM3A drives uncoupling of muscle lipid accumulation and insulin resistance depending on insulin receptor
Source: Cell Death Dis. 2025 Dec 7;17(1):72. doi: 10.1038/s41419-025-08298-1 (PMC12827484; doi:10.1038/s41419-025-08298-1)
Supplement: Supplementary file 1 — Supplementary materials [file 41419_2025_8298_MOESM1_ESM.pdf]

Supplementary materials for

FAM3A drives uncoupling of muscle lipid accumulation and insulin resistance depending on insulin receptor

Dan Yang *et al*.

Correspondence to:
zhengyuehong2022@outlook.com

This PDF file includes:

- Supplementary Figs. 1 to 9;
- Supplementary Tables 1 to 7 (animals, cells, and reagent major resources are included in Tables 4-7).

Supplementary Figures

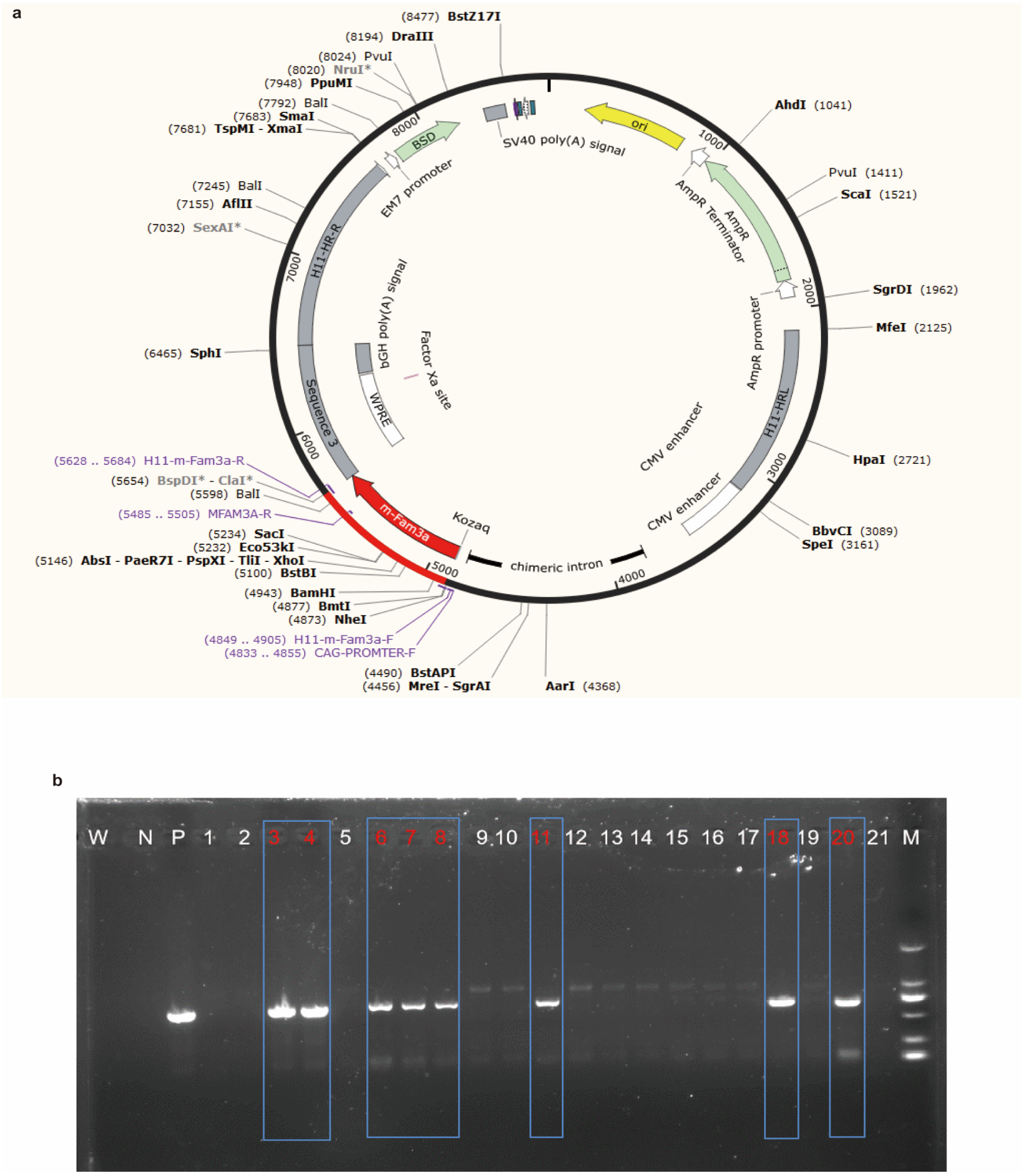

Supplementary Fig. 1 Construction and identification of FAM3A-transgenic (FAM3A_Tg) mice. a Genetic information of CAG-mfam3a vector. **b** Identification of F0 generation by PCR shows eight F0 generation mice with FAM3A transgene (blue frame).

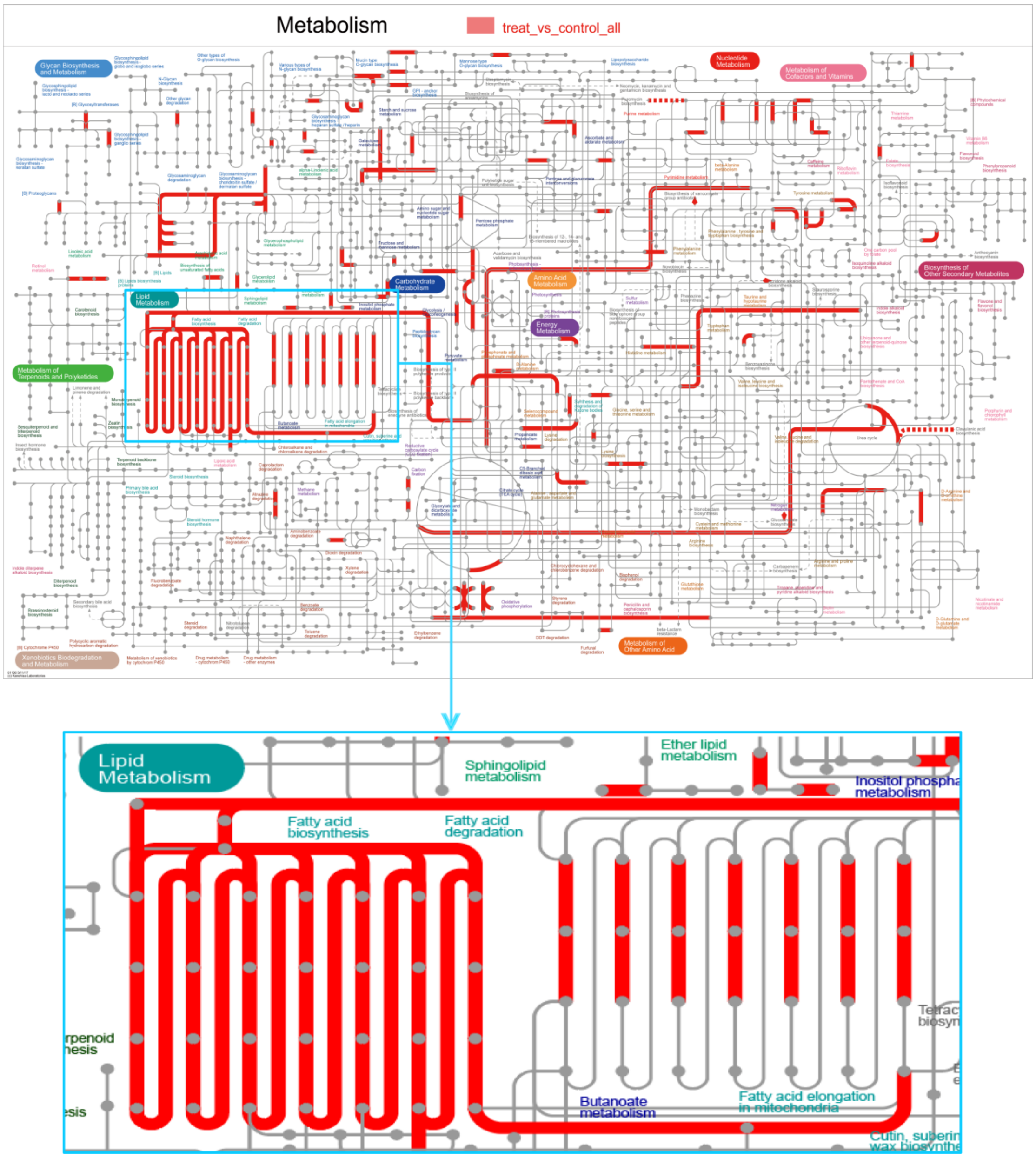

Supplementary Fig. 2 Enrichment analysis of metabolic pathway based on proteomic data from mass spectrumetry. Metabolic pathway enrichment is analyzed using the differentially expressed proteins in the comparison between WT mice (control) and FAM3A-transgenic mice (treat), both of which were fed with high-fat diet (HFD) for ten weeks (n=6 per group).

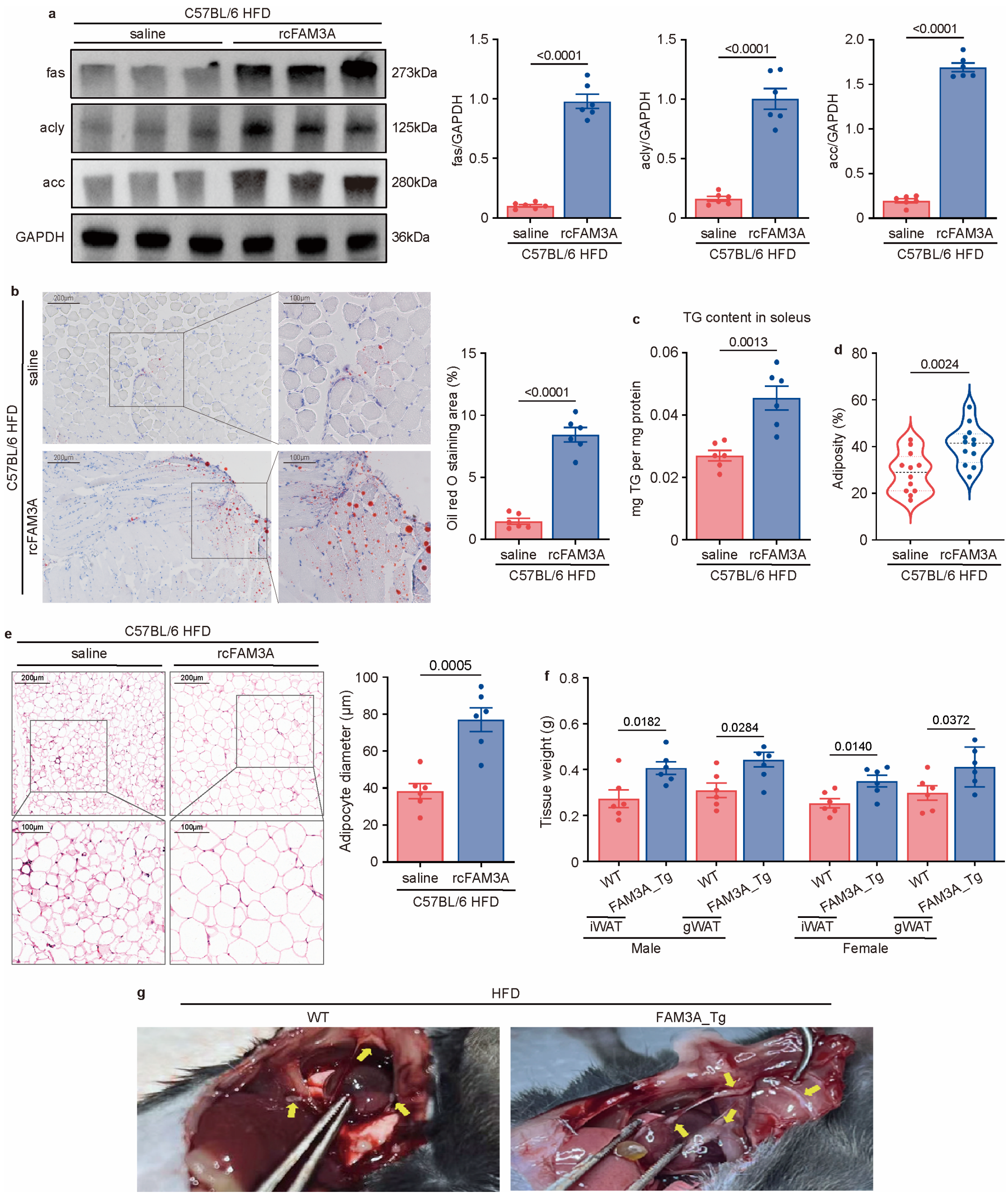

Supplementary Fig. 3 Lipogenesis following FAM3A interference. The C57BL/6 wild type (WT) mice were fed with HFD and meanwhile treated with or without recombinant FAM3A (rcFAM3A). **a** Western blot images and quantifications to evaluate the expression levels of fas, acly, and acc in soleus muscles (n=6 biologically independent animals/group; quantitative comparisons between samples were run on the same gel). **b,c** The lipid droplet detected by oil red O staining (b, n=5 biologically independent animals/group) and TG content (c, n=6 biologically independent animals/group) were measured and graphed in soleus muscles from the mice which were fed with HFD for five weeks. Scale bar: 200 μm, insets: 100 μm in b. **d,e** The body adiposity (d, n=12 biologically independent animals/group) and adipocyte diameter from gWAT (e, n=6 biologically independent animals/group) were measured and graphed in mice which were fed with HFD for ten weeks. **f** The inguinal white adipose tissue (iWAT) and gonadal white adipose tissue (gWAT) weight of WT and

FAM3A-transgenic mice including the males and the females, which were fed with HFD for five weeks (n=6 biologically independent animals/group). **g** The The representative anatomic images from C57BL/6 WT mice and FAM3A-transgenic mice were fed with HFD for five weeks. Yellow arrows indicate the fats. Data are presented as mean±SEM. Statistical significance was calculated with two-tailed independent *t* test and *P* values are indicated ($^{ns}P \geqslant 0.05$). Source data are provided as a Source Data file.

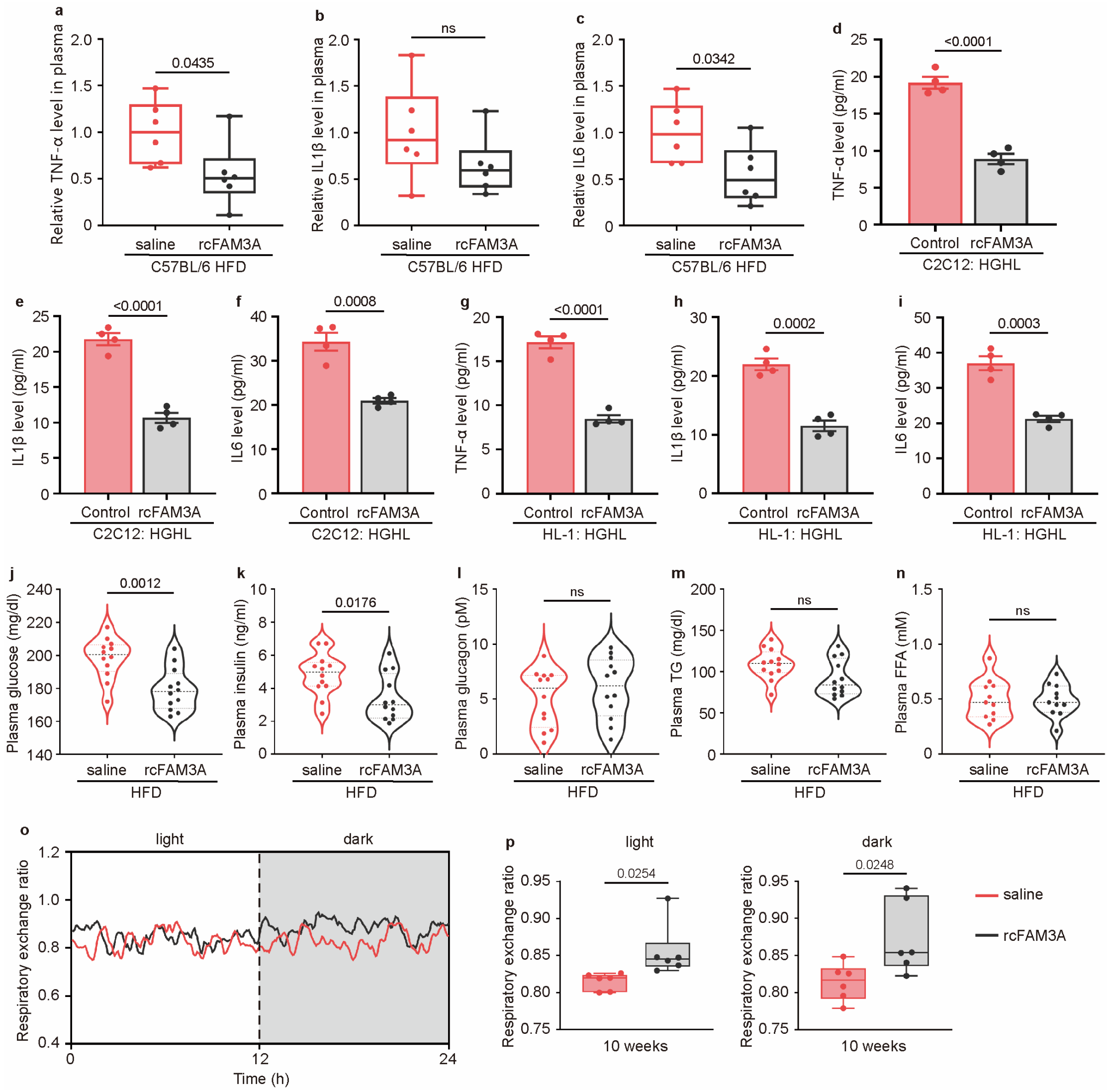

Supplementary Fig. 4 The metabolic and inflammatory characteristics following FAM3A interference. a-c The TNF-α (a), IL1β (b), and IL6 (c) levels in plasma were measured and graphed from the C57BL/6 mice which were fed with HFD for sixteen weeks and meanwhile treated with or without rcFAM3A (n=6 biologically independent animals/group). **d-i** The C2C12 and HL-1 cells were treated with rcFAM3A (200 ng/ml) for 12 hours under HGHL conditions. The TNF-α (d, g), IL1β (e, h), and IL6 (f, i) levels in the culture medium were measured and graphed (n=4 biologically independent samples/group). **j-n** The plasma glucose (j), insulin (k), glucagon (l), triglyceride (TG, m), and free fatty acid (FFA, n) levels were measured and graphed in the mice treated as in a (n=12 biologically independent animals/group). **o, p** The respiratory exchange ratio was measured and graphed in the mice treated as in a (n=6 biologically independent animals/group). Data are presented as mean+SEM. Statistical significance was calculated with two-tailed independent *t* test and *P* values are indicated ($^{ns}P \geqslant 0.05$). Source data are provided as a Source Data file.

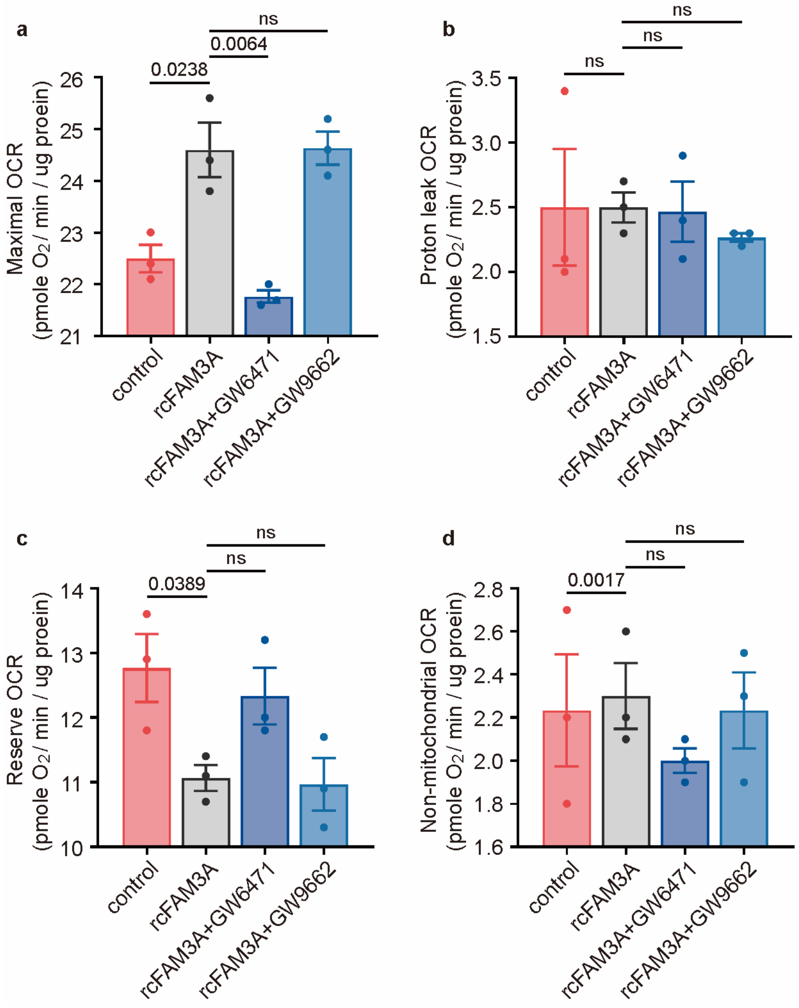

Supplementary Fig. 5 The mitochondrial function following FAM3A interferences. a-d The C2C12 myocytes were treated with rcFAM3A (200 ng/ml) for 8 hours. The cellular maximal (a), proton leak (b), reserve (c), and non-mitochondrial (d) OCR were quantified and graphed (n=3 biologically independent samples/group). Data are presented as mean+SEM. Statistical significance was calculated with two-tailed independent *t* test and *P* values are indicated ($^{ns}P \geqslant 0.05$). Source data are provided as a Source Data file.

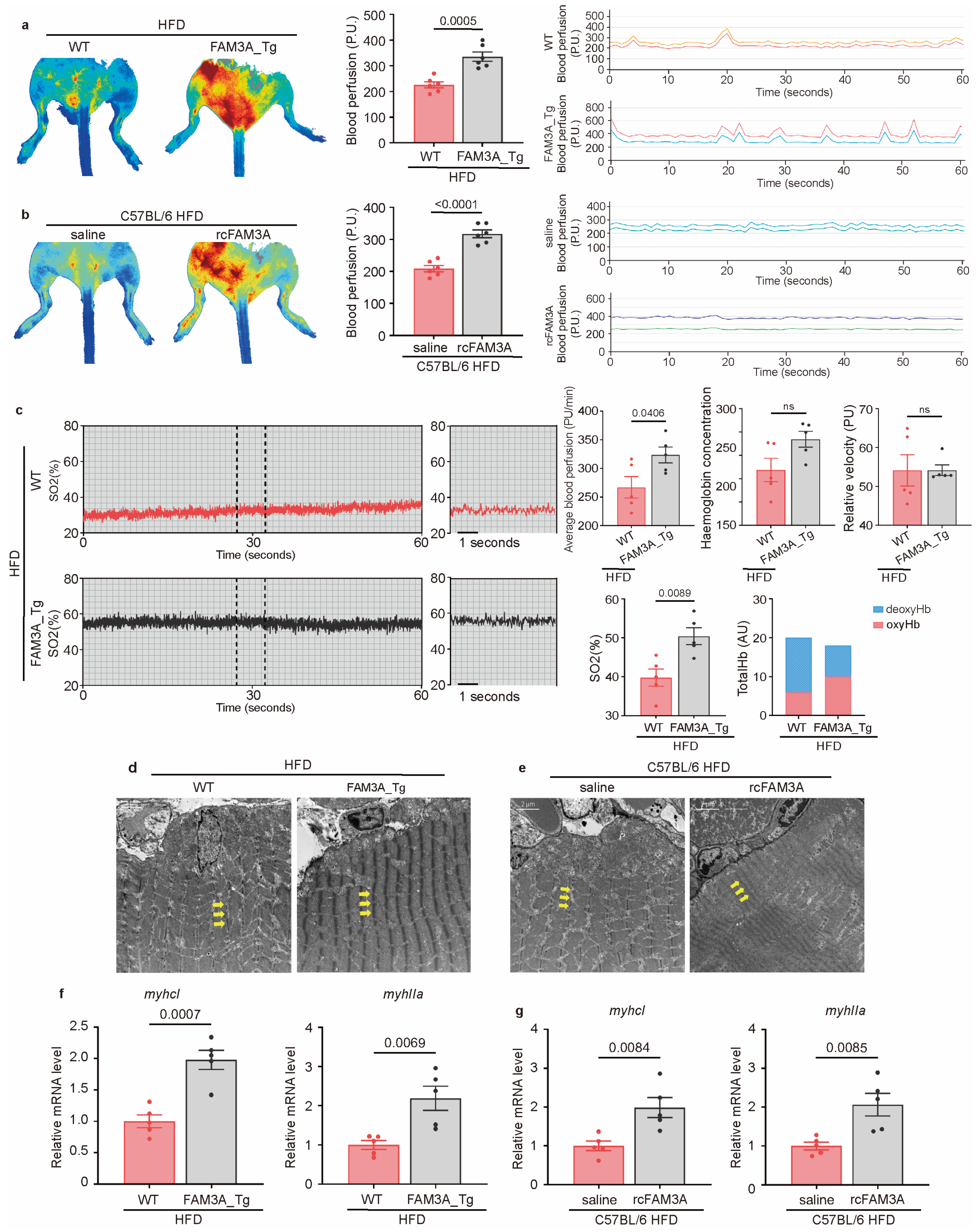

Supplementary Fig. 6 The blood flux characteristics in the hind limb and muscle homeostasis following FAM3A interferences. The mice were fed with HFD for ten weeks and meanwhile treated with or without rcFAM3A. **a, b**, The blood perfusion in the hind limb was measured and graphed (n=6 biologically independent animals/group). **c** The hemoglobin oxygen saturation (SO_2) in blood were measured and graphed (n=6 biologically independent animals/group). **d, e** The soleus muscle sarcomere morphology imaged by an electron microscope were shown, and Z-disc were indicate by the yellow arrows. Scale bar: 2 μm. **f, g** The mRNA levels of *myhcI* and *myhIIa* in the soleus tissues were measured and graphed (n=6 biologically independent animals/group). Data are presented as mean+SEM. Statistical significance was calculated with two-tailed independent *t* test and *P* values are indicated ($^{ns}P \geq 0.05$). Source data are provided as a Source Data file.

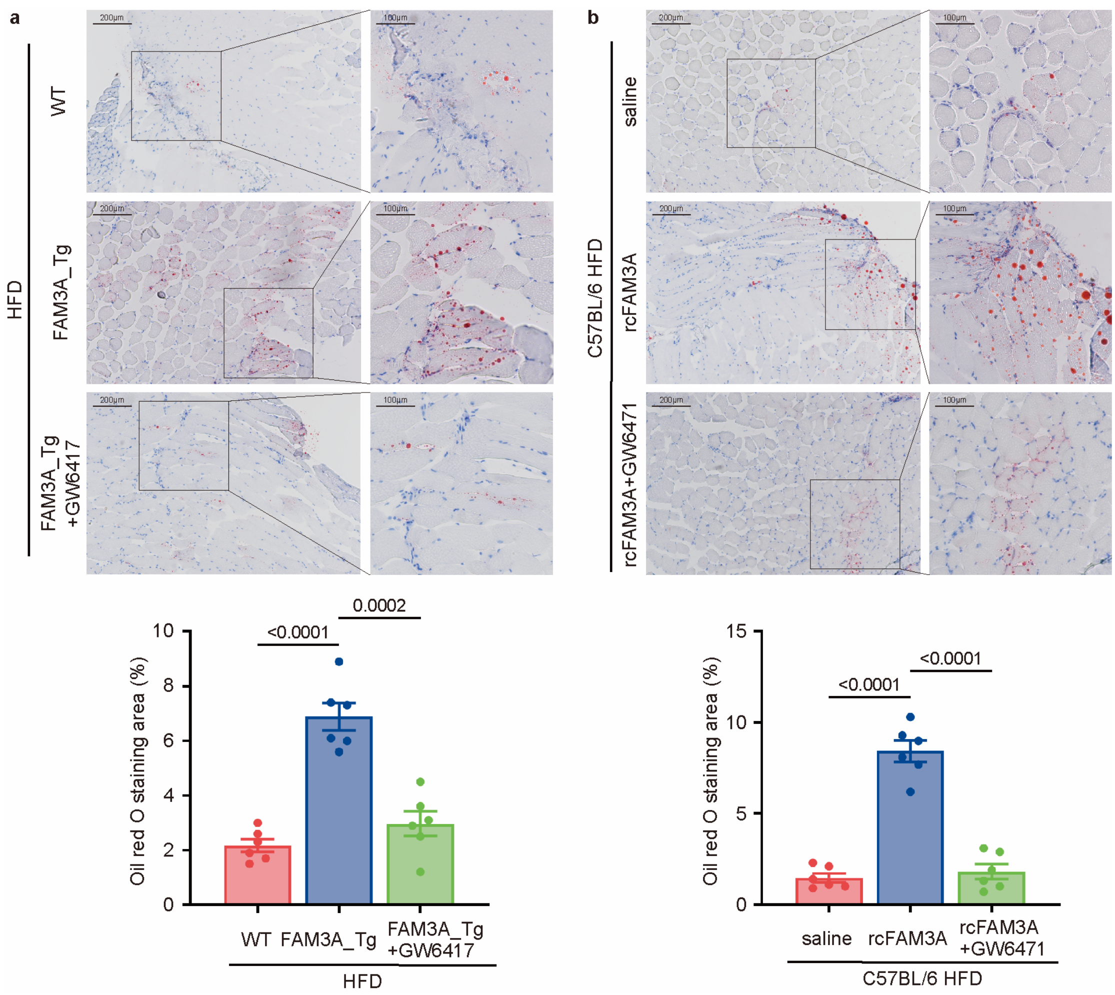

Supplementary Fig. 7 Lipogenesis following GW6471 interference. The oil-red staining in soleus muscles from FAM3A-transgenic mice (a) or rcFAM3A-infused mice (b) which were fed with HFD for five weeks and meanwhile treated with GW6471 (n=6 biologically independent animals/group). Scale bar: 200 μm, insets: 100 μm in c.

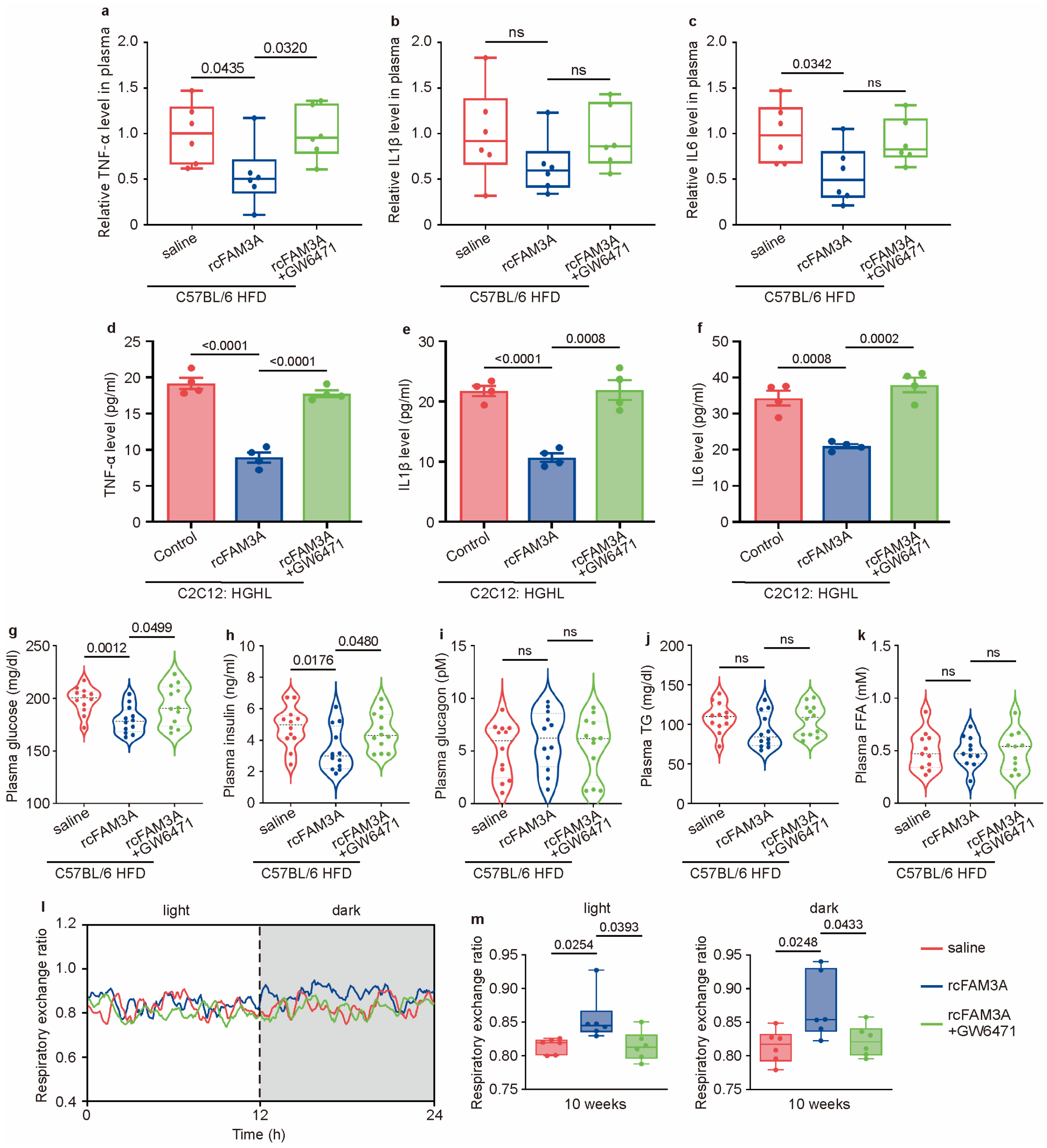

Supplementary Fig. 8 FAM3A-PPARα signaling affects the metabolic and inflammatory characteristics in mice treated with rcFAM3A. a-c The TNF-α (a), IL1β (b), and IL6 (c) levels in soleus muscles were measured and graphed in the rcFAM3A-infused C57BL/6 mice which were treated with GW6471 and fed with HFD for sixteen weeks (n=6 biologically independent animals/group). **d-f** The C2C12 cells were pre-treated with GW6471 and then stimulated with rcFAM3A (200 ng/ml) for 12 hours under HGHL conditions. The TNF-α (d), IL1β (e), and IL6 (f) levels in the culture medium were measured and graphed (n=4 biologically independent samples/group). **g-k** The plasma fasting glucose (g), insulin (h), glucagon (i), triglyceride (TG, j), and free fatty acid (FFA, k) levels were measured and graphed in the mice treated as in a (n=12 biologically independent animals/group). **l, m** The respiratory exchange ratio was measured and graphed in the mice fed with HFD for ten weeks (n=6 biologically independent animals/group). Data are presented as mean+SEM. Statistical significance was calculated with two-tailed independent *t* test and *P* values are indicated (^{ns}P ⩾0. 05). Source data are provided as a Source Data file.

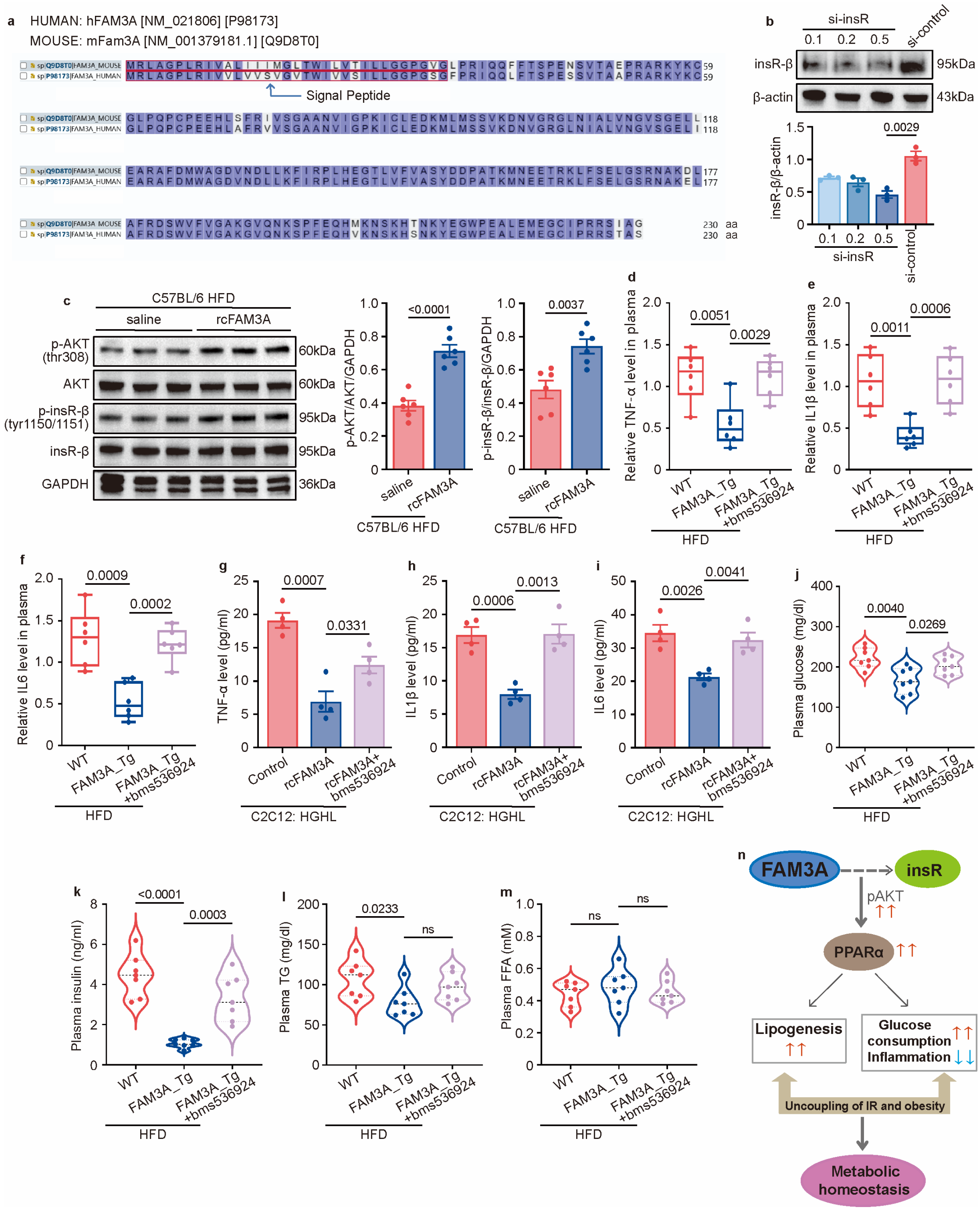

Supplementary Fig. 9 The effects of FAM3A-PPARα signaling on metabolism and inflammation is dependent on insulin receptor. a The human and mouse FAM3A amino acid sequences. The red box indicates signal peptide. **b** Western blot images and quantifications to evaluate the expression level of insR-β in cells transfected with insulin receptor (insR) siRNA with the indicated concentrations (μg/ml, n=3 biologically independent samples/group; quantitative comparisons between samples were run on the same gel). **c** Western blot images and quantifications to evaluate the expression levels of p-AKT and p-insR-β in the mice fed with HFD for ten weeks and treated with or without rcFAM3A (n=6 biologically independent animals/group). **d-f** The levels of TNF-α (d), IL1β (e), and IL6 (f) in soleus muscles were measured and graphed in the mice treated with bms536924 and fed with HFD for sixteen weeks (n=6 biologically independent animals/group). **g-i** The C2C12 cells

were pre-treated bms536924 and then stimulated with rcFAM3A (200 ng/ml) for 12 hours under HGHL conditions. The TNF-α (g), IL1β (h), and IL6 (i) levels in the culture medium were measured and graphed (n=4 biologically independent samples/group). **j-m** The plasma glucose (j), insulin (k), triglyceride (TG, l), and free fatty acid (FFA, m) levels were measured and graphed in the mice fed with HFD for sixteen weeks (n=7 biologically independent animals/group). **n** The schematic image illustrates the role of FAM3A in regulating metabolic homeostasis. FAM3A enhances AKT signaling and PPARα level depending on insulin receptor (insR). Subsequently, FAM3A-PPARα signaling promotes lipogenesis, but it gives impetus to glucose consumption and suppresses inflammation, leading to uncoupling of insulin resistance (IR) and obesity, and ultimately maintaining of metabolic homeostasis. Data are presented as mean+SEM. Statistical significance was calculated with two-tailed independent t test and P values are indicated ($^{ns}P \geqslant 0.05$). Source data are provided as a Source Data file.

Supplementary Tables

Supplementary Table 1 Clinical characteristics of varicosity patients without (Control) or with metabolic disorders (MD)

Terms	Control (n=26)	MD (n=22)	*P* value[&]
Sex: Male, n (%)	20 (76.9)	17 (77.3)	1
Age, years (mean±SEM)	61.35±5.92	61.91±5.53	1
BMI, kg/m^2 (mean±SEM)	13.73±1.64	13.42±2.22	1
Diabetes mellitus, n (%)	0 (0)	17 (77.3)	<0.0001****
Hyperlipidemia, n (%)	0 (0)	10 (45.5)	<0.0001****
Obesity, n (%)	0 (0)	1 (4.5)	<0.001***
Atherosclerosis, n (%)	0 (0)	0 (0)	-
CAD[#], n (%)	6 (23.1)	5 (22.7)	1
Hypertension, n (%)	15 (57.7)	13 (59.1)	1
Smoking, n (%)	8 (30.8)	6 (27.3)	0.92
Alcohol, n (%)	10(38.5)	9 (40.9)	0.87
Stroke, n (%)	0 (0)	0 (0)	-

CAD: coronary artery disease; BMI: body mass index; &: Statistical significance of Age was calculated with two-tailed independent *t* test and others were derived from Fisher's exact test, and *P* values are presented. ***marks significant difference with *P*<0. 001; ****marks significant difference with *P*<0. 0001.

Supplementary Table 2 Primers for Quantitative RT-PCR

Genes	Species	Forward Primer	Reverse Primer
adipoq	M	TGGAAGCTTGTCTCCAGTGA	TCGACTTTCCATCCCACTTC
fabp5	M	CAAAACCGAGAGCACAGTGA	CCCTCATTGCACCTTCTCAT
fatp3	M	AGTTGTGCCGATACCTCGTCGA	CCTCTGTCATGCCATACGTCTC
fatp4	M	GACTTCTCCAGCCGTTTCCACA	CAAAGGACAGGATGCGGCTATTG
lpl	M	TTTGGCTCCAGAGTTTGACC	GTCTTGCTGCTGTGGTTGAA
gpihbp1	M	GGGCACAAGAAGATGGTGAT	CTGGAGCAGCTCTGTGTCTG
acaca	M	TGAACTTCACACAGGTAGTCTGCC	TGGAACACTCGATGGAGTTTCT
acacb	M	TGACAGACTGATCGCAGAGAAAG	TGGAGAGCCCCACACACA
fasn	M	GGCATCATTGGGCACTCCTT	GCTGCAAGCACAGCCTCTCT
scd1	M	TTCCCTCCTGCAAGCTCTAC	CAGAGCGCTGGTCATGTAGT
acly	M	GCCAGCGGGAGCACATC	CTTTGCAGGTGCCACTTCATC

Supplementary Table 3 Plasma circulating biomarkers influenced by FAM3A (FAM3A-transgenic mice vs. C57BL/6J controls under HFD circumstance)

Entrez ID	Protein name	Fold change	Description	P.Value
56636	FGF-21	0.71276867	fibroblast growth factor 21	0.03074876
14130	Fcg RIIB	2.18517904	Fc receptor, IgG, low affinity IIb (Fcgr2b)	0.00031211
12985	G-CSF	1.75667436	colony stimulating factor 3 (granulocyte) (Csf3)	0.00114246
16005	Igfals	0.53994292	insulin-like growth factor binding protein, acid labile subunit	0.04217796
16006	Igfbp1	1.87632471	insulin-like growth factor binding protein 1	0.03246745
257630	IL-17F	1.6192842	interleukin 17F	0.00387105
60505	IL-21	1.46798503	interleukin 21	0.00181775
21937	TNF RI	0.43277885	tumor necrosis factor receptor superfamily, member 1a (Tnfrsf1a)	0.00456322
22329	VCAM-1	2.38672214	vascular cell adhesion molecule 1 (Vcam1)	0.048467
16542	VEGF R2	2.38672214	kinase insert domain protein receptor (Kdr)	0.0048467

Major Resources Information

Supplementary Table 4 Experimental animals

Species/Strain	Vendor or Source	Background Strain	Sex
Wild type C57BL/6J	Vital River	C57BL/6J	male, female
FAM3A-transgenic mouse	Institute of Laboratory Animal Science, Chinese Academy of Medical Sciences	C57BL/6J	male, female

Supplementary Table 5 Cultured cells and related reagents

Cells or reagents	Vendor or Source	Catalog #
mouse myoblasts C2C12	Fenghui, China	CL0058
mouse cardiac myocytes HL-1	Cellverse, China	icell-m077
mouse 3T3-L1	Cellverse, China	icell-m066
human cardiac MVECs (hcMVECs)	Sciencell	6000
penicillin/streptomycin	Thermo Fisher Scientific	15140122
Antibiotic-Antimycotic	Thermo Fisher Scientific	15240062
DMEM	Thermo Fisher Scientific	11140035
ECM	Sciencell	1001
ECGS	Sciencell	1052
FBS	Sciencell	0025
P/S solution	Sciencell	0503

Supplementary Table 6 Antibodies

Target antigen	Vendor or Source	Catalog #
FAM3A (WB/IHC/IF)	Origene	TA324017
adiponectin (WB)	Cell Signaling Technology	2789
adiponectin (IHC)	Abcam	ab22554
srebp1 (WB)	Santa Cruz	sc-13551
fas (WB)	Cell Signaling Technology	3180
acly (WB)	Cell Signaling Technology	4332
acc (WB)	Cell Signaling Technology	3676
PPARα (WB)	Santa Cruz	sc-398394
PPARα (IHC)	Invitrogen	PA1-822A
p65 (WB)	Santa Cruz	sc-8008
pcna (WB)	Santa Cruz	sc-25280
AKT (WB)	Cell Signaling Technology	9272
p-AKT (thr308) (WB)	Cell Signaling Technology	9275
insR-β (WB)	Cell Signaling Technology	3025
p-insR-β (tyr1150/1151) (WB)	Cell Signaling Technology	3024
Calnexin	Abcam	ab75801
TSG101	Abcam	ab125011
CD9	Abcam	ab92726
GAPDH (WB)	Cell Signaling Technology	5174

β-actin (WB)	LABLEAD	A0101
DAPI (IF)	Origene	ZLI-9600
Goat anti-rabbit IgG (HRP-conjugated, WB/IHC/IF)	Proteintech	SA00001-2
Goat anti-mouse IgG (HRP-conjugated, WB/IHC/IF)	Proteintech	SA00001-1
CoraLite488-conjugated Goat anti-Rabbit IgG (IF)	Proteintech	SA00013-2
CoraLite594-conjugated Goat anti-Rabbit IgG (IF)	Proteintech	SA00013-4
CoraLite488-conjugated Goat anti-Mouse IgG (IF)	Proteintech	SA00013-1
CoraLite594-conjugated Goat anti-Mouse IgG (IF)	Proteintech	SA00013-3

WB: western blot; IF: immunofluorescence; IHC: immunohistochemistry.

Supplementary Table 7 Other important reagents and resources

Name	Vendor or Source	Catalog #
high-fat diet	Researchdiet	D12451
FAM3A ELISA Kit (mouse)	lifespan	LS-F17398
FAM3A ELISA Kit (human)	lifespan	LS-F35367
adiponectin ELISA Kit (mouse)	Abcam	Ab226900
adiponectin ELISA Kit (human)	lifespan	LS-F2599
Lipid Droplets Green Fluorescence Assay Kit with BODIPY 493/503	Beyotime	C2053
recombinant FAM3A protein	Origene	TP303495
GW6471	Topscience	T8486
bms536924	Topscience	T6419
Insulin Receptor siRNA (m)	Santa Cruz	sc-35673
TriglycerideELISA Kit (mouse)	Abcam	ab65336
free fatty acid ELISA Kit (mouse)	Abcam	ab65336
Glucose ELISA Kit (mouse)	Applygen	E1010
Insulin ELISA Kit (mouse)	CrystalChem	90080
Glucagon ELISA Kit (mouse)	Mercodia	10-1281-01
Minute^{TM} Plasma Membrane Protein Isolation andCell Fractionation Kit	Invent Biotechnologies	SM-005
Minute^{TM} Mitochondria Isolation Kit for Mammalian Cells and Tissues	Invent Biotechnologies	MP-007
ViraPower Adenoviral Gateway Expression Kit	Invitrogen	K4930-00
Nuclear extract kit	Beyotime	P0027
Triglyceride Colorimetric Assay	Cayman Chemical	10010303
Fatty Acid Oxidation Complete Assay Kit	Abcam	ab222944
ATP bioluminescence assay kit CLS II	Roche	11699695001
tetramethylrhodamine ethyl ester	Medchemexpress	115532-52-0
Complex I enzyme activity microplate assay kit	Abcam	ab109721
Complex II enzyme activity microplate assay kit	Abcam	ab109908
Complex IV enzyme activity microplate assay kit	Abcam	ab109911
Citrate synthase activity assay Kit	Abcam	ab239712
DNeasy Blood & Tissue Kit	Qiagen	69504
Carnitine palmitoyltransferase1 assay Kit	Bioss	AK491V
Oil red O Kit	Sigma	102419
MACS C tube	Miltenyi Biotec	130-096-334
digestion medium	Thermo Fisher Scientific	10829018
penicillin/streptomycin	Thermo Fisher Scientific	15140122

sodium pyruvate	Thermo Fisher Scientific	1360070
0.2% Collagenase IV	Worthington	LS004188
dispase	Thermo Fisher Scientific	171055-041
DNase I	Sigma-Aldrich	D4527-10KU
70 mm cell strainer	Sigma-Aldrich	CLS431751-50EA
40 mm cell strainer	Sigma-Aldrich	CLS431750-50EA
[1-^{14}C]-acetic acid	Perkin Elmer	NEC084H001MC
glass bottom dishes	Mattek Life Science	P35G-1.5-14-C
Bodipy 493/503	Thermo	D3922
Hoechst 33342	Thermo	H3570
