# Supplementary material for: FAM3A drives uncoupling of muscle lipid accumulation and insulin resistance depending on insulin receptor
Source: Cell Death Dis. 2025 Dec 7;17(1):72. doi: 10.1038/s41419-025-08298-1 (PMC12827484; doi:10.1038/s41419-025-08298-1)
Supplement: Supplementary file 2 — Uncropped blots [file 41419_2025_8298_MOESM2_ESM.pdf]

Uncropped Blots

1. Figure 2

Figure 2a

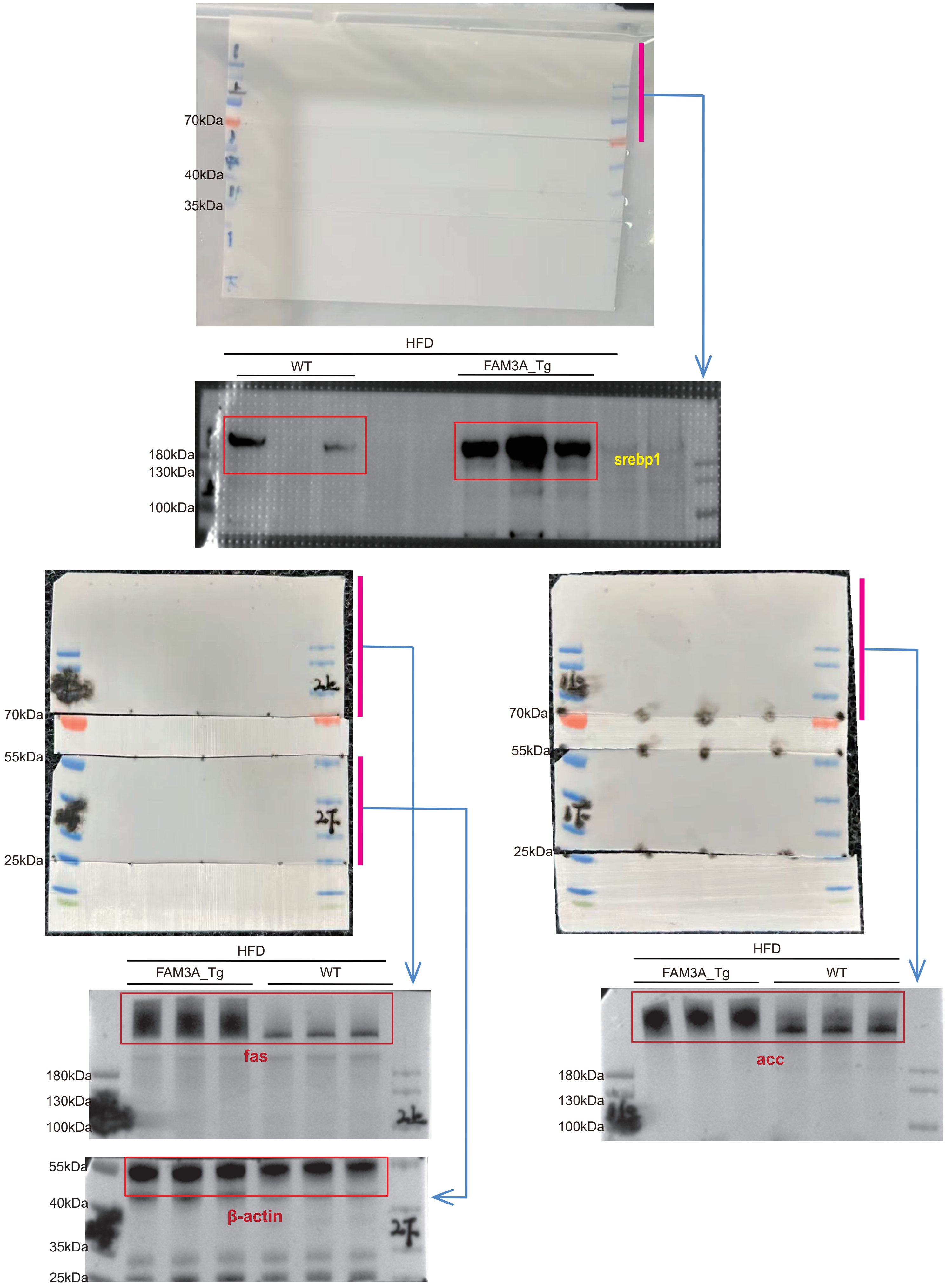

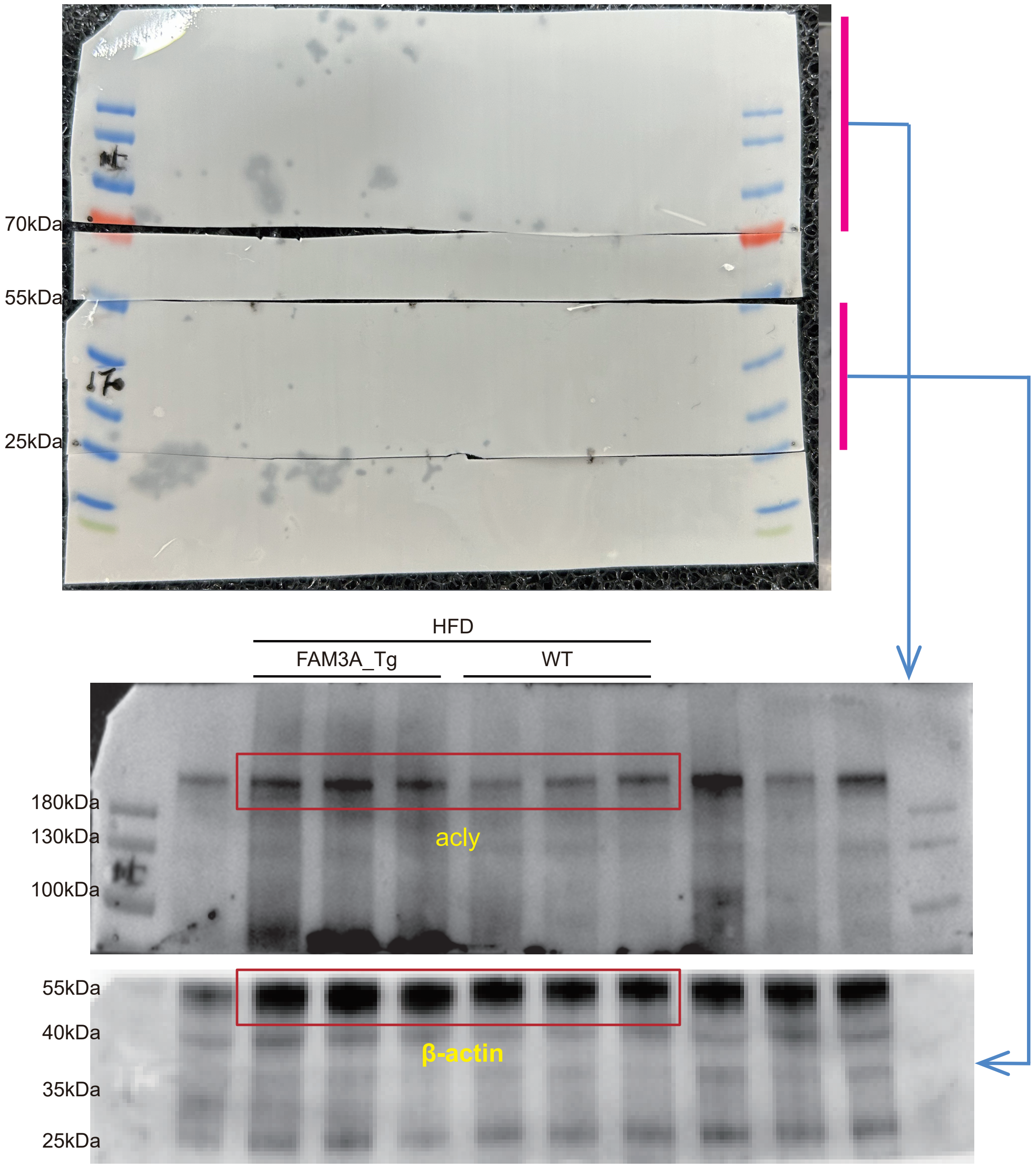

70kDa
55kDa
25kDa
HFD
FAM3A_Tg
WT
180kDa
130kDa
100kDa
acly
55kDa
40kDa
35kDa
25kDa
β-actin

Figure 2b

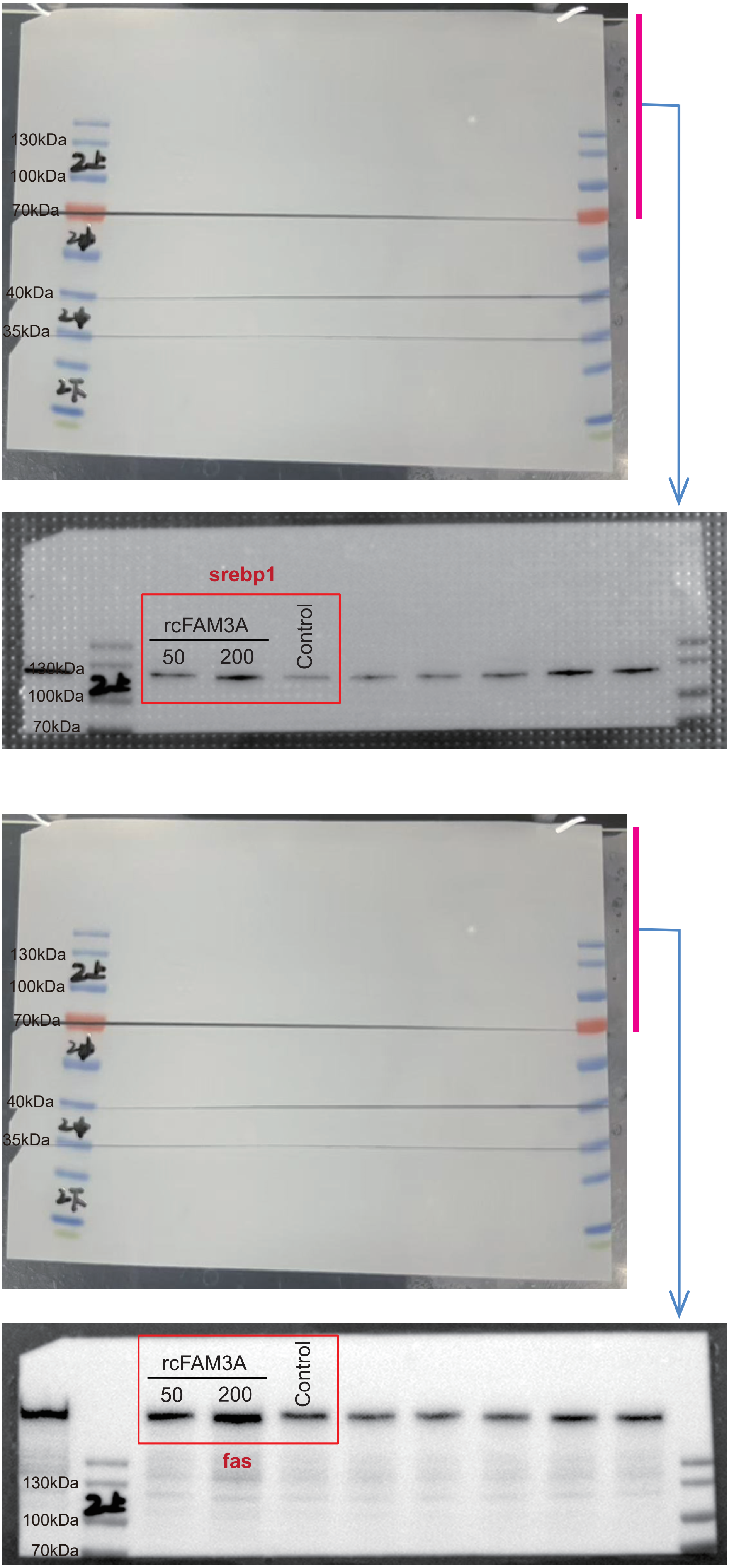
130kDa
100kDa
70kDa
40kDa
35kDa
srebp1
rcFAM3A
50
200
Control
130kDa
100kDa
70kDa
130kDa
100kDa
70kDa
40kDa
35kDa
rcFAM3A
50
200
Control
fas
130kDa
100kDa
70kDa

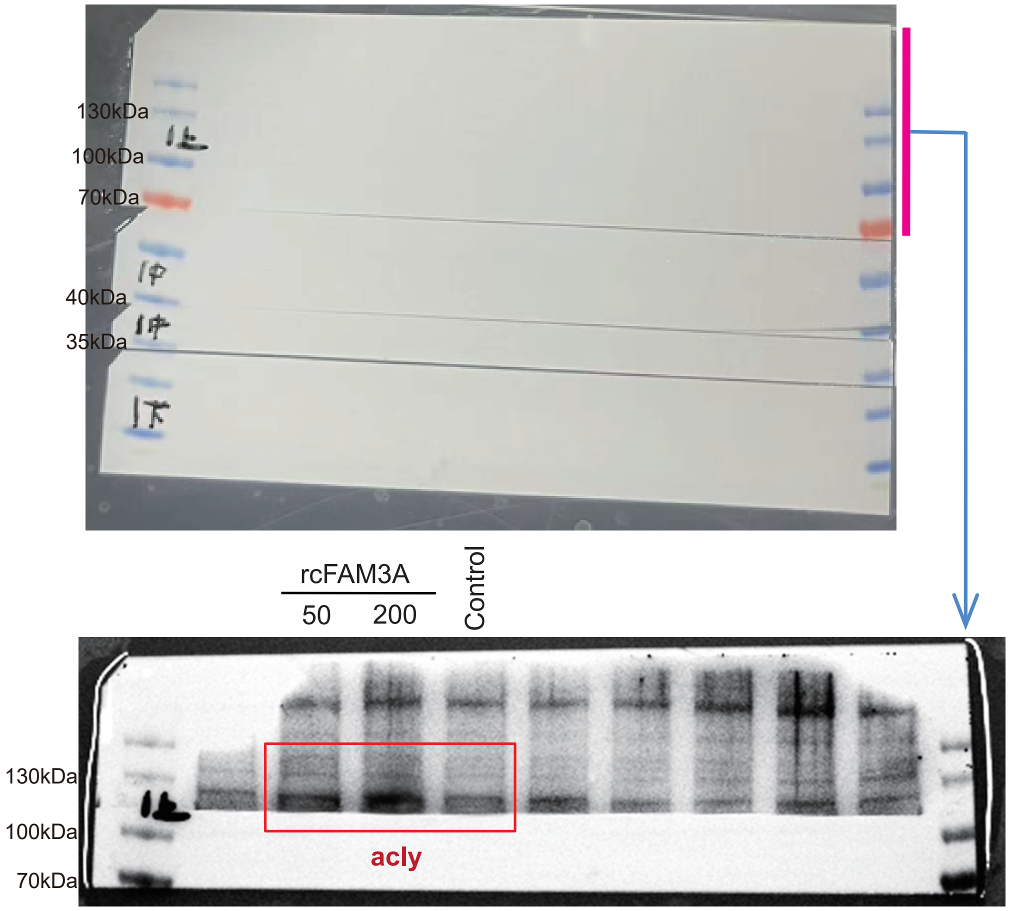
130kDa
100kDa
70kDa
40kDa
35kDa
rcFAM3A
50 200
Control
130kDa
100kDa
70kDa
acly

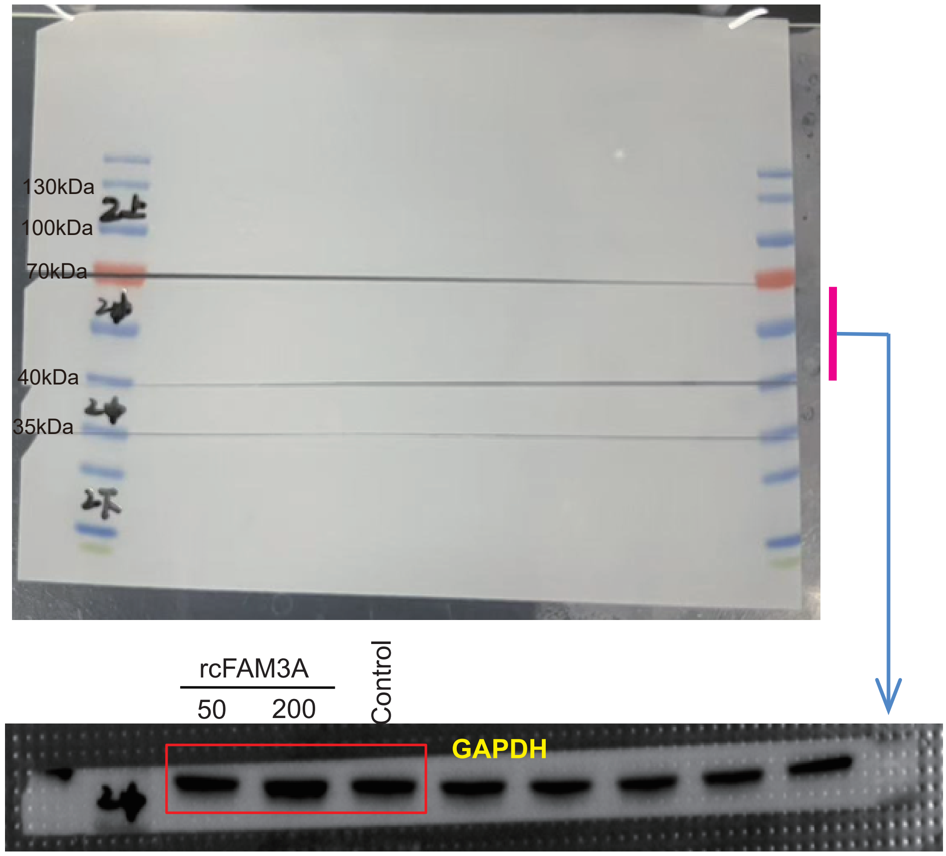
130kDa
100kDa
70kDa
40kDa
35kDa
rcFAM3A
50 200
Control
GAPDH

2. Figure 3

Figure 3a

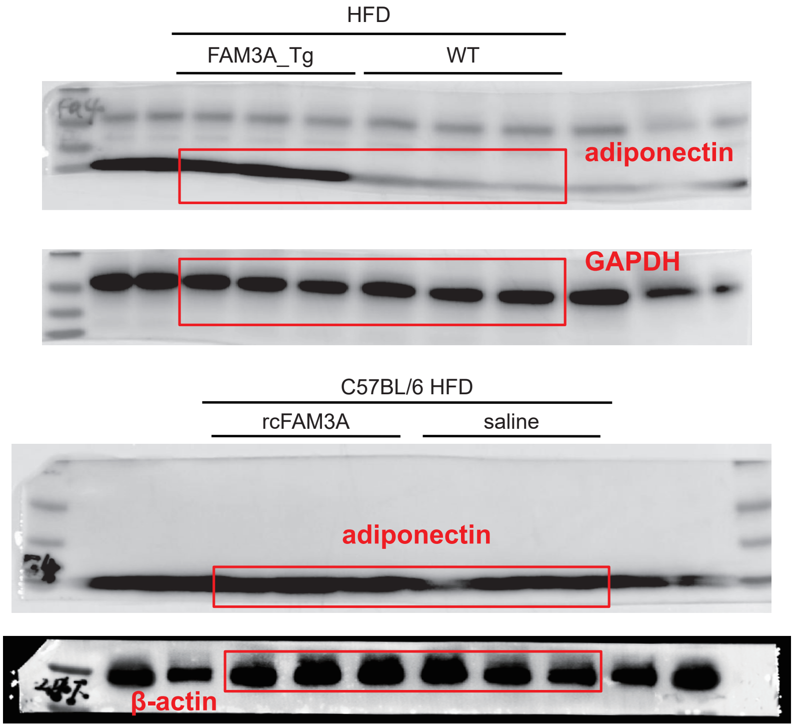

Figure 3b

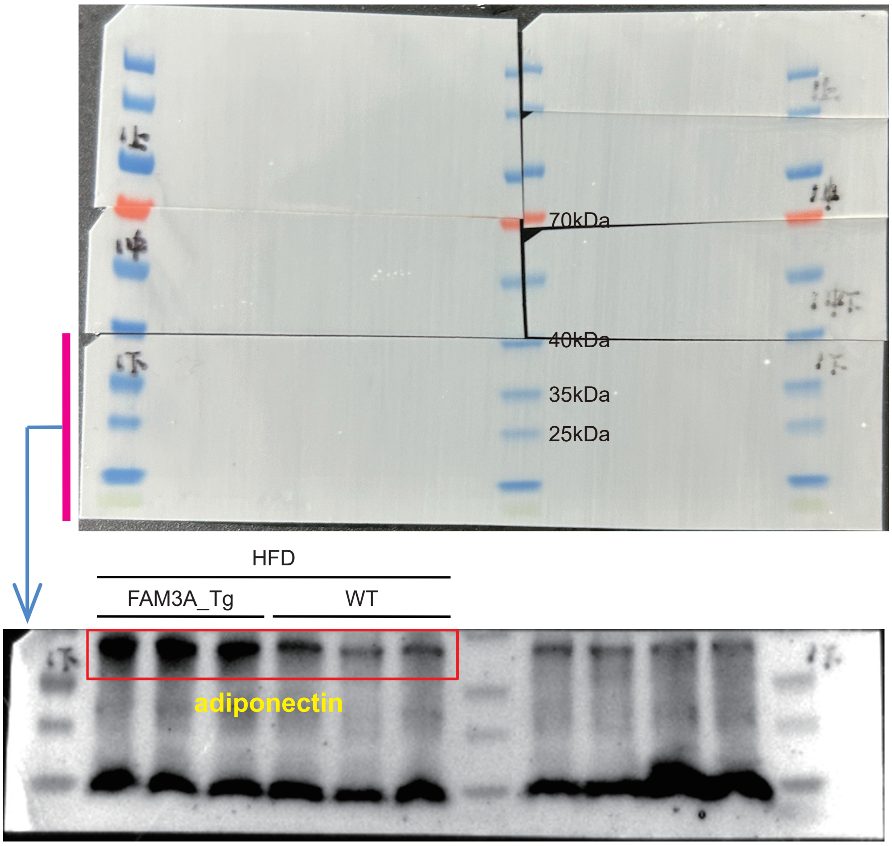

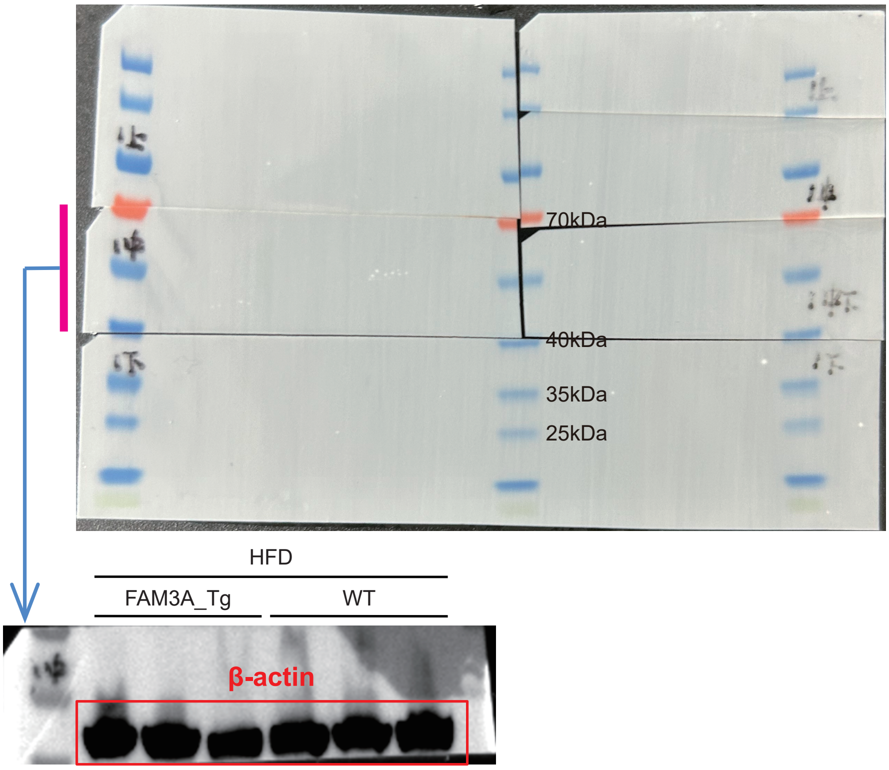
70kDa
40kDa
35kDa
25kDa
HFD
FAM3A_Tg
WT
β-actin

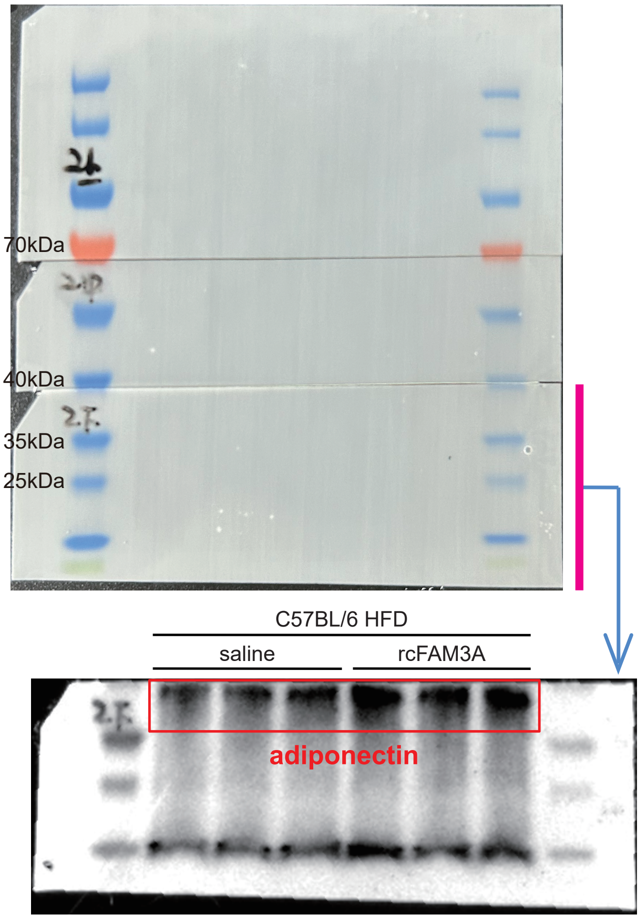
70kDa
40kDa
35kDa
25kDa
C57BL/6 HFD
saline
rcFAM3A
adiponectin

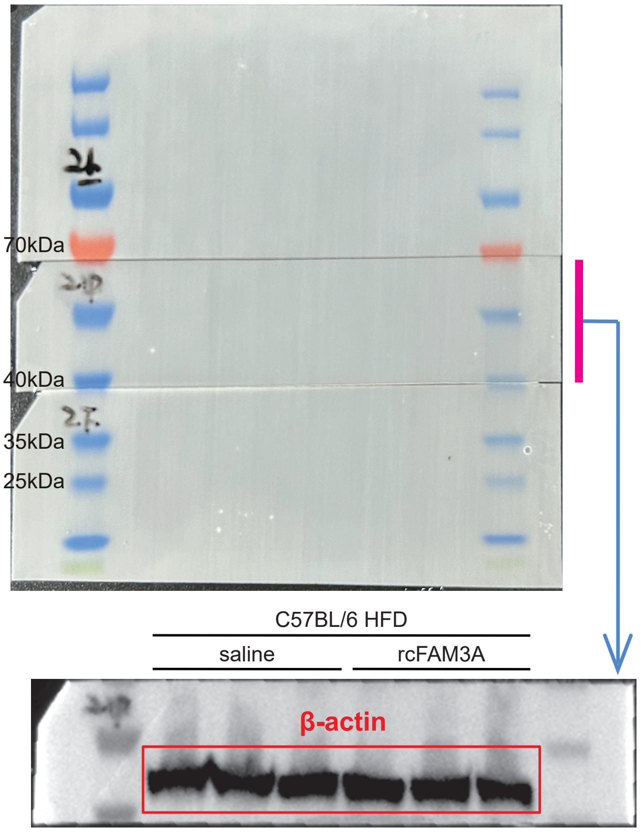
70kDa
40kDa
35kDa
25kDa
C57BL/6 HFD
saline
rcFAM3A
β-actin

Figure 3f

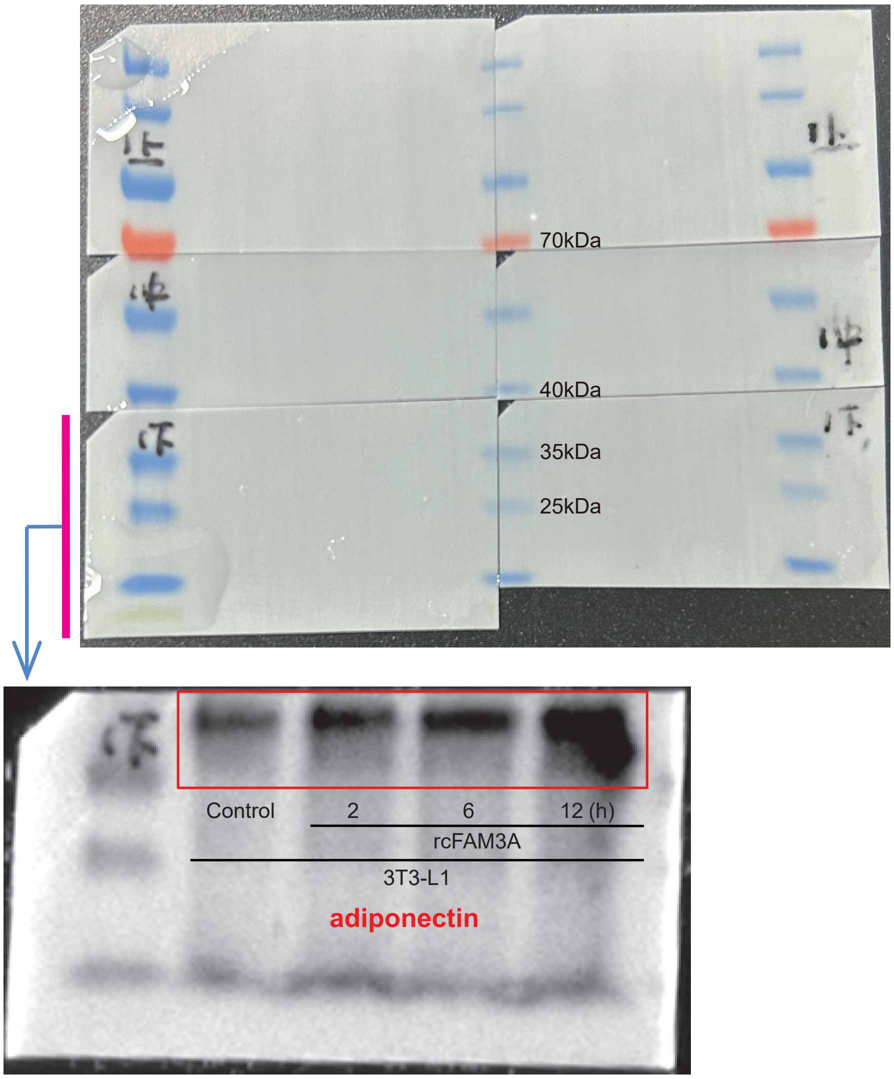
70kDa
40kDa
35kDa
25kDa
Control
2
6
12 (h)
rcFAM3A
3T3-L1
adiponectin

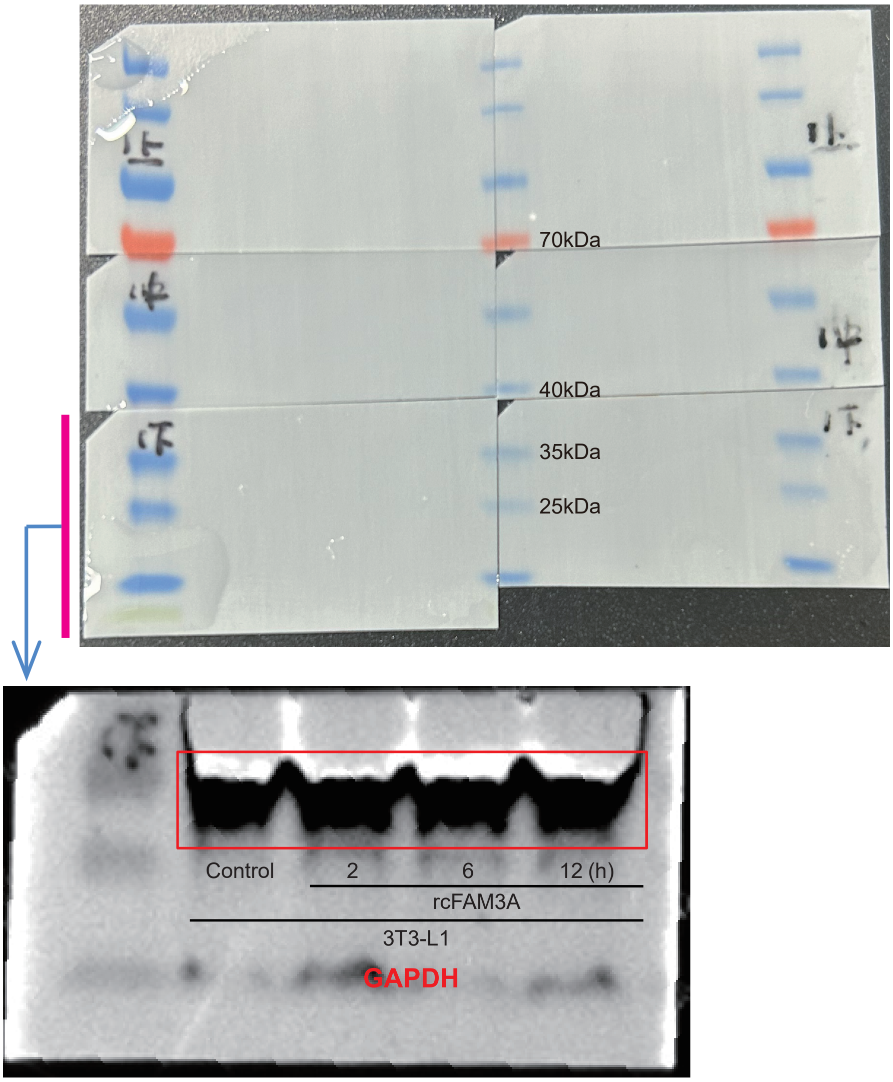

70kDa
40kDa
35kDa
25kDa
Control
2
6
12 (h)
rcFAM3A
3T3-L1
GAPDH

3. Figure 6

Figure 6a

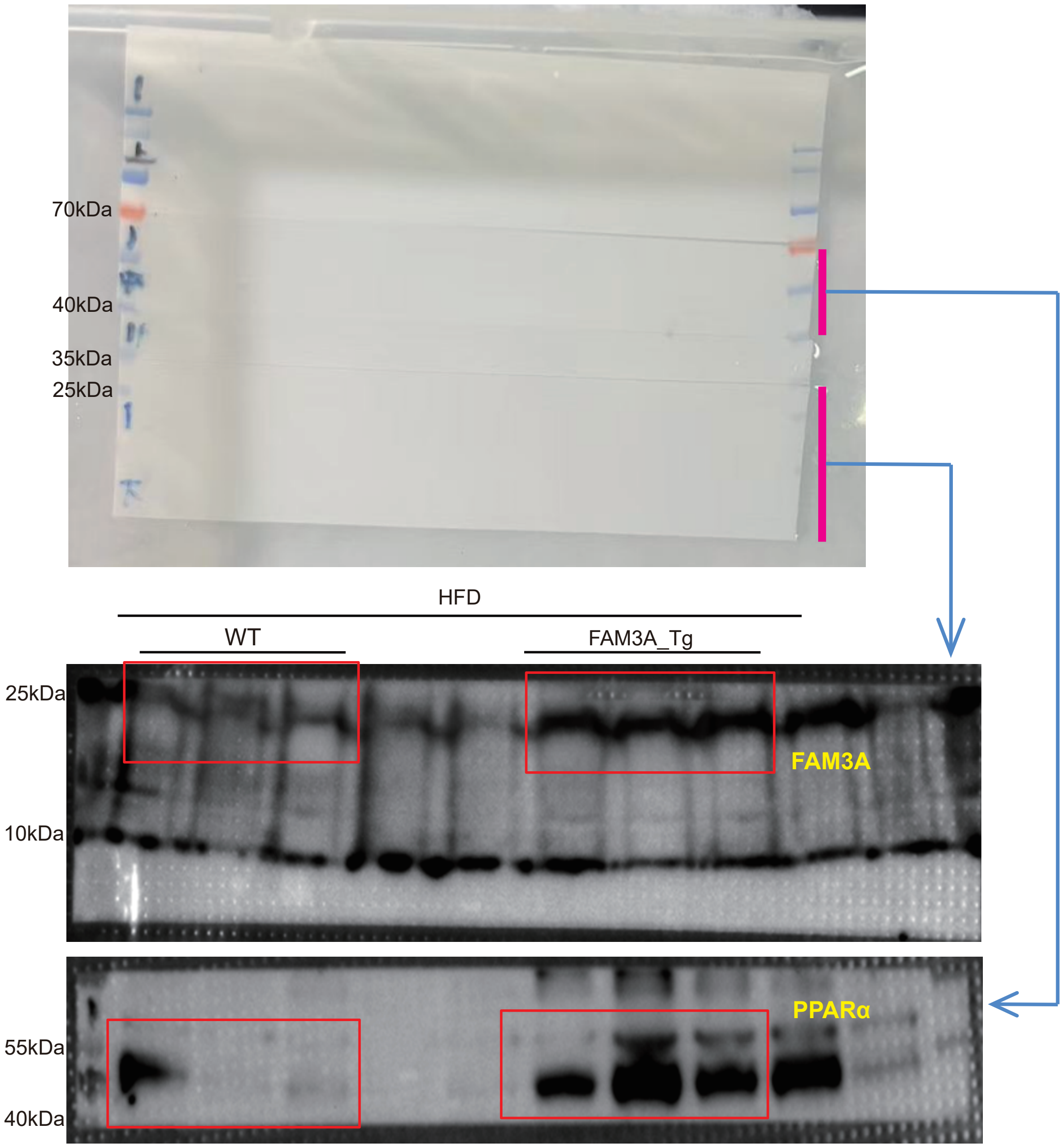

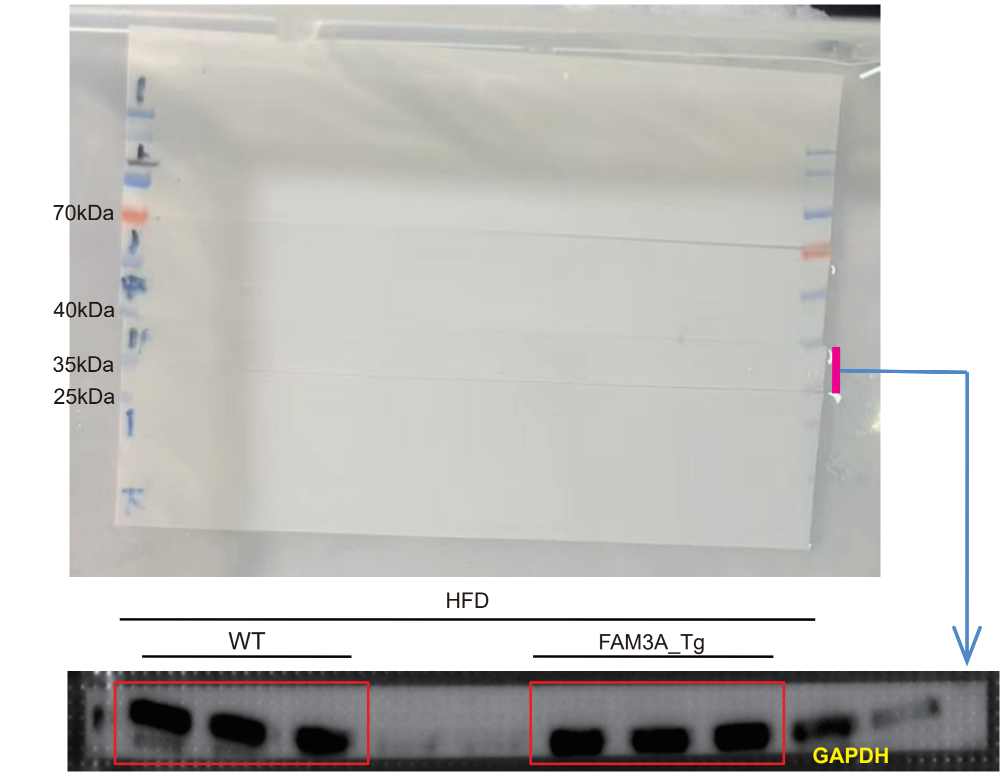

Figure 6d

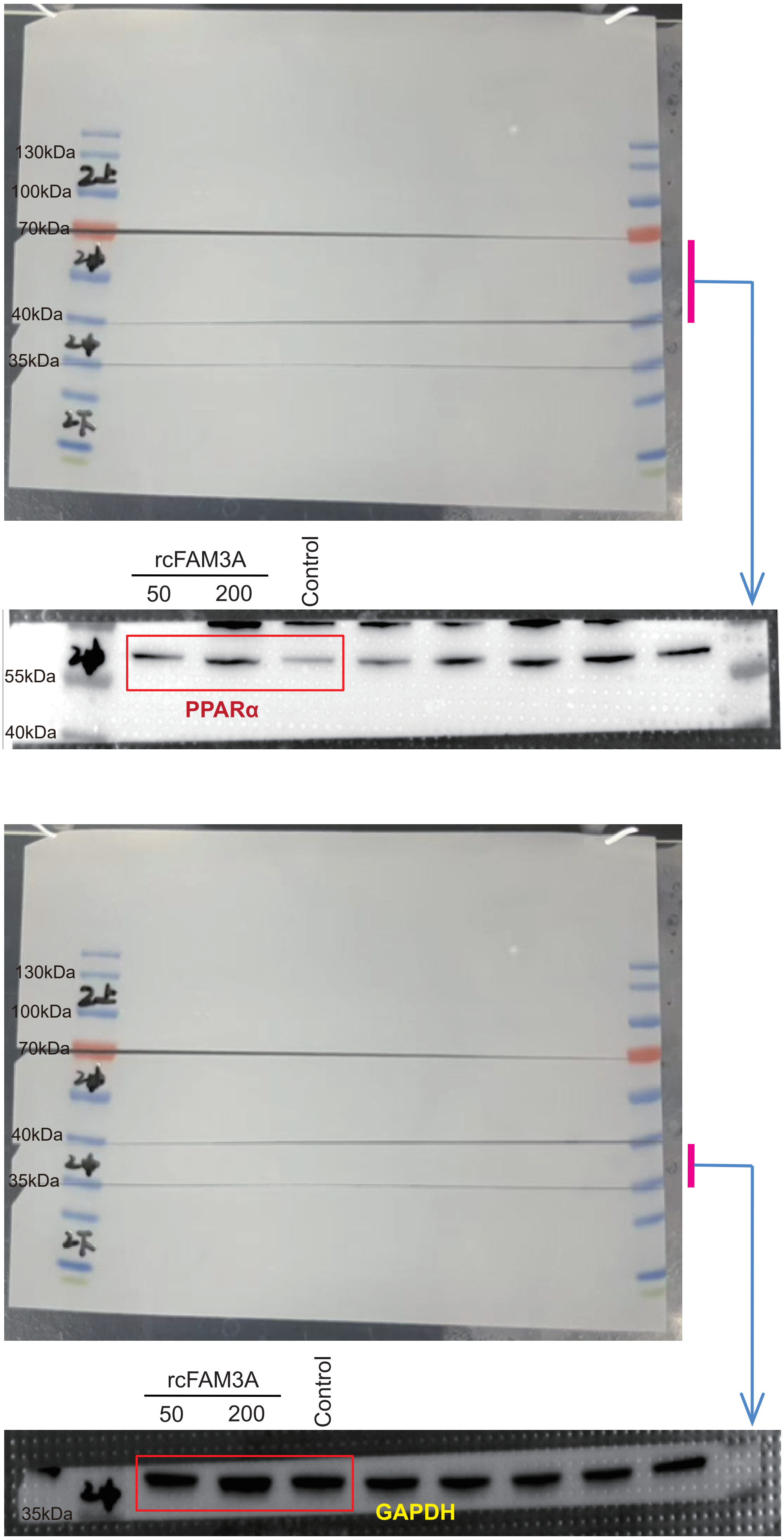

The GAPDH is same as Fig2b
due to a same western blot assay.

Figure 6e

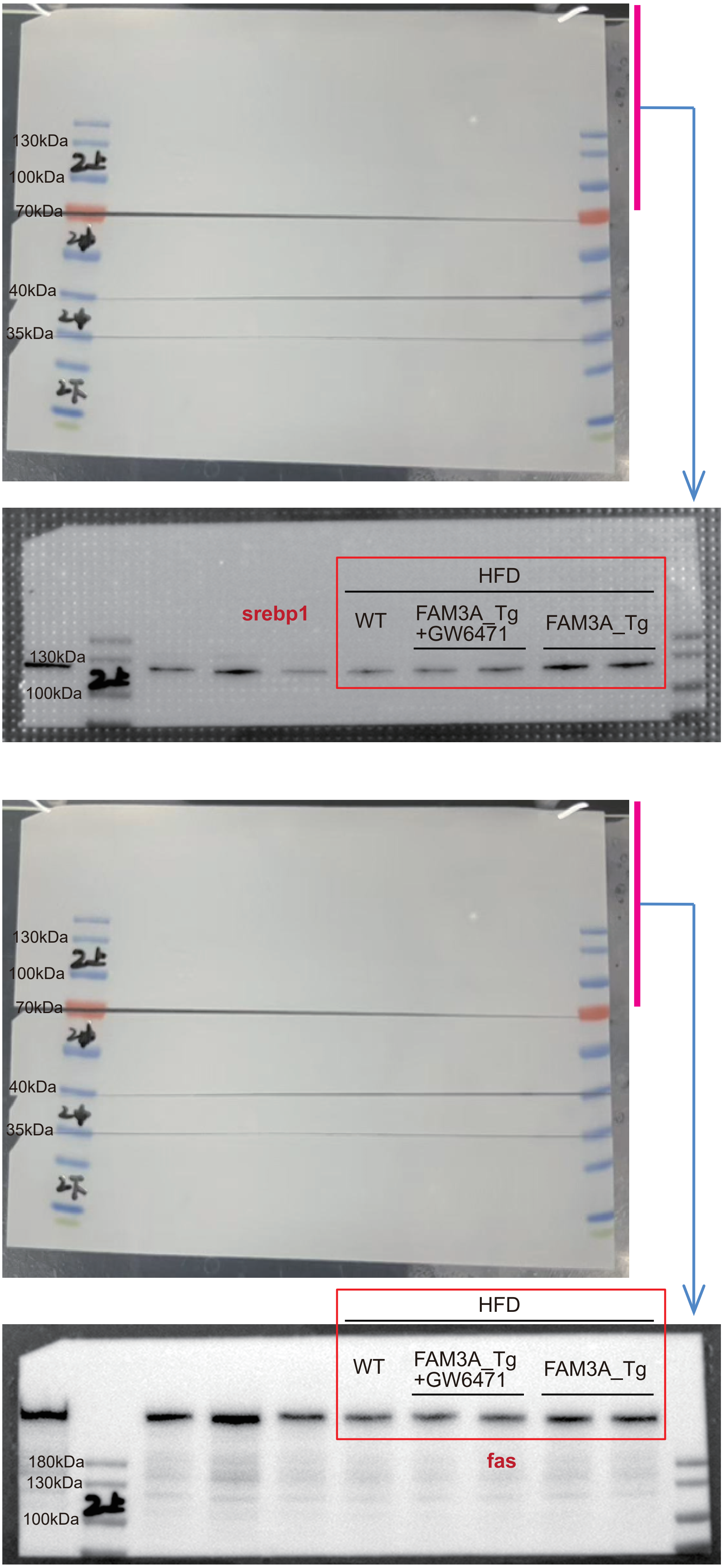
130kDa
100kDa
70kDa
40kDa
35kDa
srebp1
HFD
WT
FAM3A_Tg
+GW6471
FAM3A_Tg
130kDa
100kDa
130kDa
100kDa
70kDa
40kDa
35kDa
HFD
WT
FAM3A_Tg
+GW6471
FAM3A_Tg
fas
180kDa
130kDa
100kDa

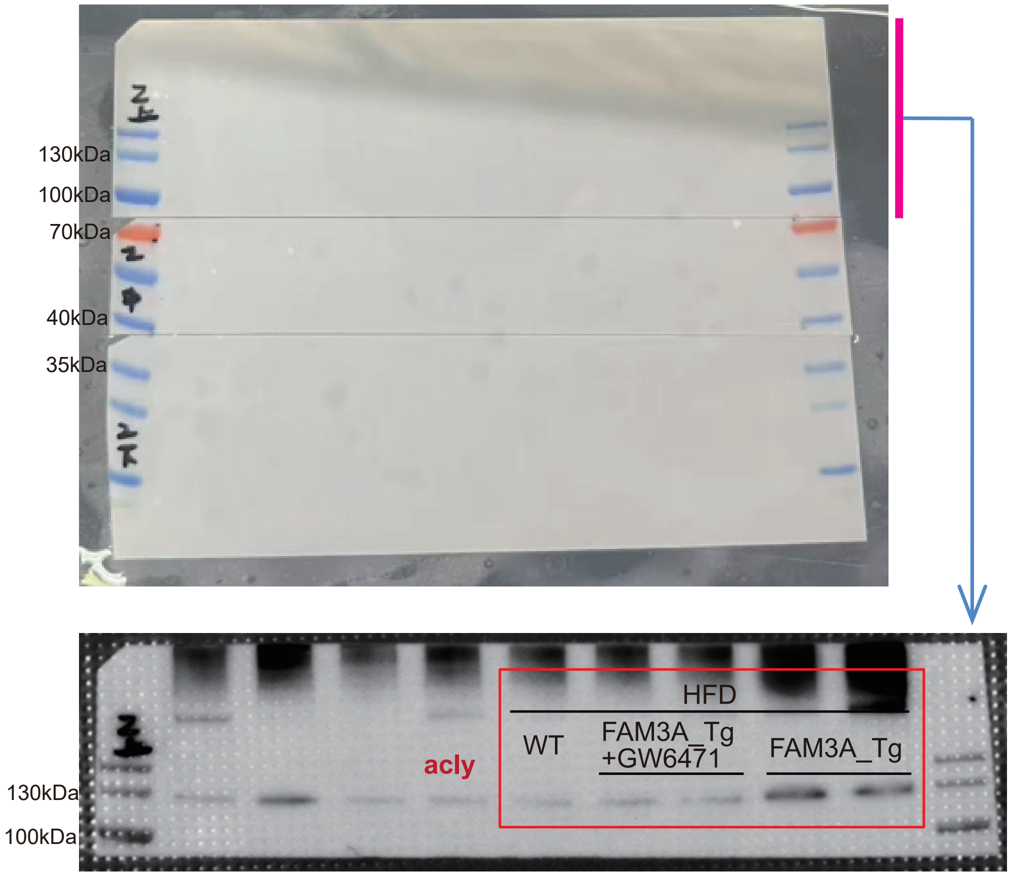
130kDa
100kDa
70kDa
40kDa
35kDa
HFD
WT
FAM3A_Tg
+GW6471
FAM3A_Tg
acly
130kDa
100kDa

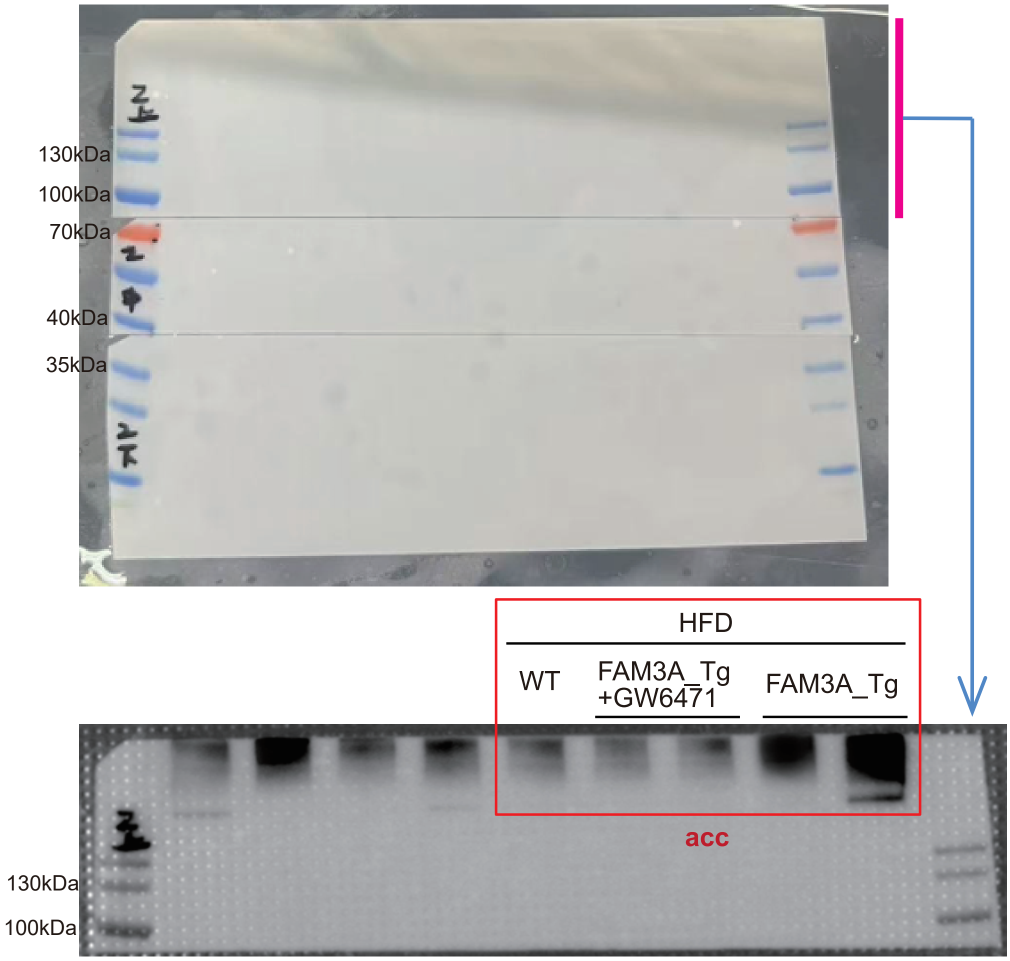
130kDa
100kDa
70kDa
40kDa
35kDa
HFD
WT
FAM3A_Tg
+GW6471
FAM3A_Tg
acc
130kDa
100kDa

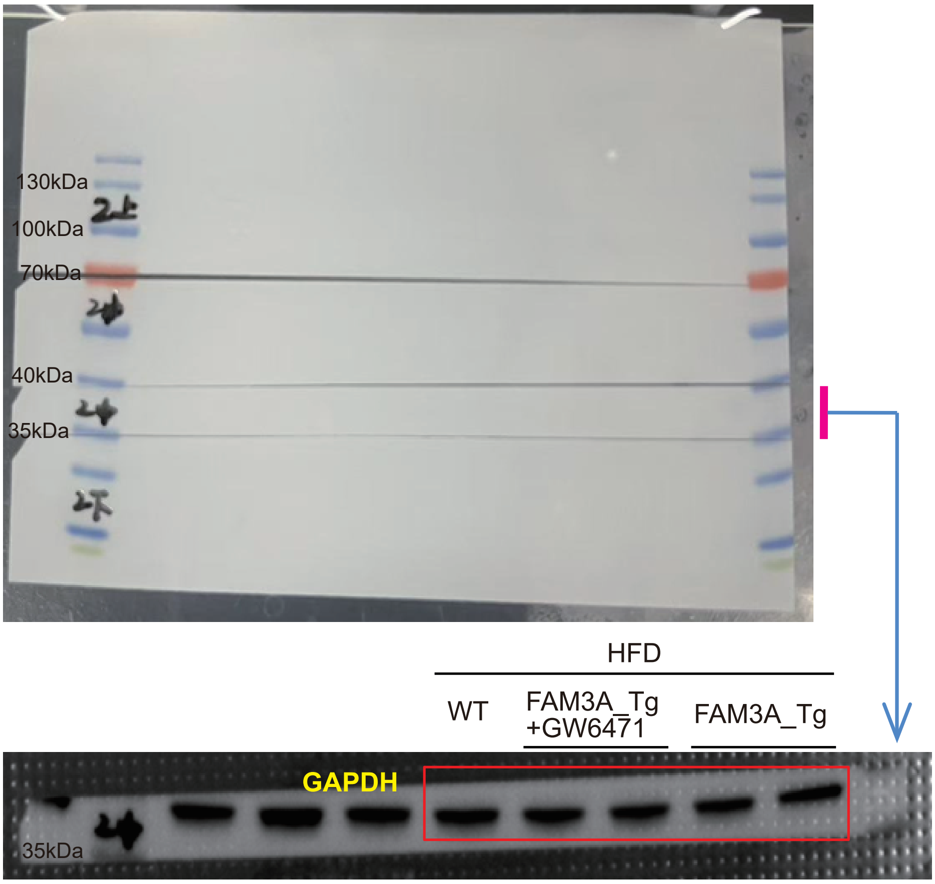
130kDa
100kDa
70kDa
40kDa
35kDa
HFD
WT
FAM3A_Tg
+GW6471
FAM3A_Tg
GAPDH
35kDa

Figure 6f

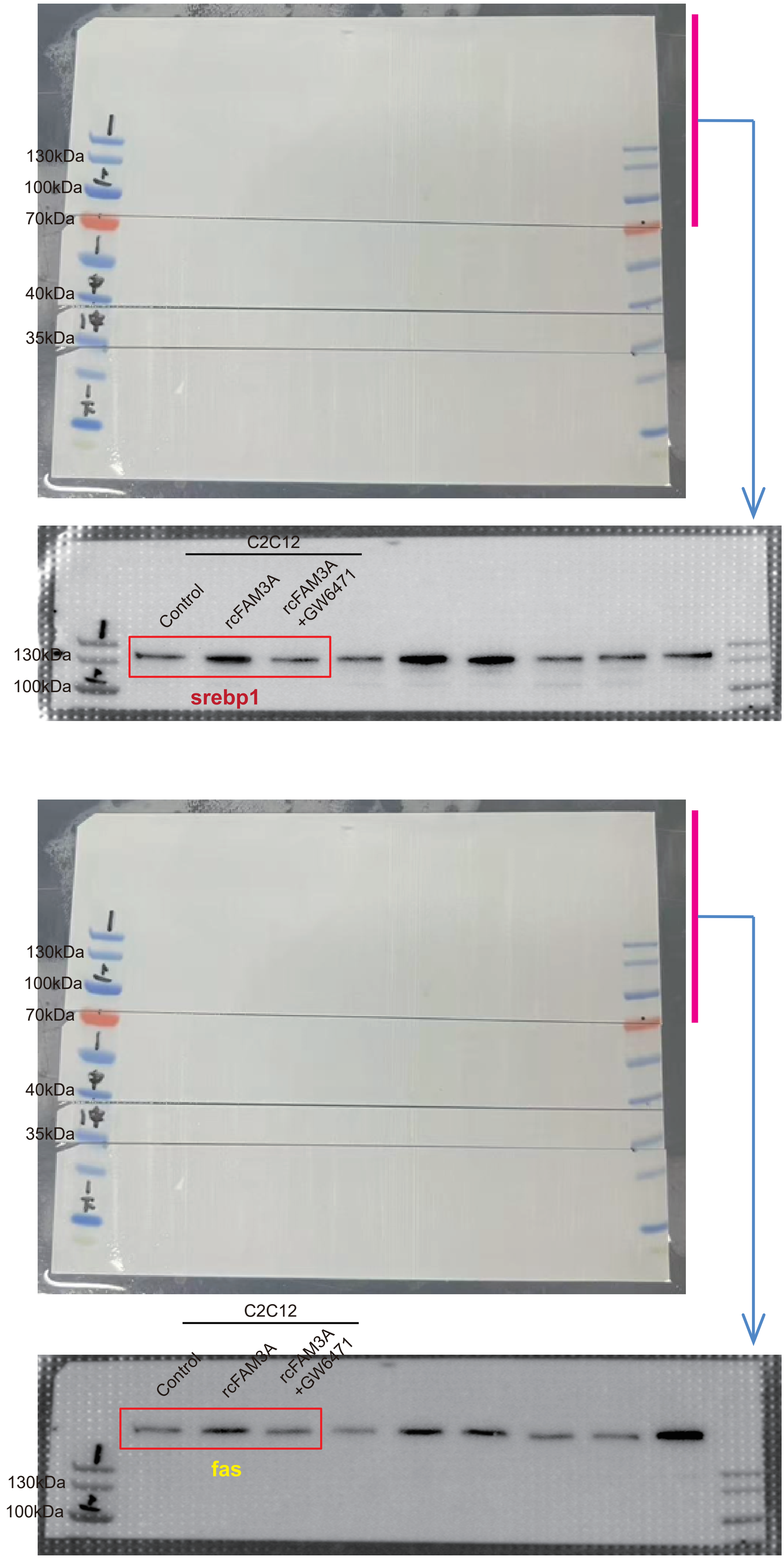

130kDa
100kDa
70kDa
40kDa
35kDa
C2C12
Control
rcFAM3A
rcFAM3A +GW6471
130kDa
100kDa
srebp1
130kDa
100kDa
70kDa
40kDa
35kDa
C2C12
Control
rcFAM3A
rcFAM3A +GW6471
fas
130kDa
100kDa

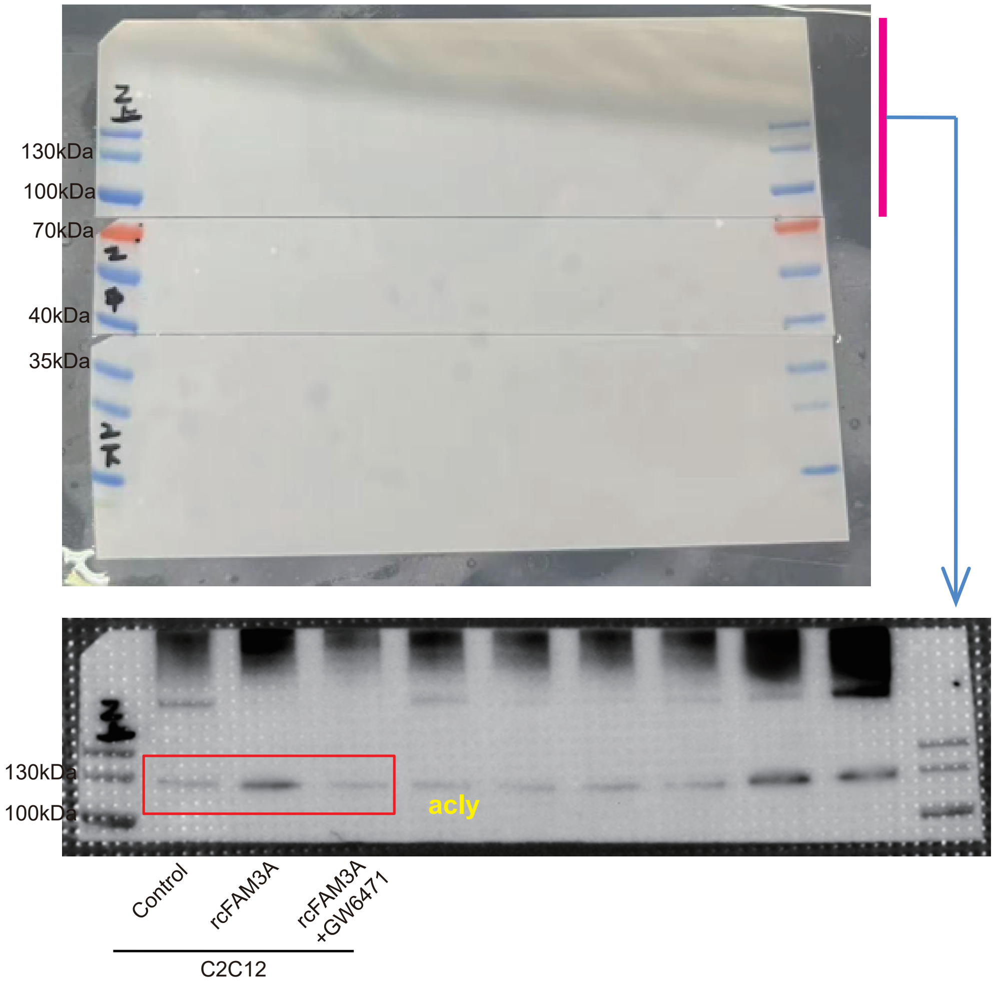
130kDa
100kDa
70kDa
40kDa
35kDa
130kDa
100kDa
acly
Control
rcFAM3A
rcFAM3A +GW6471
C2C12

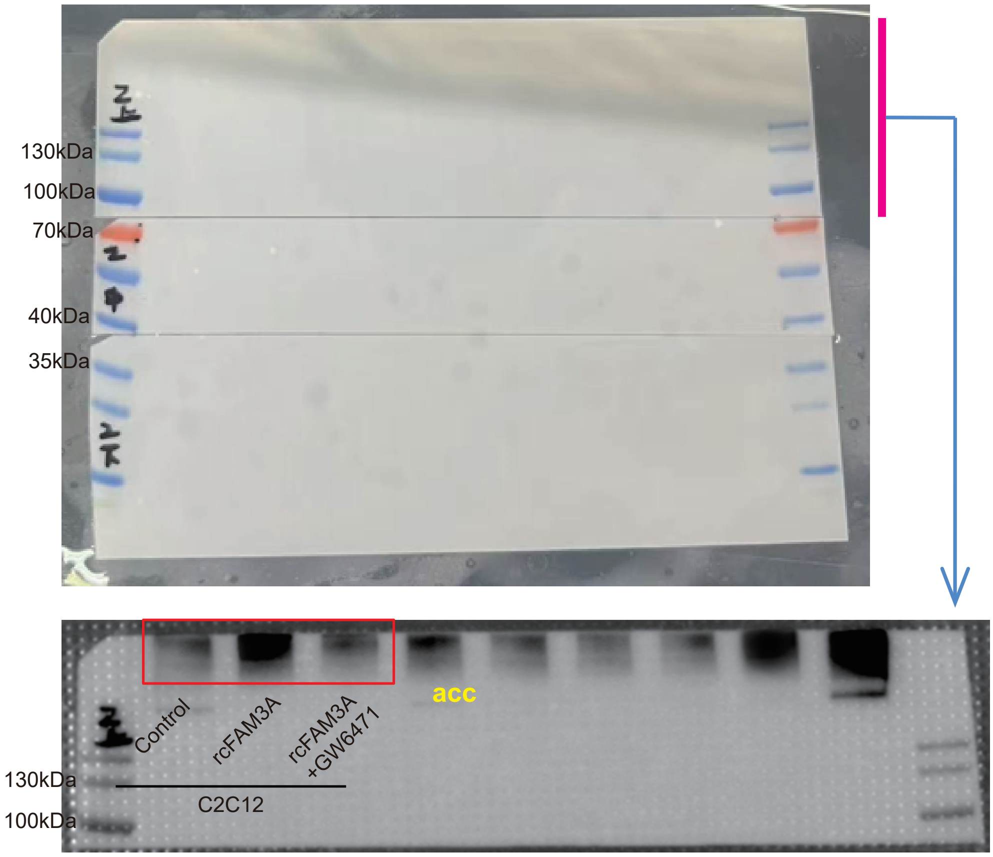
130kDa
100kDa
70kDa
40kDa
35kDa
acc
Control
rcFAM3A
rcFAM3A +GW6471
C2C12
130kDa
100kDa

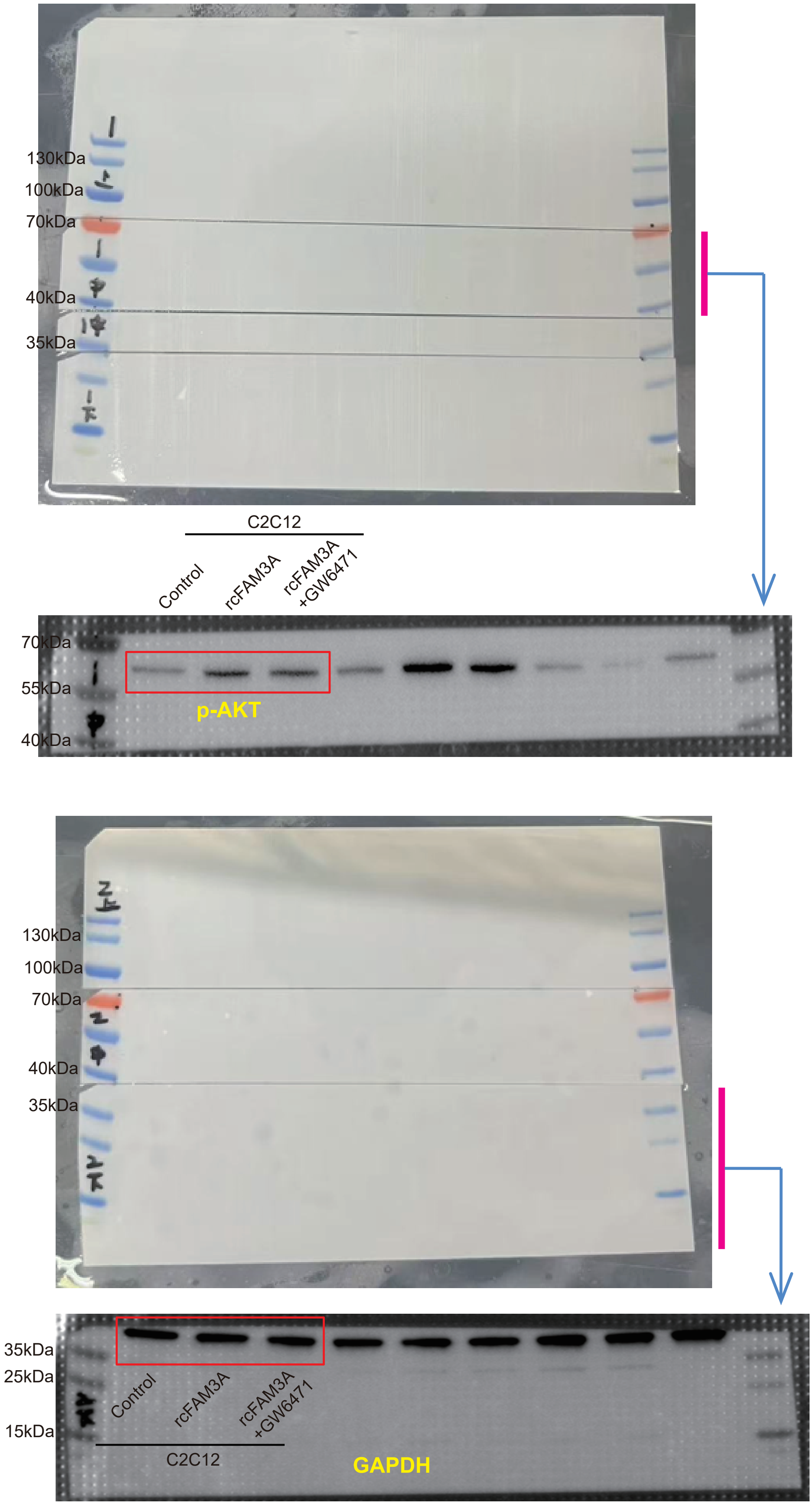

130kDa
100kDa
70kDa
40kDa
35kDa
C2C12
Control
rcFAM3A
rcFAM3A +GW6471
70kDa
55kDa
40kDa
p-AKT
130kDa
100kDa
70kDa
40kDa
35kDa
35kDa
25kDa
15kDa
Control
rcFAM3A
rcFAM3A +GW6471
C2C12
GAPDH

4. Figure 7

Figure 7d

HFD

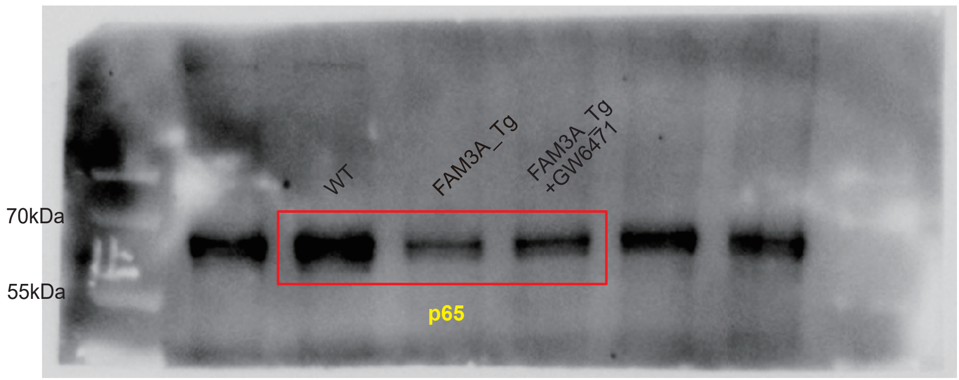

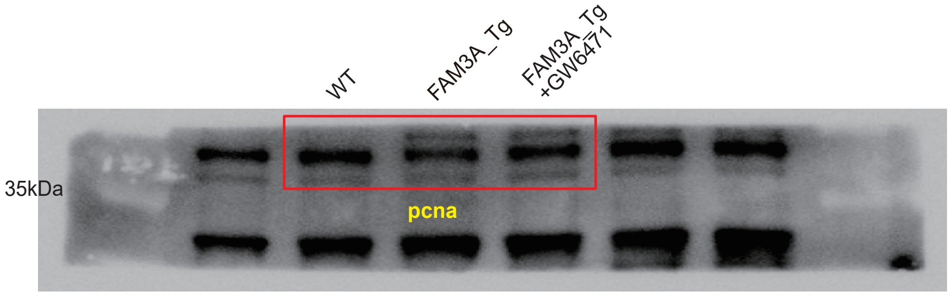

5. Figure 8

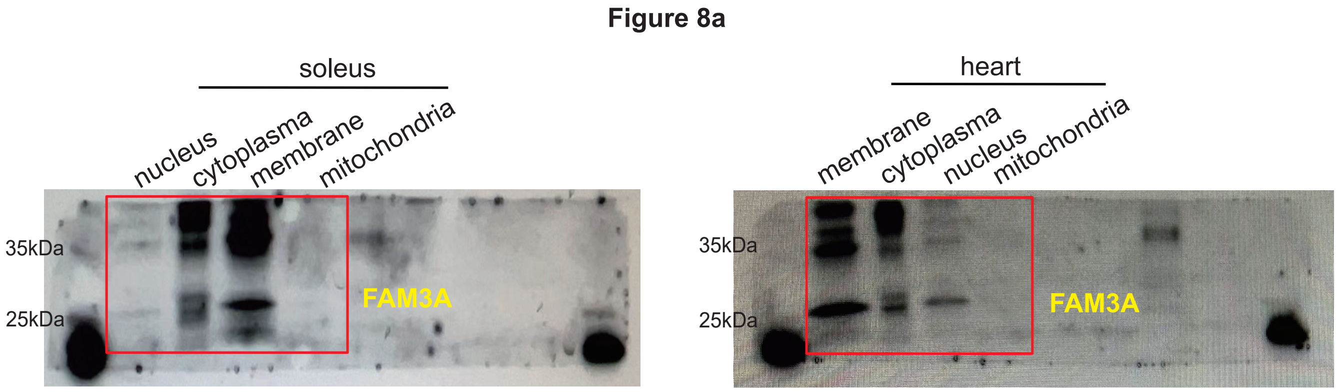

Figure 8a
soleus
nucleus
cytoplasma
membrane
mitochondria
35kDa
25kDa
FAM3A
heart
membrane
cytoplasma
nucleus
mitochondria
35kDa
25kDa
FAM3A

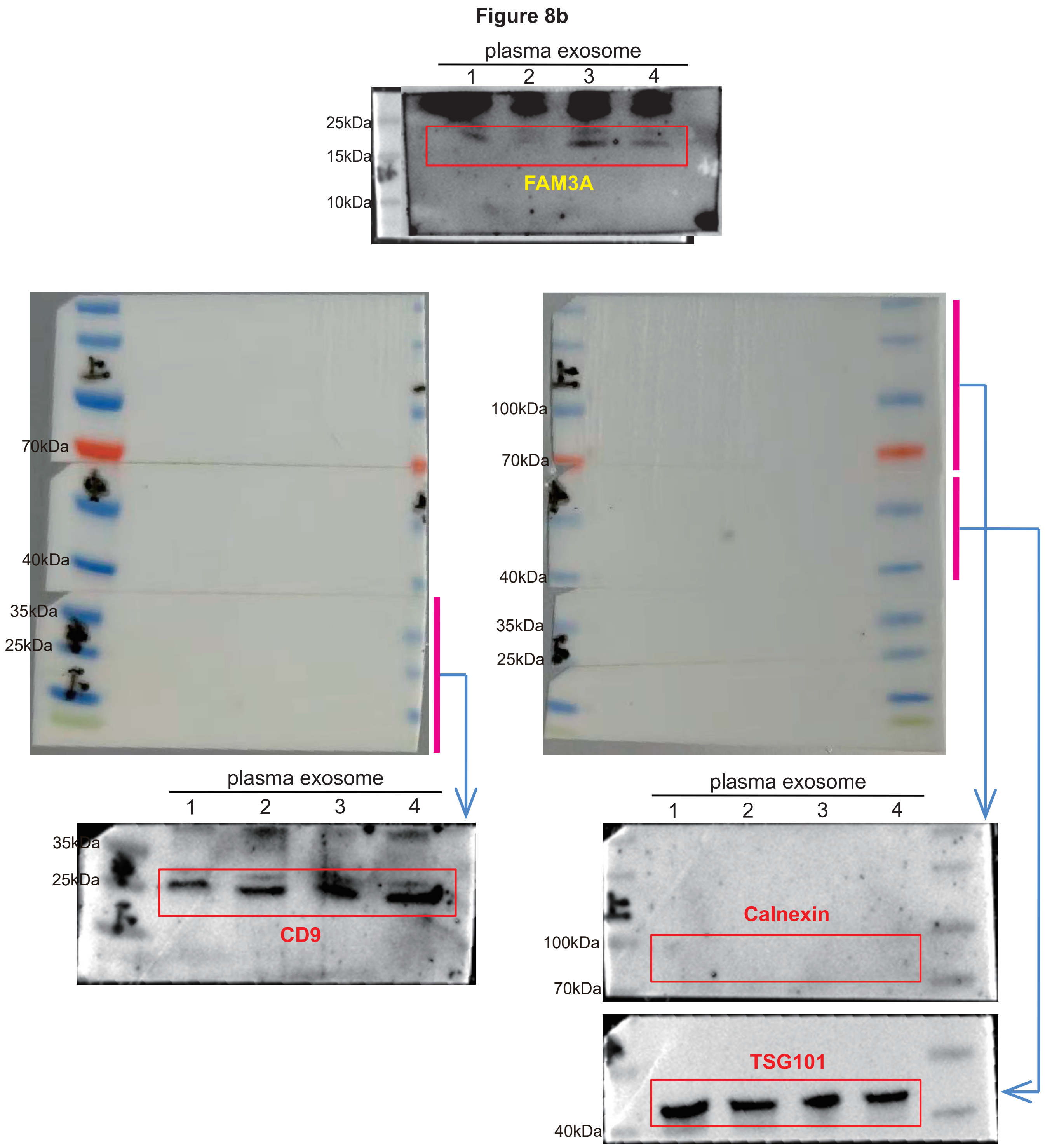

Figure 8b
plasma exosome
1
2
3
4
25kDa
15kDa
10kDa
FAM3A
70kDa
40kDa
35kDa
25kDa
100kDa
70kDa
40kDa
35kDa
25kDa
plasma exosome
1
2
3
4
35kDa
25kDa
CD9
plasma exosome
1
2
3
4
Calnexin
100kDa
70kDa
TSG101
40kDa

Figure 8c

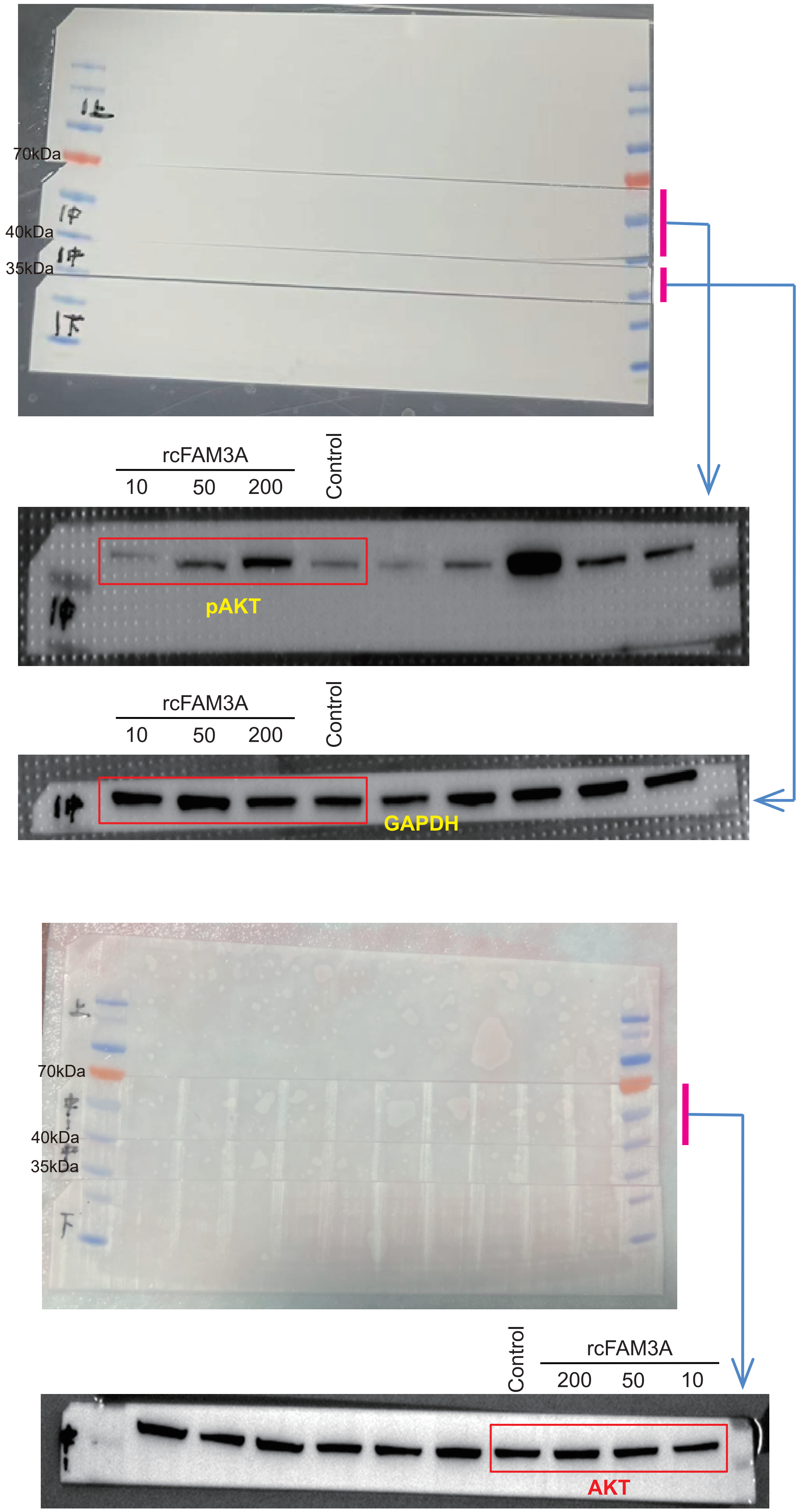
70kDa
40kDa
35kDa
rcFAM3A
10
50
200
Control
pAKT
rcFAM3A
10
50
200
Control
GAPDH
70kDa
40kDa
35kDa
Control
rcFAM3A
200
50
10
AKT

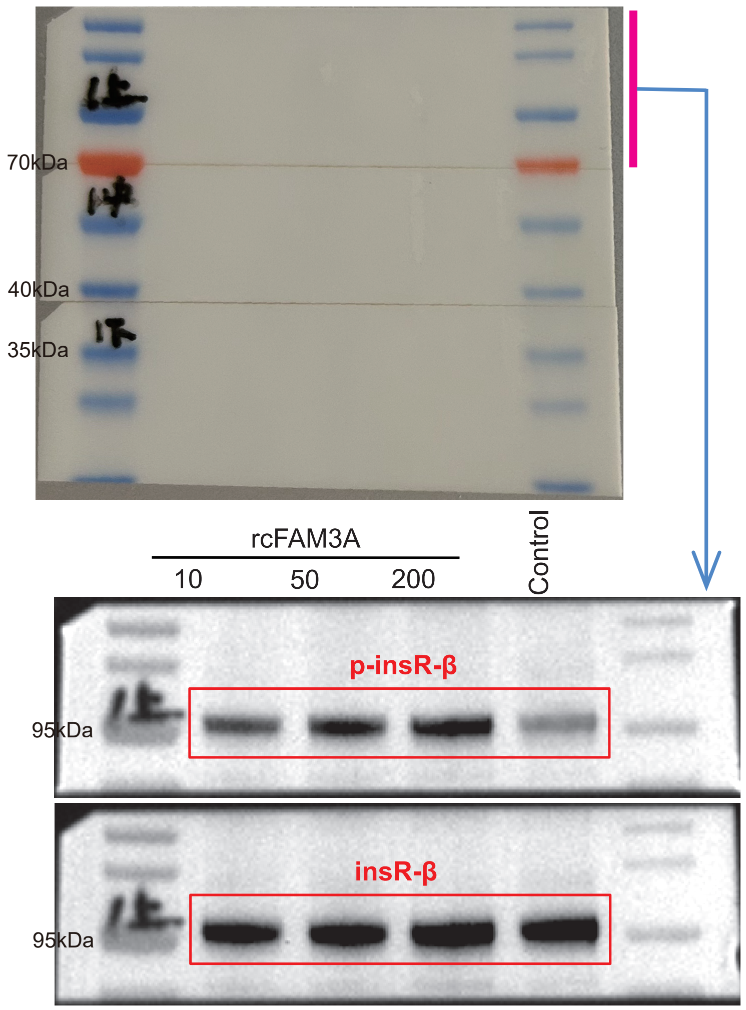
70kDa
40kDa
35kDa
rcFAM3A
10
50
200
Control
p-insR-β
95kDa
insR-β
95kDa

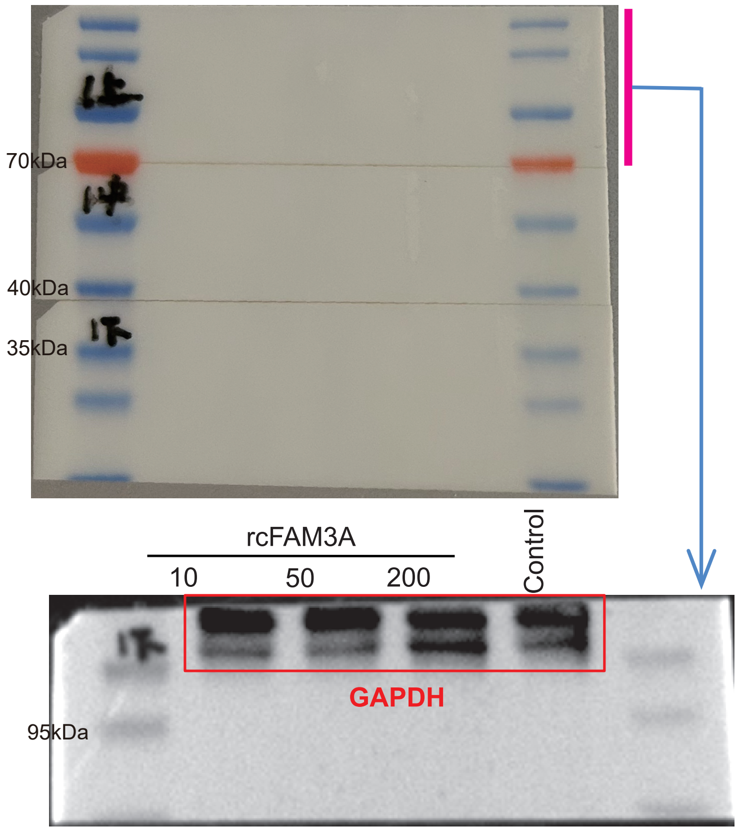
70kDa
40kDa
35kDa
rcFAM3A
10
50
200
Control
GAPDH
95kDa

Figure 8d

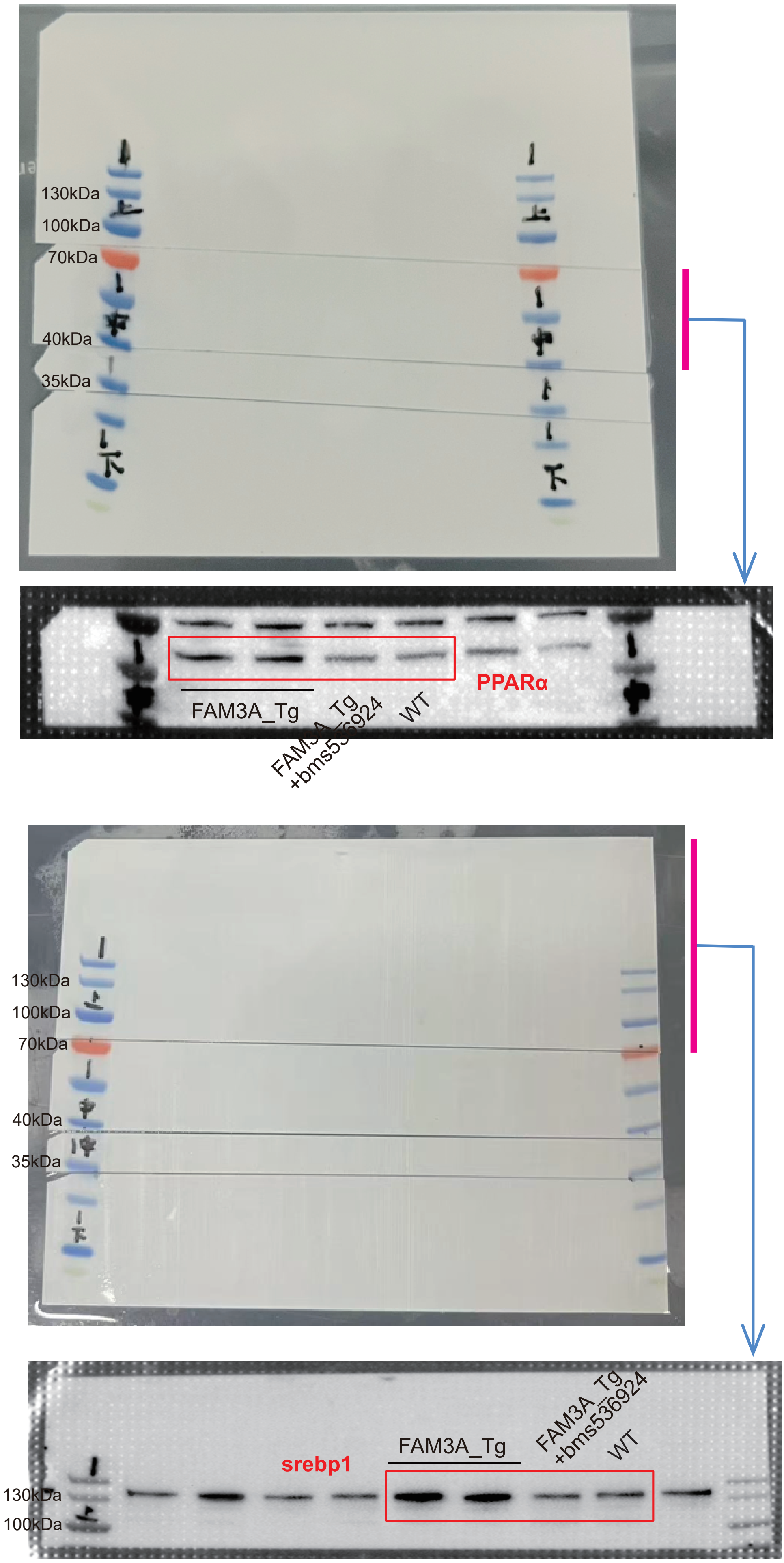
130kDa
100kDa
70kDa
40kDa
35kDa
PPARα
FAM3A_Tg
FAM3A_Tg +bms536924
WT
130kDa
100kDa
70kDa
40kDa
35kDa
srebp1
FAM3A_Tg
FAM3A_Tg +bms536924
WT
130kDa
100kDa

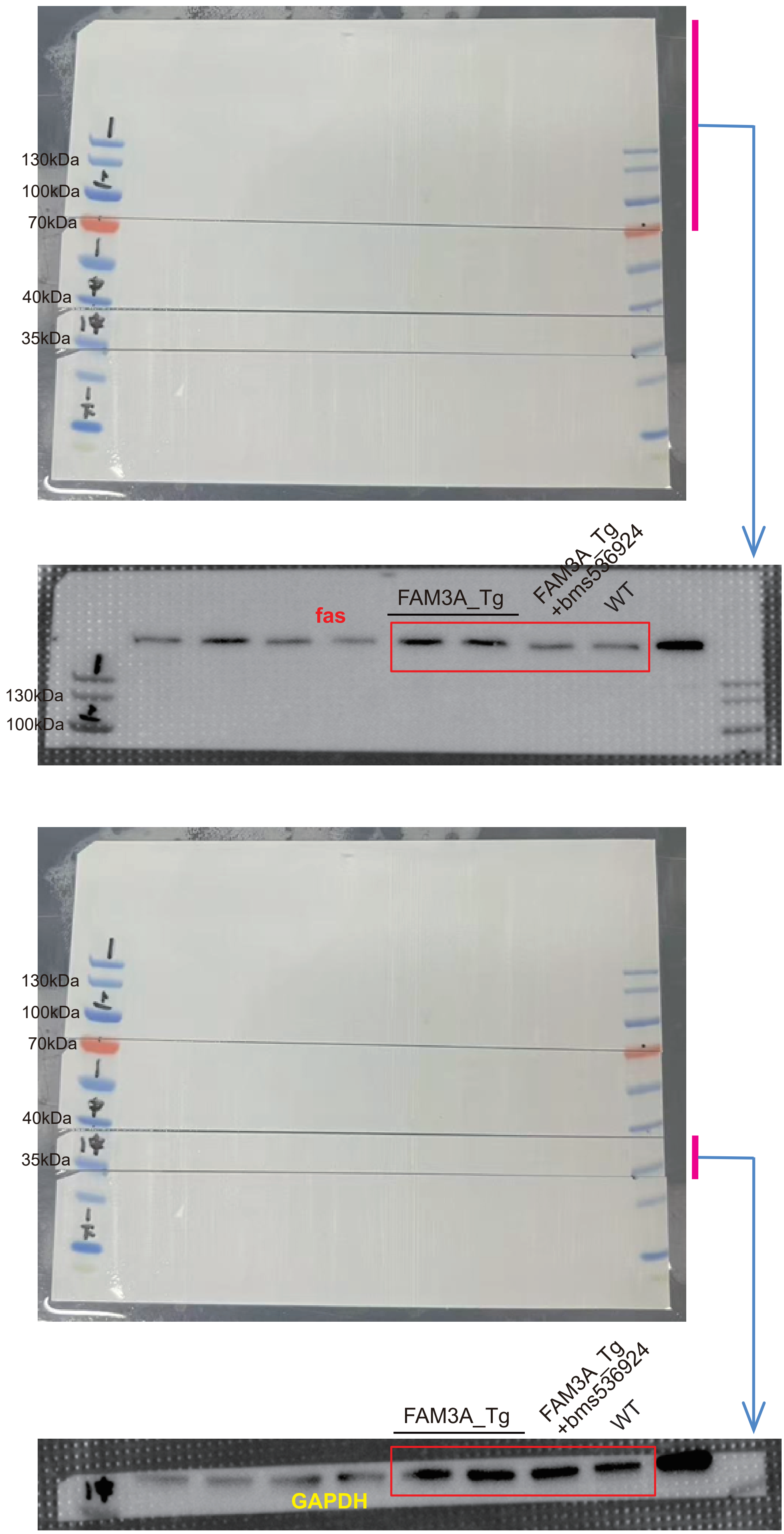
130kDa
100kDa
70kDa
40kDa
35kDa
fas
FAM3A_Tg
FAM3A_Tg
+bms536924
WT
130kDa
100kDa
130kDa
100kDa
70kDa
40kDa
35kDa
FAM3A_Tg
FAM3A_Tg
+bms536924
WT
GAPDH

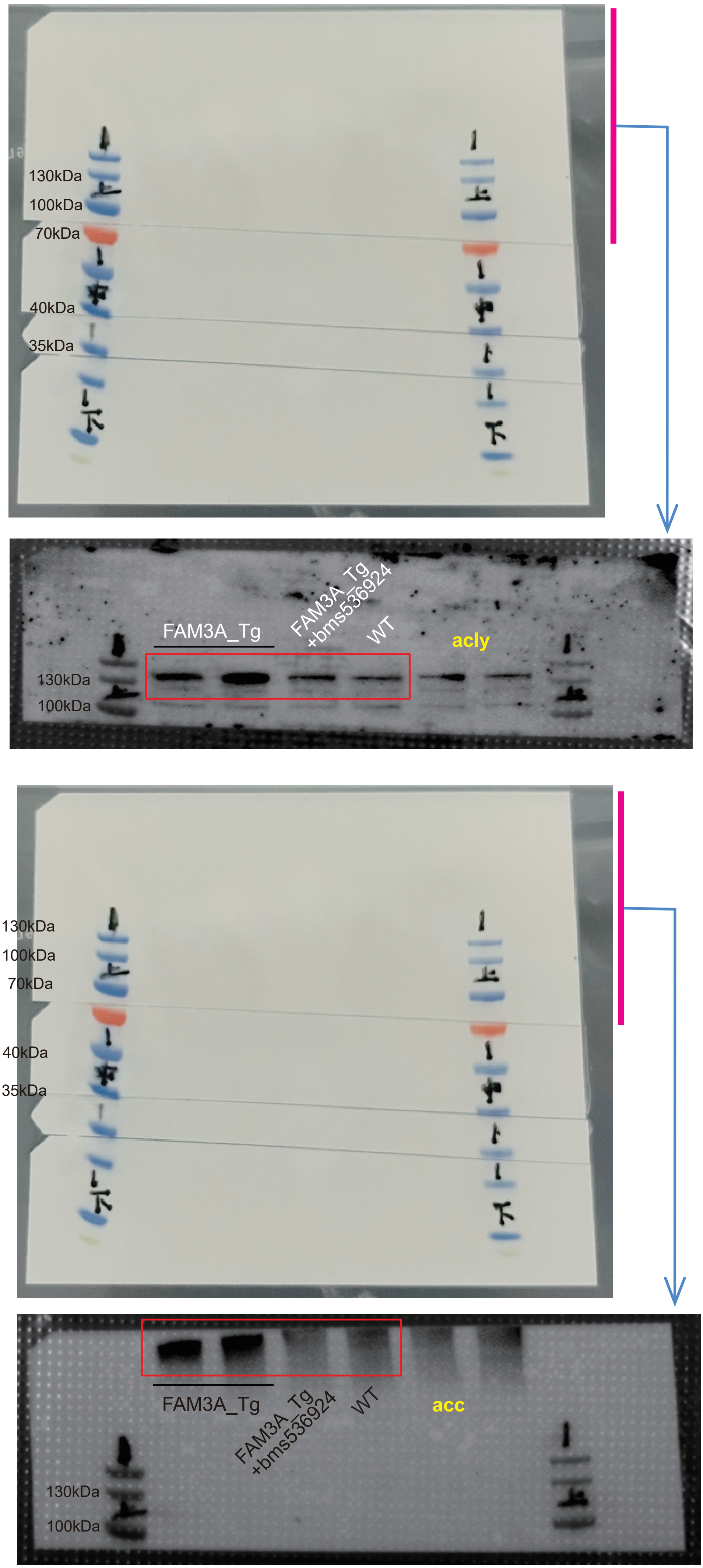

130kDa
100kDa
70kDa
40kDa
35kDa
FAM3A_Tg
FAM3A_Tg +bms536924
WT
acly
130kDa
100kDa
130kDa
100kDa
70kDa
40kDa
35kDa
FAM3A_Tg
FAM3A_Tg +bms536924
WT
acc
130kDa
100kDa

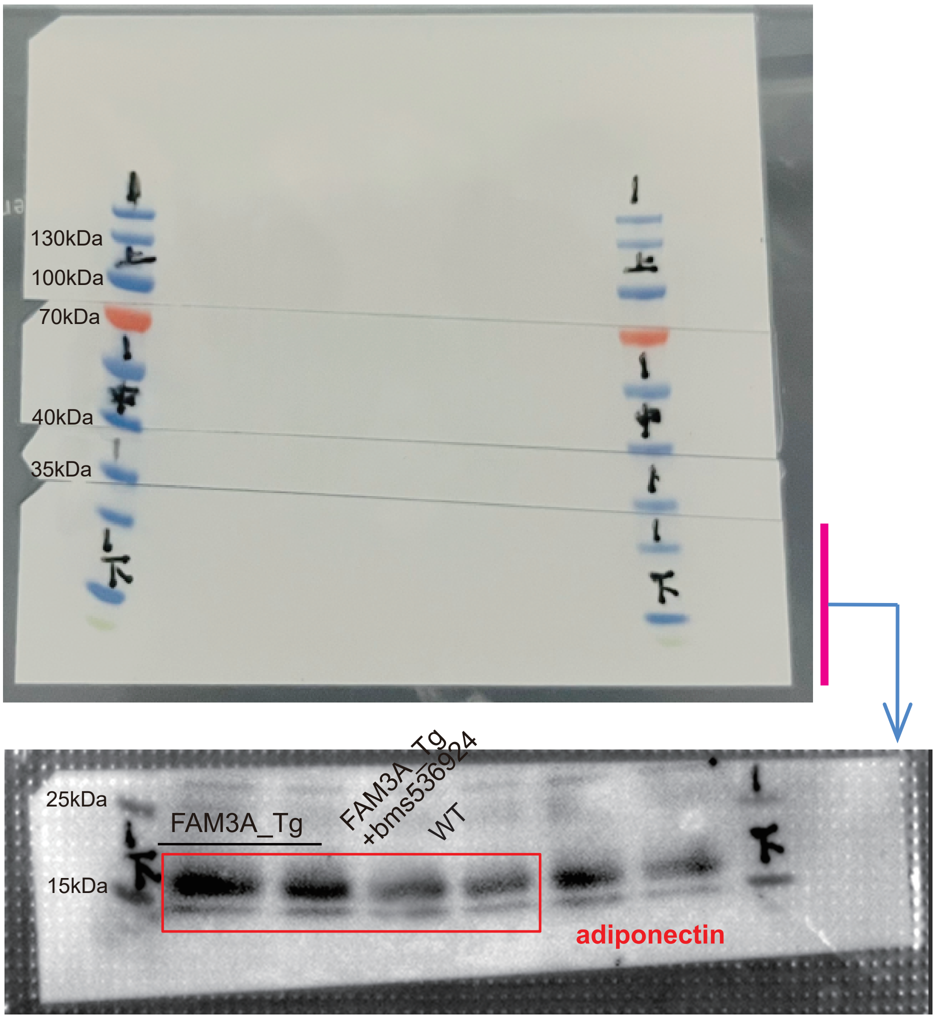
130kDa
100kDa
70kDa
40kDa
35kDa
25kDa
15kDa
FAM3A_Tg
FAM3A_Tg
+bms536924
WT
adiponectin

Figure 8e

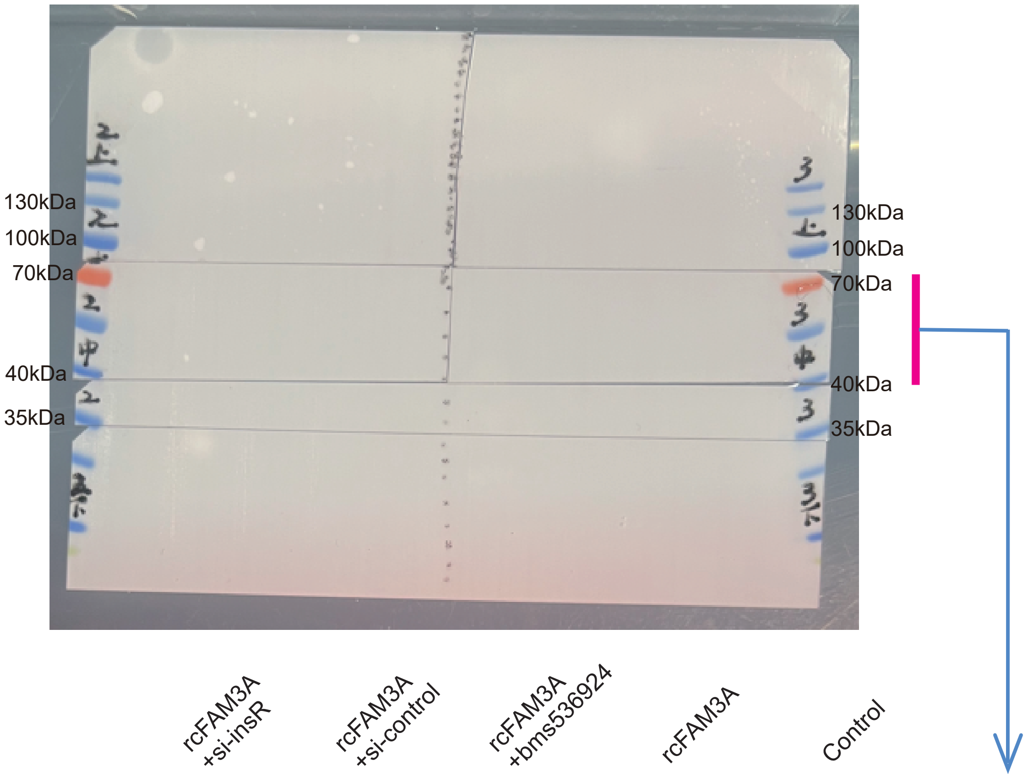
130kDa
100kDa
70kDa
40kDa
35kDa
130kDa
100kDa
70kDa
40kDa
35kDa

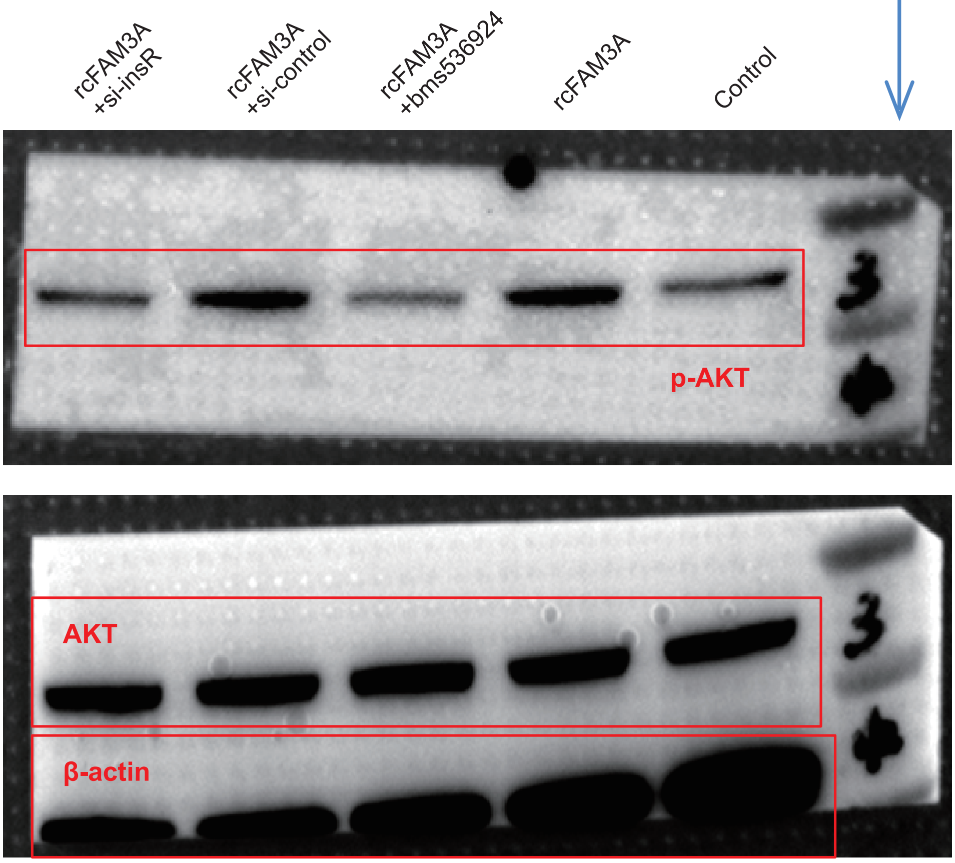
rcFAM3A +si-insR
rcFAM3A +si-control
rcFAM3A +bms536924
rcFAM3A
Control
p-AKT
AKT
β-actin

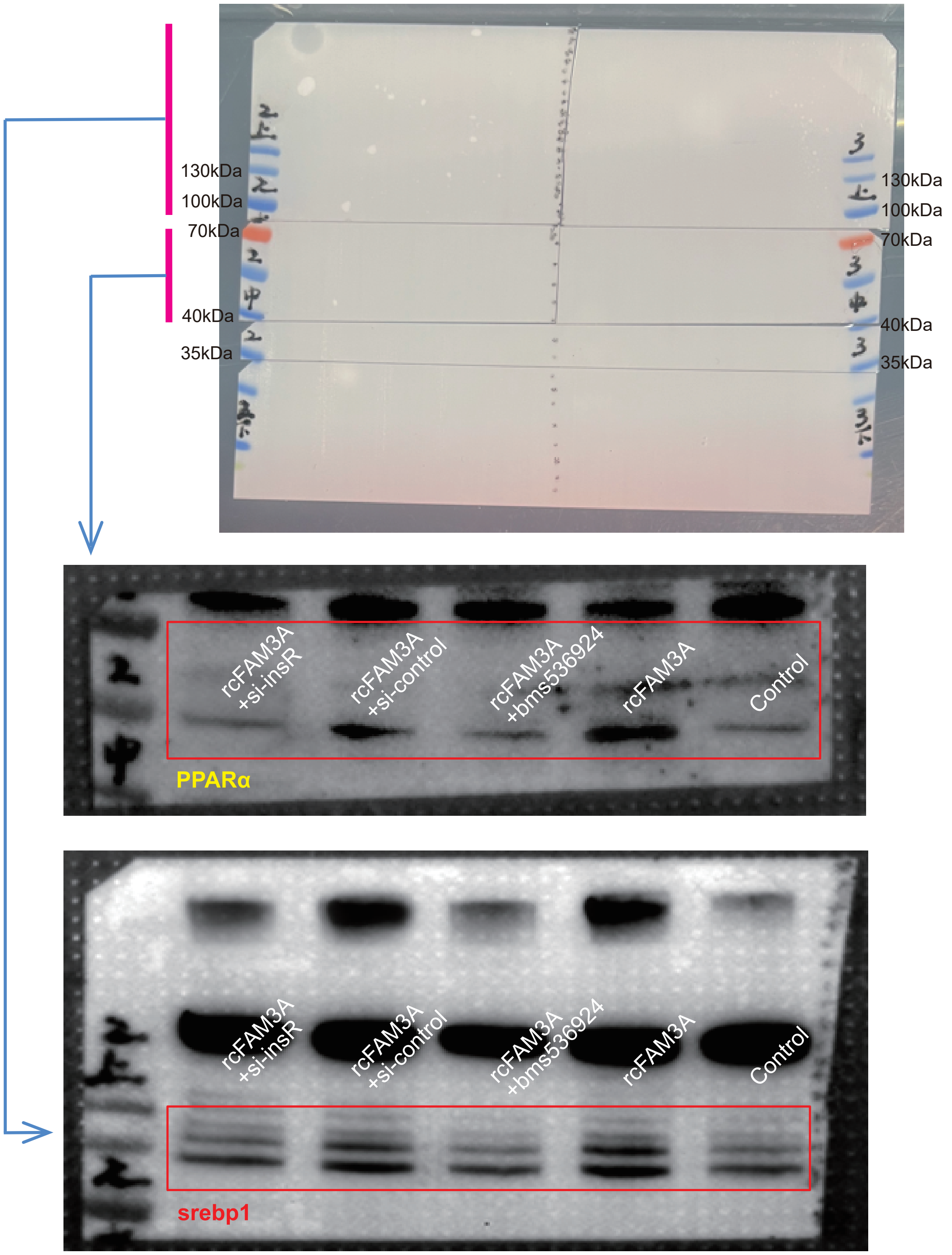
130kDa
100kDa
70kDa
40kDa
35kDa
130kDa
100kDa
70kDa
40kDa
35kDa
rcFAM3A +si-insR
rcFAM3A +si-control
rcFAM3A +bms536924
rcFAM3A
Control
PPARα
rcFAM3A +si-insR
rcFAM3A +si-control
rcFAM3A +bms536924
rcFAM3A
Control
srebp1

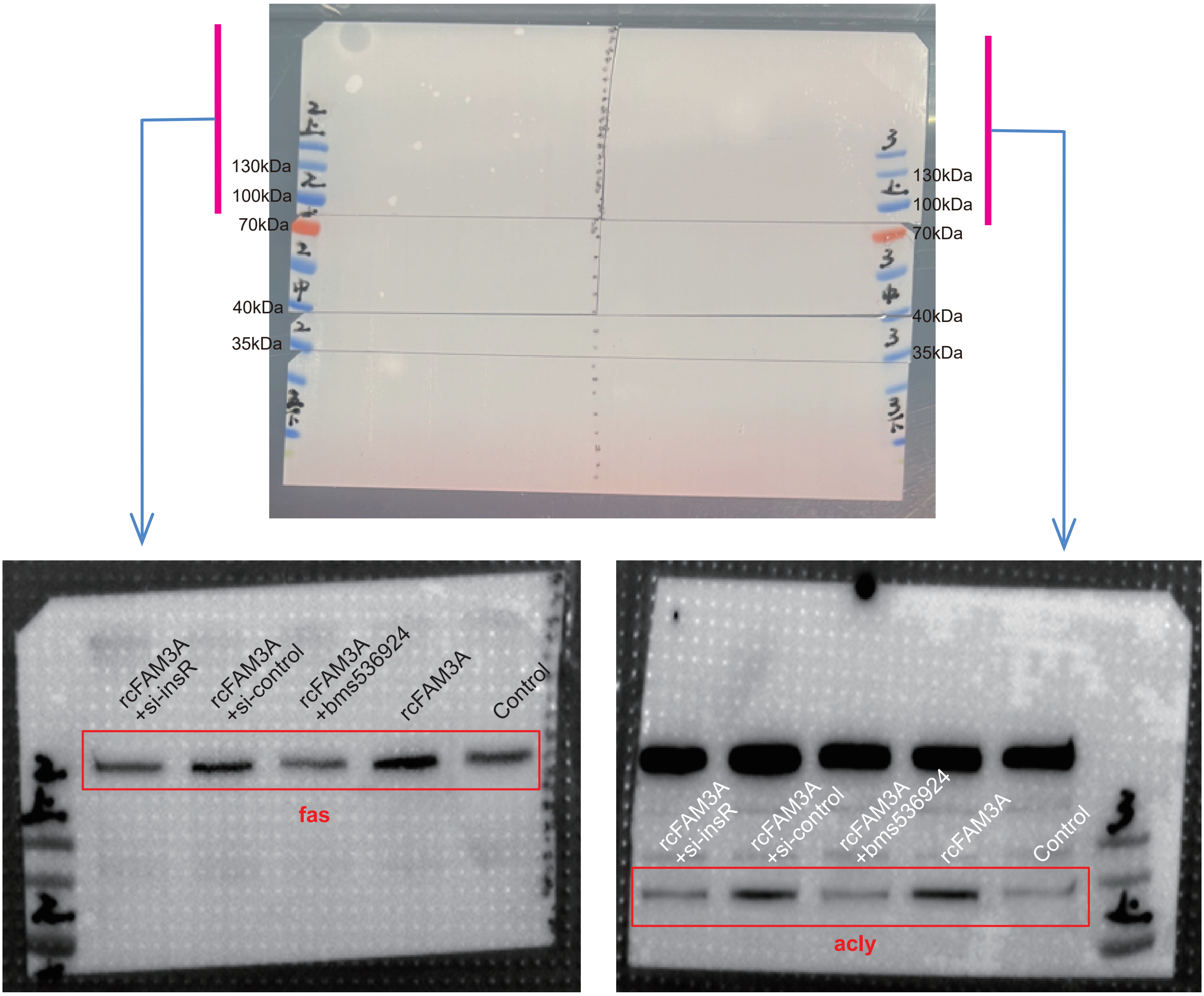
130kDa
100kDa
70kDa
40kDa
35kDa
130kDa
100kDa
70kDa
40kDa
35kDa
rcFAM3A +si-insR
rcFAM3A +si-control
rcFAM3A +bms536924
rcFAM3A
Control
fas
rcFAM3A +si-insR
rcFAM3A +si-control
rcFAM3A +bms536924
rcFAM3A
Control
acly

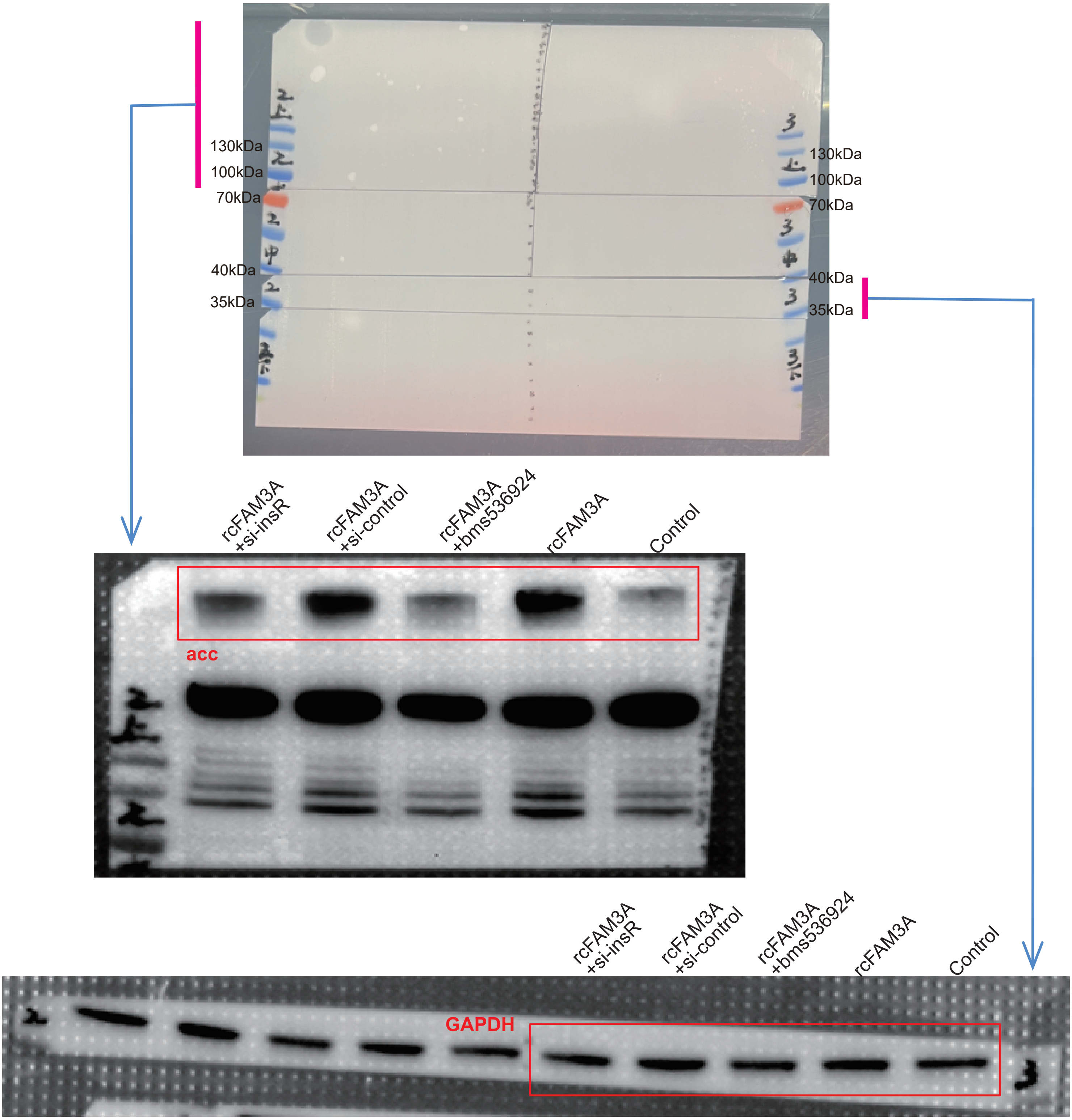
130kDa
100kDa
70kDa
40kDa
35kDa
130kDa
100kDa
70kDa
40kDa
35kDa
rcFAM3A +si-insR
rcFAM3A +si-control
rcFAM3A +bms536924
rcFAM3A
Control
acc
rcFAM3A +si-insR
rcFAM3A +si-control
rcFAM3A +bms536924
rcFAM3A
Control
GAPDH

6. Supplementary Figure 3

Supplementary Figure 3a

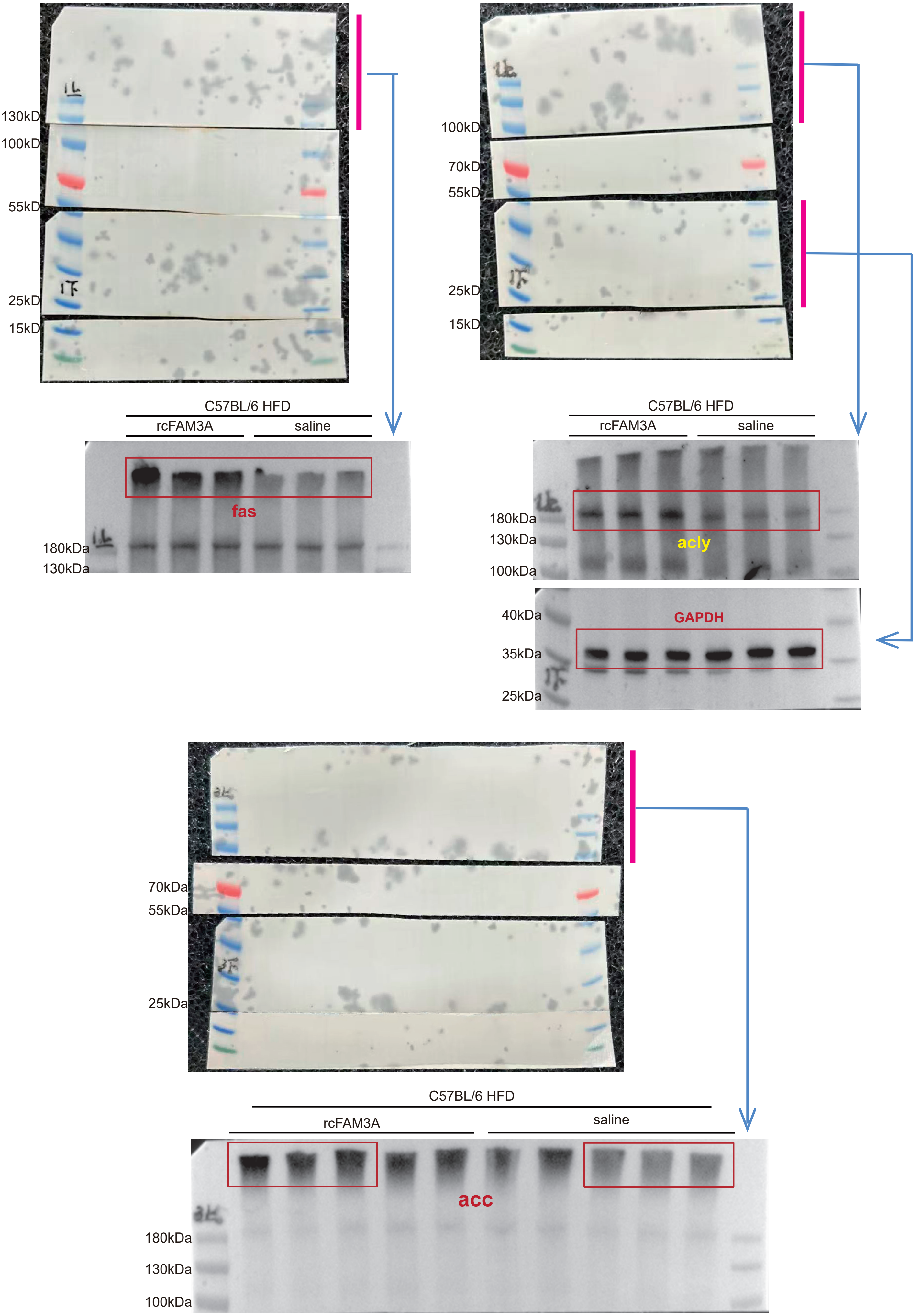

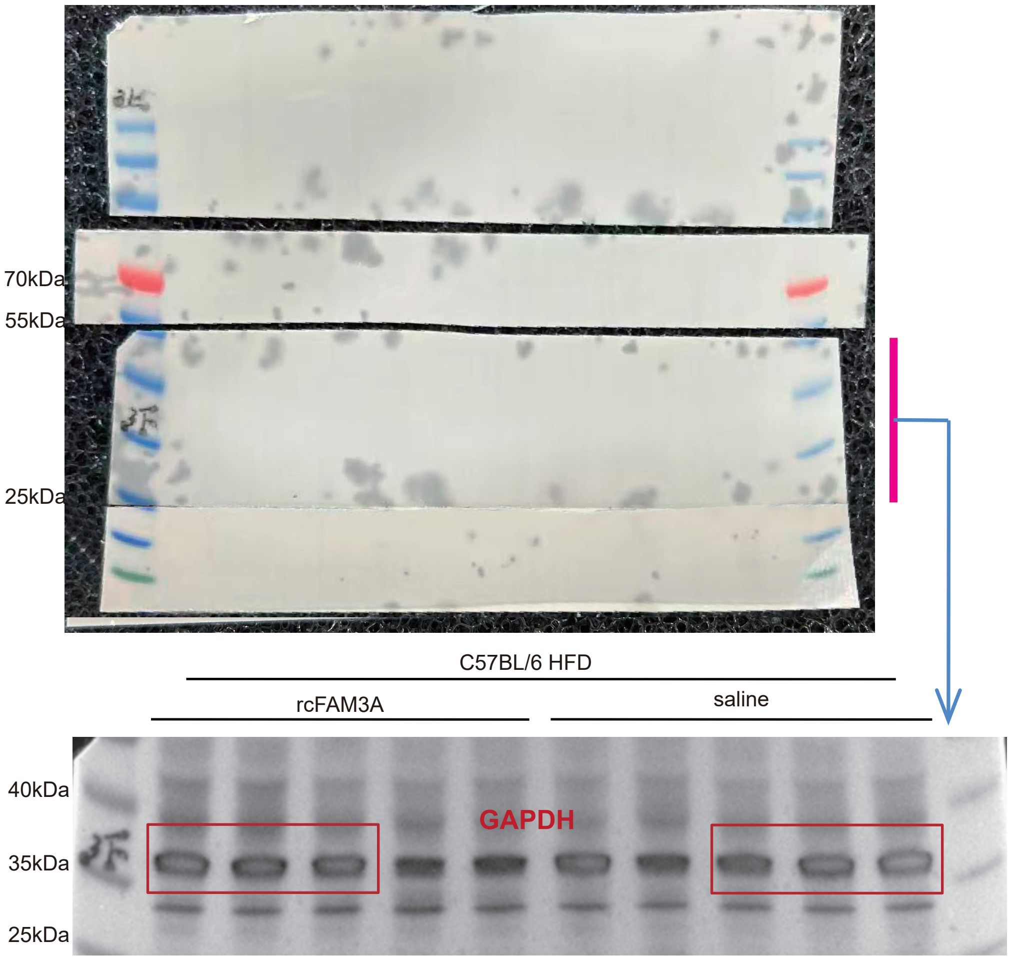
70kDa
55kDa
25kDa
C57BL/6 HFD
rcFAM3A
saline
40kDa
GAPDH
35kDa
25kDa

7. Supplementary Figure 9

Supplementary Figure 9b

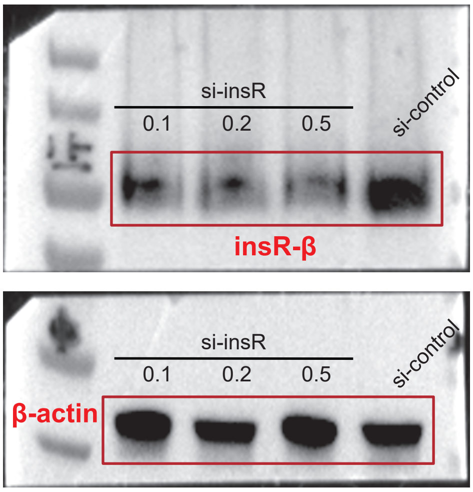

Supplementary Figure 9c

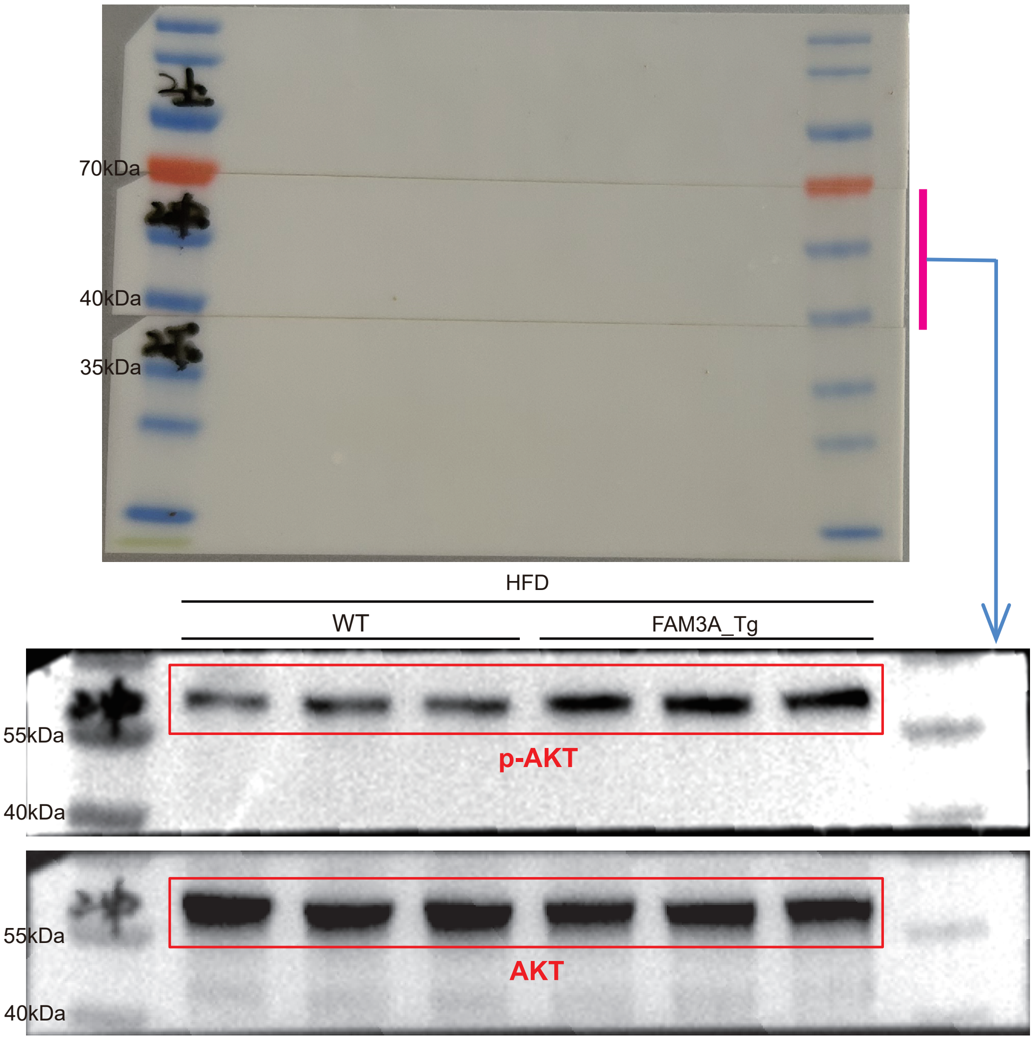

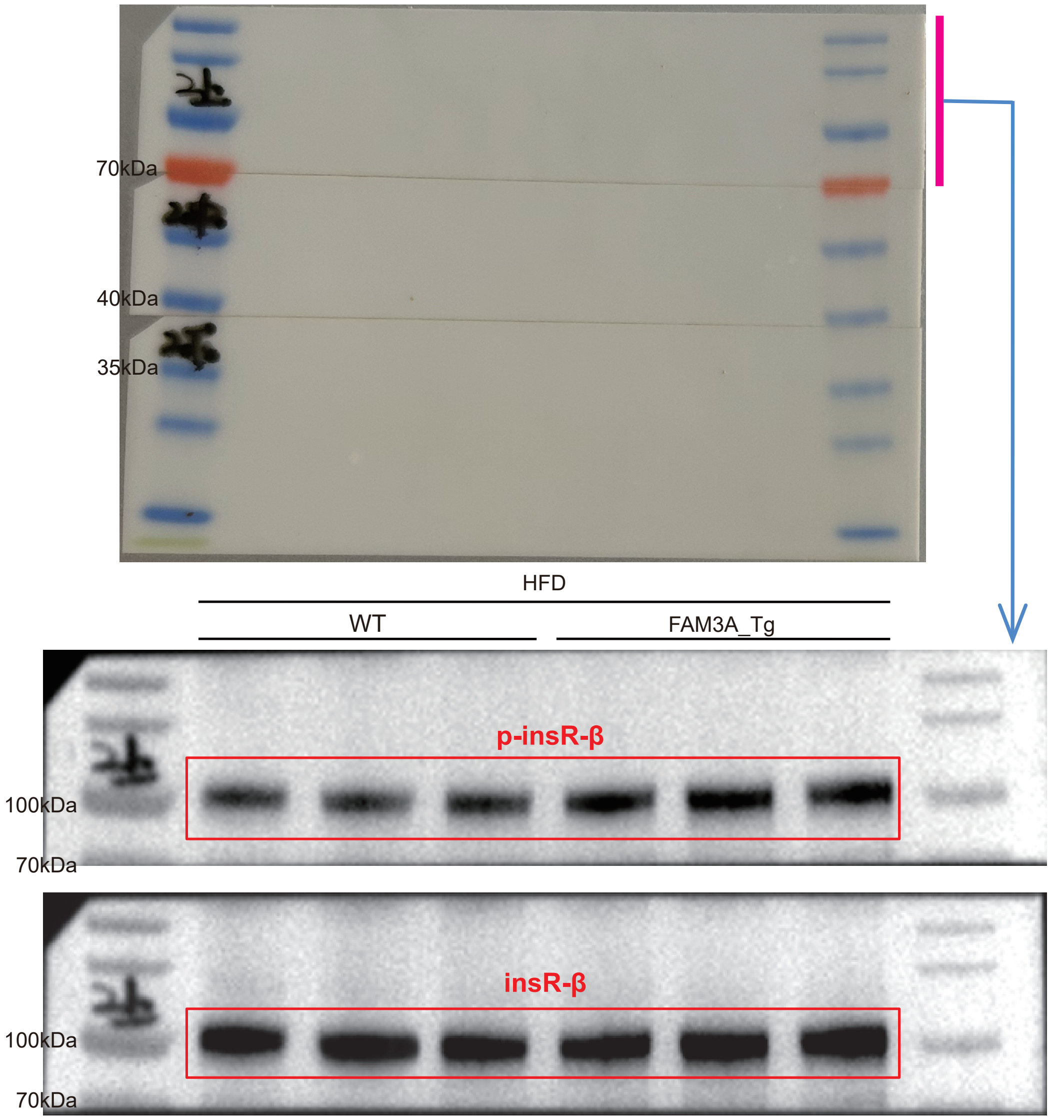
70kDa
40kDa
35kDa
HFD
WT
FAM3A_Tg
p-insR-β
100kDa
70kDa
insR-β
100kDa
70kDa

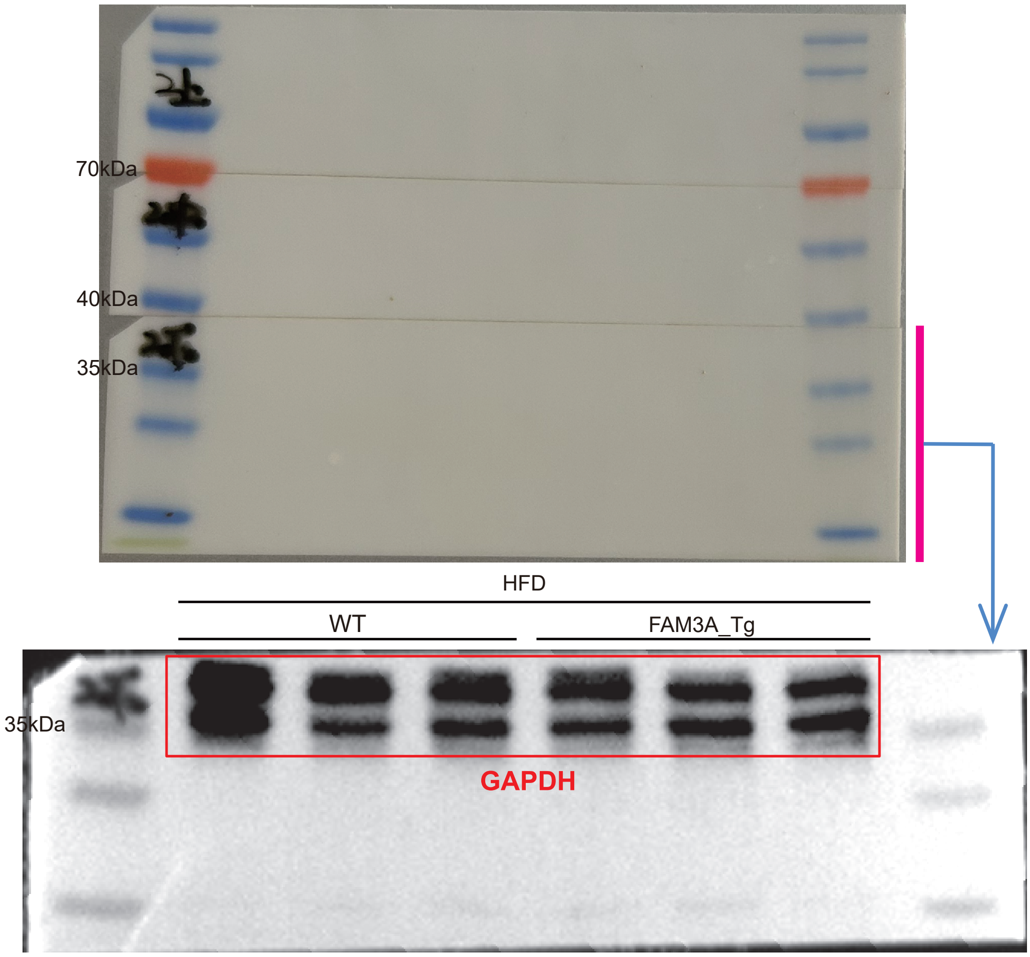
70kDa
40kDa
35kDa
HFD
WT
FAM3A_Tg
35kDa
GAPDH
